# Adamantane-Substituted Purines and Their β-Cyclodextrin Complexes: Synthesis and Biological Activity

**DOI:** 10.3390/ijms222312675

**Published:** 2021-11-24

**Authors:** Michal Rouchal, Jana Rudolfová, Vladimír Kryštof, Veronika Vojáčková, Richard Čmelík, Robert Vícha

**Affiliations:** 1 Department of Chemistry, Faculty of Technology, Tomas Bata University in Zlín, Vavrečkova 275, 760 01 Zlín, Czech Republic; rouchal@utb.cz (M.R.); jhermanova@utb.cz (J.R.); 2 Department of Experimental Biology, Palacký University, Šlechtitelů 27, 783 71 Olomouc, Czech Republic; vladimir.krystof@upol.cz (V.K.); veronika.vojackova@upol.cz (V.V.); 3 Institute of Analytical Chemistry of the Czech Academy of Sciences, Veveří 97, 602 00 Brno, Czech Republic; cmelik@iach.cz

**Keywords:** adamantane, 2,6,9-trisubstituted purine, cyclin-dependent kinase, cytotoxicity, β-cyclodextrin, molecular docking

## Abstract

Cyclin-dependent kinases (CDKs) play an important role in the cell-division cycle. Synthetic inhibitors of CDKs are based on 2,6,9-trisubstituted purines and are developed as potential anticancer drugs; however, they have low solubility in water. In this study, we proved that the pharmaco-chemical properties of purine-based inhibitors can be improved by appropriate substitution with the adamantane moiety. We prepared ten new purine derivatives with adamantane skeletons that were linked at position 6 using phenylene spacers of variable geometry and polarity. We demonstrated that the adamantane skeleton does not compromise the biological activity, and some of the new purines displayed even higher inhibition activity towards CDK2/cyclin E than the parental compounds. These findings were supported by a docking study, which showed an adamantane scaffold inside the binding pocket participating in the complex stabilisation with non-polar interactions. In addition, we demonstrated that β-cyclodextrin (CD) increases the drug’s solubility in water, although this is at the cost of reducing the biochemical and cellular effect. Most likely, the drug concentration, which is necessary for target engagement, was decreased by competitive drug binding within the complex with β-CD.

## 1. Introduction

Cyclin-dependent kinases (CDKs) are a family of serine/threonine protein kinases that play a crucial role in controlling the cell division cycle in all eukaryotic cells. The activation of CDKs, which represent the catalytic subunit in the heterodimeric complex, usually requires association with a regulatory cyclin subunit [1]. It was found that the excessive activity of cyclins or loss of expression of endogenous CDK protein inhibitors (Cip/Kip or INK4), which results in the deregulation of CDKs activity, is frequently observed in human malignancies. This initiated extensive search for the low-molecular-weight inhibitors of CDKs [2,3]. Over the past three decades, a number of ATP-competitive CDK inhibitors, differing in their chemical structure or selectivity, have been developed. To date, more than 20 CDK inhibitors have been advanced to some stage of clinical evaluation [4]. In 2015, the low-molecular-weight inhibitor of CDK4 and CDK6 palbociclib was approved by the United States Food and Drug Administration for the treatment of breast cancer in clinical use. This drug was followed by the next two CDK4/6 inhibitors, ribociclib and abemaciclib [5].

**Figure 1:**
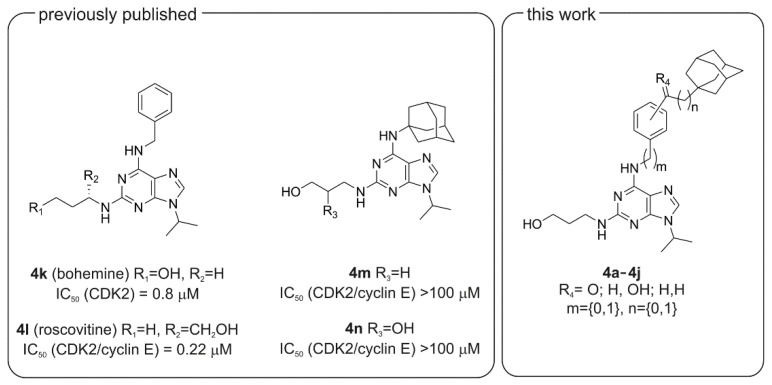
Purine derivatives considered in this work. The inhibitory concentration values (IC_50_) are taken from previously published papers (**4k** [14]; **4l** [15]; **4m**, **4n** [14,16]).

Purines, appropriately substituted at C2, C6 and C9 centres, represent a class of compounds that strongly inhibit CDKs or other kinases [6]. Structural modifications of olomoucine, one of the first selective CDK inhibitors [7], provided a number of 2,6,9-trisubstituted purines that are highly active towards CDKs, such as bohemine, roscovitine, purvalanol A and olomoucine II (Figure 1) [8,9,10]. More recently, a novel series of 2-substituted-6-biarylmethylamino-9-cyclopentylpurine derivatives exhibiting strong CDK inhibitory activity and antiproliferative effects was described [11]. The success of roscovitine as an anticancer drug candidate even inspired the synthesis and biological evaluation of several types of bioisosteres [12,13].

Adamantane (tricyclo[3.3.1.1^3,7^]decane) is a polycyclic hydrocarbon present in a number of compounds synthesised as potential therapeutics. They act as agents that are antiviral [17,18,19], hypoglycemic [20], antitumor [21,22] or antimalarial [23] substances for hypertension and vascular inflammation treatment [24,25], potential antituberculosis candidates [26] and as cannabinoid receptor ligands [27]. Due to its high lipophilicity, the adamantane scaffold is usually combined with the known pharmacophores to prepare biologically active compounds with a better pharmacological profile. At present, seven compounds bearing the adamantane moiety are in clinical use [28,29,30]. One of the most interesting properties of the adamantane scaffold is its ability to form non-covalent inclusion complexes with several types of molecular containers (e.g., cyclodextrins [CDs] or cucurbit[*n*]urils).

CDs are water-soluble, non-toxic chemical substances that are easily obtained by the biotransformation of starch. CDs are used across all industries, but most frequently they are applied in pharmacy, medicine, biotechnology, agriculture, food chemistry, cosmetics and textile chemistry [31]. Natural CDs, generally known as α-, β- and γ-, are cyclic oligosaccharides consisting of six to eight glucose units, which are linked via α-(1,4)-glycosidic bonds. CDs have a truncated cone shape with a hydrophobic cavity interior in which a number of lipophilic guest molecules can be included through non-covalent interactions [32]. The formation of host–guest complexes of natural and/or modified CDs (prepared with the transformation of hydroxyl groups) with potential drug candidates has been extensively studied, with the aim of enhancing the water solubility, biological activity and characteristics of pharmaceutical interests, such as, for example, stability and/or bioavailability [33,34,35,36,37,38,39].

In this work, a series of novel 2,6,9-trisubstitued purines bearing adamantylated aromatic amines in position 6 was prepared (Figure 1). The formation of host–guest complexes between synthesised trisubstituted purines and β-CD was studied. The new compounds, as well as their mixtures with β-CD, were assayed for their enzymatic and cytotoxic activity.

## 2. Results and Discussion

### 2.1. Chemistry

**Scheme 1:**
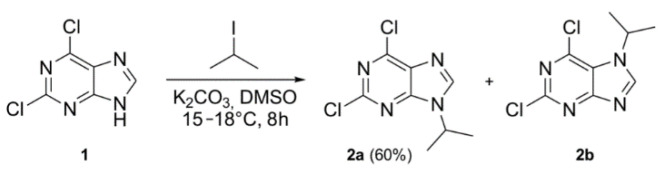
Alkylation of 2,6-dichloro-9*H*-purine.

**Scheme 2:**
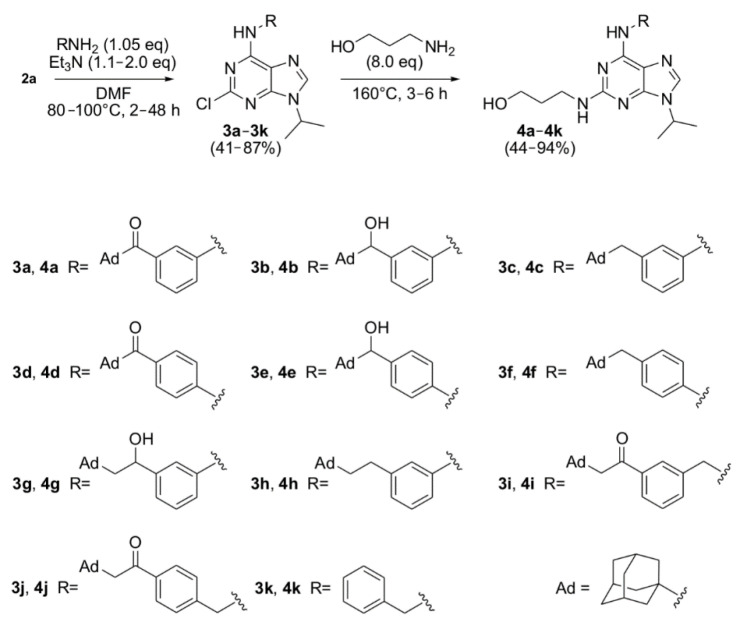
Synthesis of 2,6,9-trisubstituted purines bearing the adamantane moiety.

A novel series of 2,6,9-trisubstituted purines bearing the adamantane moiety was prepared with a three-step procedure outlined in Scheme 1 and Scheme 2.

The alkylation of the commercially available 2,6-dichloro-9*H*-purine (**1**) was carried out with 2-iodopropane and potassium carbonate in dimethyl sulfoxide (DMSO) between 15 and 18 °C (Scheme 1), according to the previously published procedure [40]. Although the authors of the original paper claimed that the precise control of the reaction temperature led to the regioselective N9 alkylation, we detected both N9 and N7 isomers in the reaction mixture. The gas chromatography analyses showed a relative ratio for N9 and N7 isomers from 89% to 11%. The required 2,6-dichloro-9-isopropyl-9*H*-purine was separated from the mixture of both isomers using crystallisation from ethyl acetate/hexane with a 60% yield.

Subsequently, the previously prepared adamantylated aromatic amines [41,42], or benzylamine, were introduced to position 6 of the purine **2a**. The nucleophilic aromatic substitution of chlorine at C6 led to the formation of compounds **3a**–**k** (Scheme 2). These reactions were carried out in the mixture of dimethyl formamide, the corresponding amine and triethylamine, from 80–100 °C [43]. Purines **3a**–**k** were obtained after purifying the crude material using column chromatography with good purity and yields of approximately 41–87%.

The final substitution of chlorine at C2 of compounds **3a**–**k** with 3-aminopropan-1-ol was relatively difficult. Initially, several reaction conditions, such as the utilisation of 4-(dimethylamino)pyridine [44] and 1-methylpyrrolidin-2-one [45], or the application of a stronger base (Hünig’s base) in DMSO, [46] were unsuccessful. Under these conditions, the desired product in the reaction mixture was either in a minor portion or not detected at all. Compounds **4a**–**k** were finally prepared under solvent-free conditions [47] with a significant excess of 3-aminopropan-1-ol at 160 °C in 3–6 h (Scheme 2).

All prepared compounds were fully characterised using spectral methods such as infrared (IR) and mass spectrometry (MS) and ^1^H- and ^13^C-nuclear magnetic resonance (see Experimental Section 3.3, Section 3.4, Section 3.5). Moreover, the structures of purines **2a**, **2b**, **3f**, **3j** and **3k** were confirmed using X-ray diffraction analyses [48,49,50]. NMR (^1^H and ^13^C), as well as the electrospray ionisation (ESI) mass spectra of compounds **4a**–**k**, are given in Supplementary Materials (Figures S1–S30).

### 2.2. Inclusion Complexes with β-CD

#### 2.2.1. ESI-MS Analyses

The ESI-MS was applied, not only for the study of host–guest complexes, but also to confirm the structure of the prepared compounds.

In the positive-ion first-order mass spectra of single purines, the dominant signal at *m*/*z* corresponding to the protonated molecule [M + H]^+^ was accompanied by the [M + Na]^+^ ion. It should be noted that the ion corresponding to the formation of dimers was not observed for any prepared purine in the gas phase. On the other hand, the existence of dimers, linked via H-bonds, was confirmed to be in a solid state for purines **3f**, **3j** and **3k** using X-ray diffraction analyses [49,50].

**Figure 2:**
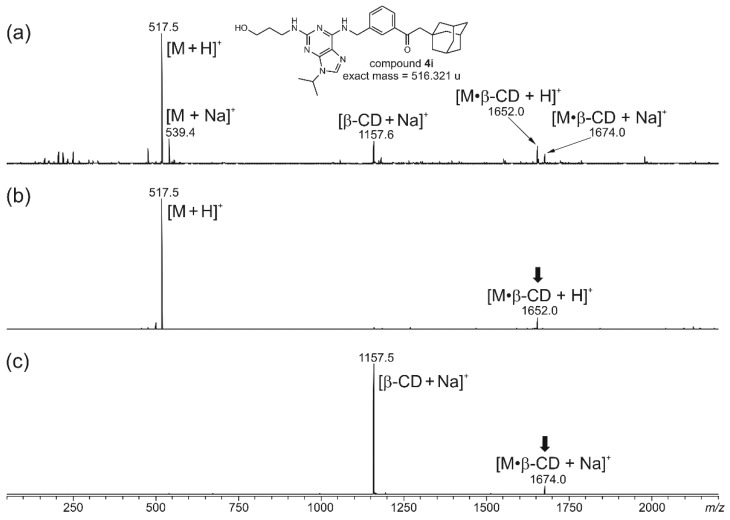
The positive-ion ESI mass spectra of the MeOH/H_2_O (1/1, *v*/*v*) solution of **4i**·β-CD: (**a**) first-order mass spectrum, (**b**) MS/MS of *m*/*z* 1652, (**c**) MS/MS of *m*/*z* 1674. The assignments for the observed ions are shown in the brackets. The fragmented ions in tandem mass spectra are marked with bold downward arrows.

The equimolar mixtures of individual purines with β-CD were prepared immediately before each experiment. In addition to the protonated molecule of the guest [M + H]^+^, its sodium adduct [M + Na]^+^ and the sodium adduct of β-CD [β-CD + Na]^+^, and two types of purine·β-CD aggregates (namely [M·β-CD + H]^+^ and [M·β-CD + Na]^+^) were detected for all purines bearing the adamantane moiety (Figure 2a). In the first-order mass spectra of purine **4k** (bohemine), only a signal of low intensity, which can be related to the protonated purine·β-CD aggregate, was detected. The existence of host–guest aggregates in the gas phase was confirmed using MS/MS experiments of [M·β-CD + H]^+^ and [M·β-CD + Na]^+^ ions under collision-induced dissociation (CID) conditions. The loss of neutral β-CD from the complex, and the formation of a singly charged product ion at *m*/*z* corresponding to the protonated guest molecule, represent a typical fragmentation pathway of protonated purine·β-CD aggregates (Figure 2b). In contrast, the fragmentation of the [M·β-CD + Na]^+^ ion led exclusively to the sodium adduct of β-CD (*m*/*z* 1157) in all cases (Figure 2c). This means that the neutral molecule of the guest was lost by this type of complex. The ESI mass spectra of host–guest complexes for all prepared purines **4a**–**k** are given in the Supplementary Materials (Figures S31–S40).

#### 2.2.2. ^1^H-^1^H Rotating Frame Nuclear Overhauser Effect Spectroscopy (ROESY) NMR Experiments

**Figure 3:**
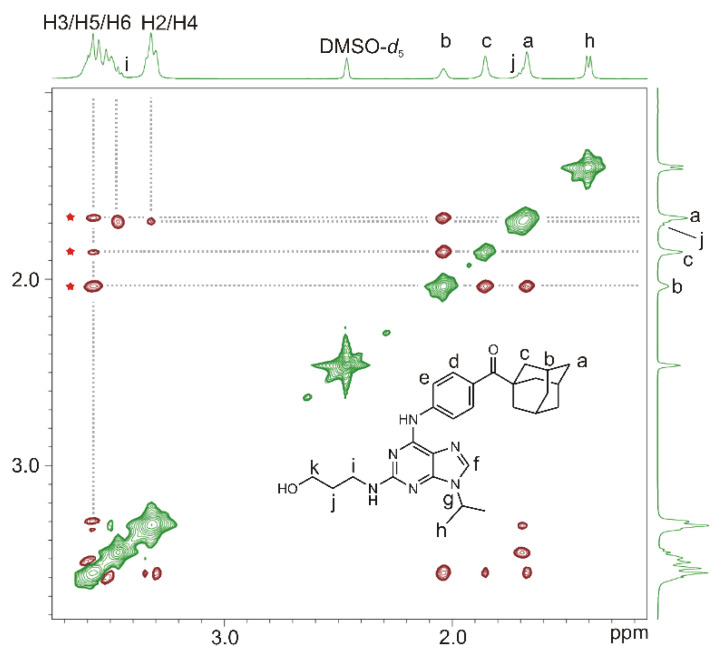
Portion of ROESY spectrum recorded for the 11.4 mM solution, consisting of purine **4d** and β-CD with a 1:1 molar ratio (D_2_O:DMSO-*d*_6_, 1:2, *v*:*v*; 303 K). The cross-peaks related to the intermolecular contacts inside the β-CD cavity are indicated by red stars. H2–H6 are H-atoms with glucose units of β-CD.

Our attempts to demonstrate the supramolecular properties of our purines were compromised by the low solubility of purines in water. Thus, isothermal calorimetric titrations and NMR titration experiments were disabled. However, we succeeded in 2D ROESY experiments to show, unambiguously, the supramolecular nature of purine complexes with β-CD. Figure 3 clearly shows cross-peaks, indicating that hydrogen atoms from the adamantane cage, that is, H(a), H(b) and H(c), interact with H5 and H3 atoms, which are located on the inside of the CD cylinder. This data concerts with the typical arrangement of inclusion complexes of adamantane derivatives and β-CD, where the adamantane cage is positioned inside the cavity of the CD. Combining the NMR and MS data, we can infer that our purines are able to form relatively stable inclusion complexes in water solutions.

### 2.3. CDK Inhibition Activity

The inhibitory potency of purines **4a**–**k** was tested with the recombinant heterodimeric complex CDK2/cyclin E. As can be seen in Table 1, the CDK inhibition activity was studied with both the single purines and their mixtures with β-CD (purine·β-CD, 1:1 and 1:10, molar ratio). Purine **4k** (bohemine) was used as a reference compound. Moreover, the activity of single compounds **4a**–**k** was compared with the previously published IC_50_ value of roscovitine [19]. The strongest activity has been observed for purines **4b** (IC_50_ = 0.21 βM) and **4d** (IC_50_ = 0.58 βM). The activity of purine **4b** on CDK2/cyclin E is 3.5-fold higher than bohemine and is essentially the same as that of roscovitine (Table 1). Compounds **4a**, **4e**, **4g** and **4j** inhibit CDK2/cyclin E in the micromolar range (IC_50_ = 0.92–3.26 βM). On the other hand, purines **4c** and **4i** displayed relatively weak inhibitory activity against CDK2/cyclin E in concentrations higher than 10 μM. The results mentioned above show that the presence of polar oxo, or the hydroxy function group, in the substituent at C6 of the purine ring positively influences the inhibitory activity of tested compounds. This point is further supported by the results of the inhibitory activity obtained for purines **4c**, **4f** and **4h** with a non-polar linker between the adamantane scaffold and the benzene ring. These compounds displayed weak or no inhibition activity towards CDK2/cyclin E. In the case of compounds with adamantylated benzylamine in position 6 (**4i** and **4j**), the *para* substituted derivative, **4j**, displayed 4.3-fold higher activity than its *meta*-analogue, **4i**. Of note, two purines bearing the adamantane moiety, linked directly by the N-atom at position 6 (**4m** and **4n** in Figure 1), were previously tested for their CDK inhibition activity with no positive results [18,20]. The presence of a bulky adamantane cage close to the purine ring was assumed to be the reason for the low activity.

The CDK inhibition activity was also studied for β-CD·purines **4a**–**k** complexes (1:1 and 10:1, molar ratio). The activity of the equimolar mixtures of β-CD with purines **4b**, **4e**, **4g** and **4k** was essentially consistent with their non-complexed forms. In the case of compounds **4a** and **4d**, the complexation with β-CD (1:1) resulted in an approximately 1.5–2.5 times decrease of their inhibitory activity. Purines **4f** and **4h** had no activity on CDK2/E, neither as single compounds nor as complexes with β-CD (both 1:1 and 1:10). The preparation of complexes purine·β-CD (1:10) led, with the exception of compounds **4b** and **4k**, to the dramatic decrease or total loss of inhibition activity. These results can be explained by the competition of β-CD and CDK for the purine ligand.

### 2.4. In Vitro Cytotoxicity

The antiproliferative activity of purines **4a**–**k** was assayed on two types of human tumour cell lines (K-562, chronic myelogenous leukaemia and MCF-7, breast adenocarcinoma). Analogous to that of CDK inhibition activity, the cytotoxicity was tested on single purines **4a**–**k**, as well as their mixtures with β-CD (Table 2). Purine **4d** was 17.8- and 9.2-fold more potent at inhibiting cell proliferation on K-562 than bohemine and roscovitine, respectively. In the case of MCF-7, the efficacy of **4d** was 4.7- and 2.2-fold higher to that of bohemine and roscovitine, respectively. Additionally, compounds **4a**, **4e** and **4f** showed lower growth inhibitory values (GI_50_) towards leukaemia and/or breast cancer cells in comparison with bohemine and roscovitine. Unfortunately, the study of the antiproliferative activity of single purines **4a**–**j** was compromised by the low solubility of the prepared purines. However, the solubility of compounds **4a**–**j** rapidly increased when they were mixed with β-CD. The cytotoxic effects of the equimolar mixtures of β-CD with adamantane-bearing purines, **4a**–**j**, on leukaemia and breast cancer cells were, in most cases, significantly higher to that of the β-CD·bohemine (**4k**) complex. Antiproliferative activity of 1:1 complexes of β-CD with purines **4a**, **4d**, **4e** and **4f** was 2–3 times lower than their non-complexed form. In the case of purine·β-CD (1:10) complexes, no cytotoxic effects were observed for all of the prepared compounds (except to purine **4k** towards MCF-7).

### 2.5. Docking Study

The docking study involved two CDK2 complexes (whose structures are available in the Protein Data Bank), the CDK2 (2A4L) and CDK2/cyclin E complex (5L2W), and ten purine derivatives, **4a**–**j**. For chiral compounds **4b**, **4e** and **4g**, both enantiomers were docked separately. Initially, we validated the reliability of the docking protocol by redocking the optimised dinaciclib structure in the CDK2/cyclin E active site. The superimposed experimental [52] and docked complex is shown in Figure S41 to demonstrate the essentially equal positioning of the two structures inside the binding site.

**Figure 4:**
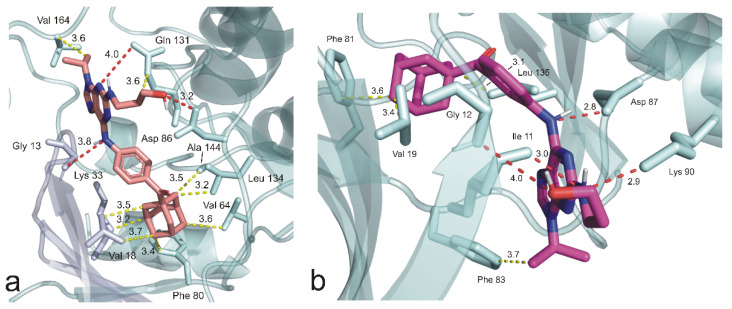
Purine **4d** (**a**) and purine **4a** (**b**) docked into the CDK2 and CDK2/cyclin E active site, respectively. Intermolecular contacts were analysed using PLIP. Non-polar contacts and H-bonds are shown as yellow and red dashed lines, respectively. Lengths are given in Å.

Considering the lowest energy geometries for each protein–ligand pair, three distinct orientations of the ligand inside the ATP binding pocket were detected in the case of the CDK2/cyclin E complex (5L2W). The most frequent typical orientation (i.e., the adamantane cage deep inside the binding pocket) can be seen in Figure 4b for the ligand **4a**. In this orientation, the adamantane cage contributes to complex stabilisation through non-polar interactions with Val 19, Phe 81 and Leu 135. The *N*9-isopropyl group is located near the portal of the binding site and displays non-polar interactions with Phe 83. The purine core is linked with a number of H-bond interactions to residues Ile 11, Leu 84, His 85, Asp 87 and Lys 90. In the case of three compounds, a flipped orientation of the ligand molecule (i.e., with the adamantane cage near the portal of the binding pocket) displayed minimum energy (for an example, see **4f**@CDK2/E in Figure S42). This orientation is stabilised through non-polar interactions with Val 65, Phe 81, Phe 83, Gln 132 and Leu 135, and H-bonds with Gln 132, Leu 84 and Asp 87.

In the binding pocket of CDK2 (2A4L), the most frequent arrangement of the ligand is stabilised via non-polar interactions between the adamantane cage and Val 18, Lys 33, Val 64, Phe 80, Leu 134 and Ala 144 (for the orientation of **4d**, see Figure 4a). In addition, N9-isopropyl has non-polar interactions with Val 164 and Glu 162, and the aminopurine ring interacts through the H-bonds with Gly 13 and Gln 131. Conformation of the *C*2-sidearm was stabilised via the H-bond between the OH group and Asp 86. The other orientations with minimum energy are shown in Figure S43 for **4a** and Figure S44 for **4j**.

A complete list of the intermolecular contacts recognised by PLIP software, and energies of the most stable arrangements of the ligand with CDK2 and CDK2/cyclin E (according to PyRx), are given in Tables S1 and S2, respectively.

Our docking study implicates three interesting conclusions. First, the binding pocket of the CDK2 is large enough to accommodate not only the bulky adamantane moiety but all of the molecules of our ligands. Therefore, the appropriate modification of the purine-based ligands (i.e., with a sufficiently long spacer at position *C*6) with an adamantane scaffold, which is motivated by the improvement of the pharmacologic and/or pharmacokinetic properties, does not disable the ligand binding to the active site. Second, in both examined protein structures, there are two distinct domains in which the adamantane cage can be accommodated. In the case of CDK2, the ligand orientation depends on the length of the spacer between the adamantane cage and purine ring. Ligands with longer spacers (**4g**–**4i**) prefer a positioning of the adamantane cage in the Val 164 domain, whereas ligands with shorter spacers (**4a**–**4f**) prefer an orientation with the adamantane cage in the Phe 80 domain. In the case of CDK2/cyclin E, the positioning of the adamantane cage in the Phe 81 domain predominates (9:4), with no clear correlation to the ligand geometry. Finally, we docked the previously published 6-[(1-adamantyl)amino]-2-[(3-hydroxypropyl)amino]-9-(propan-2-yl)purine (**4m**) [18] into the binding site of CDK2. This compound, **4m**, displayed no inhibition activity (IC_50_ > 0.1 mmol dm^−3^) towards CDK2 and CDK1. We compared the most stable arrangement of the inactive **4m** with those of our ligands, **4a**–**4f**, to clarify the role of the adamantane moiety. The superposed image is shown in Figure S45. As can be seen, the positioning of the compound **4m** inside the CDK2 binding site is driven by interactions of the adamantane moiety in the Phe 80 domain. Since the adamantane cage occupies essentially the same position in the case of guests **4a**–**4f** and **4m**, the purine ring of compound **4m** does not interact efficiently via H-bonds with residues within the pocket. This leads to significantly lower binding strength, as demonstrated in Table S1.

## 3. Materials and Methods

### 3.1. General Data

All starting compounds, reagents and solvents were purchased from commercial sources in analytical quality and were used without further purification. Adamantylated aromatic amines were prepared following previously published procedures [41,42,53]. Melting points were measured on a Kofler block and were not corrected. Elemental analyses (C, H, N, S) were performed with the Flash EA 1112 (Thermo Fisher Scientific, Waltham, MA, USA). Retention times were determined using thin layer chromatography (TLC) plates (Alugram Sil G/UV) from Macherey-Nagel (Germany). Two types of mobile phases were used: petroleum ether/ethyl acetate, 1/1, *v*/*v* (system a) and chloroform/methanol, 8/1, *v*/*v* (system b). The NMR spectra were recorded on a Bruker Avance-300 spectrometer at 300.13 MHz (^1^H) and 75.77 MHz (^13^C) or on an ECZ400R spectrometer (JEOL, Japan) at 399.78 MHz (^1^H) and 100.95 MHz (^13^C). ^1^H- and ^13^C-NMR chemical shifts were referenced to the signal of the solvent (^1^H: *δ*(residual CHCl_3_) = 7.27 ppm and *δ*(residual DMSO-*d*_5_) = 2.5 ppm; ^13^C: *δ*(CDCl_3_) = 77.23 ppm and *δ*(DMSO-*d*_6_) = 39.51 ppm). The ROESY experiments were carried out with a spin-lock time of 600 ms. The IR spectra were recorded in a KBr disc with a Nicolet Avatar-380 spectrophotometer. GC–EI–MS analyses were run on a QP-2010 instrument (Shimadzu, Japan) using a SLB-5ms (30 m, 0.25 mm; Supelco, Bellefonte, PA, USA) column. Helium was employed as a carrier gas in a constant linear flow mode (38 cm s^−1^): 100 °C/7 min, 25 °C/min to 250 °C, and held for the required amount of time. The DI–EI–MS spectra were performed on a QP-2010 instrument (Shimadzu, Japan) via the direct impact technique. Samples were prepared by drying 10 μL of the solution (0.03 mg mL^−1^) in dichloromethane in the DI cuvette at 50 °C. The DI method is 80 °C/1 min, 40 °C/min to 350 °C, and holding for the required amount of time, IS 200 °C/70 eV. Only peaks of the relative abundance exceeding 5% are listed. The electrospray mass spectra were recorded on an Esquire LC ion-trap mass spectrometer (Bruker Daltonics, Bremen, Germany) equipped with an ESI source. Sample solutions (8.8 μM in methanol/water, 1/1, *v*/*v*) were infused into the ion source using a syringe pump at a constant flow rate of 3 μL min^−1^. The other instrumental conditions were as follows: a capillary voltage of −3.5 kV, a drying gas temperature of 250 °C, a drying gas flow of 5 dm^3^ min^−1^ and a nebuliser pressure of 96.53 kPa. Nitrogen was used as both nebulising gas and drying gas. The nozzle–skimmer potential and octopole potential were modified and optimised before each experiment. Tandem mass spectra were carried out using CID with He as the collision gas after isolation of the required ions.

### 3.2. Molecular Docking Study

Molecules of the purines were built up in Avogadro v1.0.3 and optimised using a MMFF94s force field [54]. Previously reported crystal structures of CDK2 (2A4L) [55] and CDK2/cyclin E (5L2W) [52] were used as target proteins. Water molecules and ligands from the active site were removed in Avogadro. Molecular docking was performed using AutoDock Vina [56] implemented in PyRx v0.8 software [57]. The intermolecular contacts between the ligand and protein were analysed using an online tool, PLIP v2.2.0 [58].

### 3.3. Alkylation of 2,6-Dichloro-9H-purine (1)

The title compound was prepared following a modified literature procedure [40]. Potassium carbonate (9.9 g, 71.4 mmol) and 2-iodopropane (11.9 cm^3^, 119.0 mmol) were added to a well-stirred solution of 2,6-dichloro-9*H*-purine (4.5 g, 23.8 mmol) in DMSO (50 cm^3^). The reaction mixture was stirred at 15–18 °C for 8 h. After that, the mixture was diluted with water (50 cm^3^) and extracted with ether (7 × 15 cm^3^). Collected organic layers were washed with brine (2 × 10 cm^3^), dried over sodium sulphate and evaporated in vacuo. Both N9 and N7 isomers were separated after the crystallisation of the crude material from the ethyl acetate/hexane (1/1, *v*/*v*).

2,6-Dichloro-9-isopropyl-9*H*-purine (**2a**)

Colourless needles, yield 3.30 g (60%), mp 147–150 °C, R_f_ = 0.36 (system a). ^1^H NMR (CDCl_3_): *δ* 1.65 (d, *J* = 6.9 Hz, 6H, CH(C**H**_3_)_2_), 4.91 (septet, *J* = 6.9 Hz, 1H, C**H**(CH_3_)_2_), 8.17 (s, 1H, NC^8^HN) ppm. ^13^C NMR (CDCl_3_): *δ* 22.7(CH_3_), 48.6(CH), 131.3(C), 143.7(CH), 152.0(C), 152.9(C), 153.0(C) ppm. IR (KBr): 3119(w), 2988(w), 1783(w), 1588(s), 1556(s), 1487(m), 1463(m), 1359(s), 1318(m), 1275(m), 1245(s), 1217(s), 1187(m), 1158(s), 1139(m), 1108(w), 959(m), 878(s), 778(m), 682(w), 645(m), 628(m), 596(m) cm^−1^. GC-EI-MS *m*/*z* (%): 233(7), 232(22), 231(14), 230(M^+^, 34), 217(6), 215(9), 197(7), 195(20), 192(11), 191(7), 190(64), 189(12), 188(100), 161(6), 155(12), 153(39), 92(12), 64(6), 43(31), 42(8), 41(39). Anal. Calcd for C_8_H_8_Cl_2_N: C 41,58; H 3.49; N 24.25. Found: C 41.83; H 3.35; N 23.95.

2,6-Dichloro-7-isopropyl-7*H*-purine (**2b**)

Pale yellow crystalline powder, mp 152–154 °C, R_f_ = 0.28 (system a). ^1^H NMR (CDCl_3_): *δ* 1.68 (d, *J* = 6.7 Hz, 6H, CH(C**H**_3_)_2_), 5.23 (septet, *J* = 6.7 Hz, 1H, C**H**(CH_3_)_2_), 8.40 (s, 1H, NC^8^HN) ppm. ^13^C NMR (CDCl_3_): *δ* 23.7(CH_3_), 50.3(CH), 121.6(C), 143.8(CH), 147.2(C), 153.2(C), 163.8(C) ppm. IR (KBr): 3111(w), 2985(w), 1824(w), 1597(s), 1529(s), 1462(s), 1403(s), 1315(s), 1277(m), 1198(m), 1167(s), 1140(m), 1103(m), 997(s), 916(m), 869(s), 783(s), 683(w), 663(m), 634(s), 590(w) cm^−1^. GC-EI-MS *m*/*z* (%): 234(8), 233(5), 232(42), 231(7), 230(M^+^, 67), 217(36), 216(6), 215(59), 192(6), 191(12), 190(31), 189(20), 188(49), 181(5), 179(16), 155(24), 154(7), 153(73), 134(6), 127(6), 126(8), 118 (7), 101(5), 100(6), 99(7), 92(8), 91(7), 86(5), 85(6), 65(6), 64(8), 53(7), 43(100), 42(15), 41(73), 40(6).

### 3.4. General Procedure for Preparation of 6-“Amino”-2-chloro-9-isopropyl-9H-purines (**3a**–**k**)

Compounds **3a**–**k** were prepared following a slightly modified literature procedure [43]. Purine **2a** (100 mg, 0.43 mmol) was dissolved in DMF (2 cm^3^), and then corresponding amine (1.05 equivalent) and triethylamine (1.1 equivalent for a free base and 2 equivalents for hydrochloride) were added. The reaction mixture was stirred at 90 °C till the TLC indicated the consumption of all starting material (2–53 h). Subsequently, the mixture was diluted with water (a formation of soft precipitation was observed) and extracted with ether (4 × 10 cm^3^). Combined organic layers were washed twice with brine and dried over sodium sulphate. The solvent was removed by the evaporation in vacuo. Purification of the crude product using column chromatography (silicagel, petroleum ether/ethyl acetate, 1/1, *v*/*v*) resulted in the desired product.

(1-Adamantyl){3-[(2-chloro-9-isopropyl-9*H-*purin-6-yl)amino]phenyl}methanone (**3a**)

Prepared from **2a** and (1-adamantyl)(3-aminophenyl)methanone. Colourless needles, yield 115 mg (60%), mp 85–93 °C, R_f_ = 0.21 (system a). ^1^H NMR (CDCl_3_): *δ* 1.62 (d, *J* = 6.9 Hz, 6H, CH(C**H**_3_)_2_), 1.78 (m, 6H, CH_2_(Ad)), 2.09 (m, 9H, CH_2_(Ad) + CH(Ad)), 4.88 (septet, *J* = 6.6 Hz, 1H, C**H**(CH_3_)_2_), 7.29 (d, *J* = 7.6 Hz, 1H, Ph), 7.38 (t, *J* = 7.9 Hz, 1H, Ph), 7.69 (d, *J* = 8.3 Hz, 1H, Ph), 7.89 (s, 1H, Ph), 8.06 (s, 1H, NC^8^HN), 8.31 (s, 1H, C^6^N**H**Ph) ppm. ^13^C NMR (CDCl_3_): *δ* 23.0(CH_3_), 28.4(CH), 36.8(CH_2_), 39.3(CH_2_), 47.3(C), 47.6(CH), 119.3(CH), 121.9(CH), 123.3(CH), 129.1(CH), 137.7(C), 138.9(CH), 140.3(C), 140.4(C), 150.7(C), 152.4(C), 154.0(C), 209.8(CO) ppm. IR (KBr): 3324(w), 2904(s), 2850(m), 1667(m), 1622(s), 1571(s), 1451(m), 1318(m), 1226(m), 1027(w), 998(w), 943(w), 788(w), 735(w), 665(w) cm^−1^. DI-EI-MS *m*/*z* (%): 451(10), 450(9), 449(M^+^, 27), 314(12), 243(7), 136(11), 135(100), 107(9), 93(18), 91(5), 79(17), 67(7). Anal. Calcd for C_25_H_28_ClN_5_O: C 66.73; H 6.27; N 15.56. Found: C 66.83; H 6.38; N 15.44.

(1-Adamantyl){3-[(2-chloro-9-isopropyl-9*H*-purin-6-yl)amino]phenyl}methanol (**3b**)

Prepared from **2a** and (1-adamantyl)(3-aminophenyl)methanol. Colourless crystalline powder, yield 129 mg (67%), mp 198–201 °C, R_f_ = 0.28 (system a). ^1^H NMR (CDCl_3_): *δ* 1.54–1.73 (m, 18H, CH(C**H**_3_)_2_ + CH_2_(Ad)), 1.98 (m, 3H, CH(Ad)), 3.09 (s, 1H, CHO**H**), 4.24 (s, 1H, C**H**OH), 4.85 (septet, *J* = 6.9 Hz, 1H, C**H**(CH_3_)_2_), 7.01 (d, *J* = 7.6 Hz, 1H, Ph), 7.31 (d, *J* = 7.6 Hz, 1H, Ph), 7.59 (s, 1H, Ph), 7.72 (d, *J* = 7.8 Hz, 1H, Ph), 7.85 (s, 1H, NC^8^HN), 8.11 (s, 1H, C^6^N**H**Ph) ppm. ^13^C NMR (CDCl_3_): *δ* 23.0(CH_3_), 28.6(CH), 37.3(CH_2_), 37.6(C), 38.5(CH_2_), 47.5(CH), 83.0(CH), 119.6(CH), 120.5(CH), 124.0(CH), 128.2(CH), 137.5(C), 138.4(CH), 142.4(C), 150.6(C), 152.5(C), 152.6(C), 154.1(C) ppm. IR (KBr): 3322(w), 2902(s), 2848(m), 1644(s), 1578(s), 1470(m), 1347(m), 1317(m), 1217(m), 1058(w), 1031(m), 944(w), 668(w) cm^−1^. DI-EI-MS *m*/*z* (%): 451(M^+^, 5), 319(5), 318(26), 316(77), 315(8), 276(5), 274(14), 238(7), 210(5), 136(11), 135(100), 107(12), 93(23), 91(5), 79(21), 77(8), 67(9). Anal. Calcd for C_25_H_30_ClN_5_O: C 66.43; H 6.69; N 15.49. Found: C 66.38; H 6.79 N 15.21.

N-[3-(1-Adamantylmethyl)phenyl]-2-chloro-9-isopropyl-9*H*-purin-6-amine (**3c**)

Prepared from **2a** and 3-(1-adamantylmethyl)anilinium chloride. Colourless crystalline powder, yield 107 mg (57%), mp 63–68 °C, R_f_ = 0.45 (system a). ^1^H NMR (CDCl_3_): *δ* 1.54–1.67 (m, 18H, CH(C**H**_3_)_2_ + CH_2_(Ad)), 1.96 (m, 3H, CH(Ad)), 2.41 (s, 2H, PhC**H**_2_Ad), 4.86 (septet, *J* = 7.6 Hz, 1H, C**H**(CH_3_)_2_), 6.88 (d, *J* = 7.6 Hz, 1H, Ph), 7.28 (t, *J* = 7.6 Hz, 1H, Ph), 7.49 (s, 1H, Ph), 7.67 (d, *J* = 7.9 Hz, 1H, Ph), 8.03 (s, 1H, NC^8^HN), 8.30 (s, 1H, C^6^N**H**Ph) ppm. ^13^C NMR (CDCl_3_): *δ* 22.9(CH_3_), 29.0(CH), 33.8(C), 37.2(CH_2_), 42.7(CH_2_), 47.8(CH), 51.5(CH_2_), 118.2(CH), 123.2(CH), 126.9(CH), 128.3(CH), 136.1(C), 137.3(C), 138.1(C), 139.5(CH), 150.3(C), 152.3(C), 154.6(C) ppm. IR (KBr): 3289(w), 3195(w), 3126(w), 2976(w), 2903(s), 2845(m), 1623(s), 1597(s), 1572(s), 1452(bm), 1345(m), 1316(s), 1224(m), 1199(w), 1161(w), 1029(m), 942(w), 883(w), 788(w), 717(w), 699(w), 666(w), 639(w) cm^−1^. DI-EI-MS *m*/*z* (%): 437(9), 436(9), 435(M^+^, 25), 136(11), 135(100), 107(9), 93(16), 81(5), 79(16), 67(7). Anal. Calcd for C_25_H_30_ClN_5_: C 68.87; H 6.94; N 16.06. Found: C 69.17; H 6.98; N 15.80.

(1-Adamantyl){4-[(2-chloro-9-isopropyl-9*H*-purin-6-yl)amino]phenyl}methanone (**3d**)

Prepared from **2a** and (1-adamantyl)(4-aminophenyl)methanone. Pale orange crystalline powder, yield 91 mg (47%), mp 67–72 °C, R_f_ = 0.20 (system a). ^1^H NMR (CDCl_3_): *δ* 1.62 (d, *J* = 6.6 Hz, 6H, CH(C**H**_3_)_2_), 1.78 (m, 6H, CH_2_(Ad)), 2.08 (m, 9H, CH_2_(Ad) + CH(Ad)), 4.88 (septet, *J* = 6.9 Hz, 1H, C**H**(CH_3_)_2_), 7.78 (d, *J* = 8.6 Hz, 2H, Ph), 7.87 (d, *J* = 8.6 Hz, 2H, Ph), 7.91 (s, 1H, NC^8^HN), 8.31 (s, 1H, C^6^N**H**Ph) ppm. ^13^C NMR (CDCl_3_): *δ* 22.9(CH_3_), 28.5(CH), 36.9(CH_2_), 39.6(CH_2_), 47.2(C), 47.7(CH), 119.1(CH), 119.7(C), 129.6(CH), 134.1(C), 139.0(CH), 140.7(C), 150.8(C), 152.1(C), 154.0(C), 207.8(CO) ppm. IR (KBr): 3324(w), 2905(s), 2851(m), 1626(m), 1605(m), 1572(s), 1507(w), 1452(m), 1413(w), 1322(m), 1271(w), 1236(m), 1175(m), 1027(w), 987(w), 846(w), 638(w) cm^−1^. DI-EI-MS *m*/*z* (%): 451(7), 450(6), 449(M^+^, 19), 317(6), 316(33), 315(18), 314(100), 272(11), 244(7), 135(32), 107(7), 93(17), 91(6), 79(18), 77(6), 67(7), 55(5). Anal. Calcd for C_25_H_28_ClN_5_O: C 66.73; H 6.27; N 15.56. Found: C 67.05; H 6.59; N 15.80.

(1-Adamantyl){4-[(2-chloro-9-isopropyl-9*H*-purin-6-yl)amino]phenyl}methanol (**3e**)

Prepared from **2a** and (1-adamantyl)(4-aminophenyl)methanol. Pale orange crystalline powder, yield 92 mg (47%), mp 289–293 °C, R_f_ = 0.19 (system a). ^1^H NMR (CDCl_3_): *δ* 1.52–1.66 (m, 18H, CH(C**H**_3_)_2_ + CH_2_(Ad)), 1.99 (m, 3H, CH(Ad)), 2.10 (s, 1H, CHO**H**), 4.24 (s, 1H, C**H**OH), 4.88 (m, C**H**(CH_3_)_2_), 7.28–7.31 (d + s, 3H, Ph + solvent), 7.74 (m, 2H, Ph + NC^8^HN), 7.88 (s, 1H, C^6^N**H**Ph) ppm. ^13^C NMR (CDCl_3_): *δ* 23.0(CH_3_), 28.6(CH), 37.3(CH_2_), 38.5(CH_2_), 58.5(CH), 82.9(CH), 119.4(CH), 128.7(CH), 134.6(C), 138.2(CH), 137.7(C), 152.6(C), 160.0(C) ppm. IR (KBr): 3349(w), 2905(s), 2847(m), 1635(s), 1579(s), 1511(m), 1463(m), 1345(m), 1320(m), 1219(m), 1029(m), 942(w), 844(w), 787(w), 670(w) cm^−1^. DI-EI-MS *m*/*z* (%): 451(M^+^, 11), 435(5), 319(6), 318(33), 317(30), 316(100), 315(38), 314(6), 276(13), 275(7), 274(40), 246(5), 238(10), 210(12), 135(37), 134(5), 107(10), 93(19), 91(6), 81(6), 79(22), 77(12), 67(8). Anal. Calcd for C_25_H_30_ClN_5_O: C 66.43; H 6.69; N 15.49. Found: C 66.56; H 6.55; N 15.71.

N-[4-(1-Adamantylmethyl)phenyl]-2-chloro-9-isopropyl-9*H*-purin-6-amine (**3f**)

Prepared from **2a** and 4-(1-adamantylmethyl)anilinium chloride. Colourless crystalline powder, yield 77 mg (41%), mp 180–184 °C, R_f_ = 0.44 (system a). ^1^H NMR (CDCl_3_): *δ* 1.50–1.70 (m, 18H, CH(C**H**_3_)_2_ + CH_2_(Ad)), 1.94 (m, 3H, CH(Ad)), 2.37 (s, 2H, PhC**H**_2_Ad), 4.86 (septet, *J* = 6.9 Hz, 1H, C**H**(CH_3_)_2_), 7.11 (d, *J* = 7.9 Hz, 2H, Ph), 7.67 (d, *J* = 7.9 Hz, 2H, Ph), 7.85 (s, 1H, NC^8^HN), 7.89 (s, 1H, C^6^N**H**Ph) ppm. ^13^C NMR (CDCl_3_): *δ* 23.0(CH_3_), 29.0(CH), 33.8(C), 37.2(CH_2_), 42.6(CH_2_), 47.4(CH), 50.9(CH_2_), 119.6(CH), 119.8(C), 131.3(CH), 134.3(C), 136.1(C), 138.5(CH), 150.4(C), 152.6(C), 154.1(C) ppm. IR (KBr): 3268(w), 3183(w), 3131(w), 2976(w), 2906(s), 2843(m), 1631(s), 1579(s), 1511(s), 1462(s), 1417(w), 1348(s), 1314(s), 1223(m), 1194(m), 1027(m), 942(m), 848(m), 787(w), 759(w), 659(w), 639(w), 610(w) cm^−1^. DI-EI-MS *m*/*z* (%): 437(13), 436(11), 435(M^+^, 37), 302(7), 300(23), 257(11), 222(7), 136(11), 135(100), 107(9), 97(5), 93(18), 91(5), 85(6), 83(5), 81(7), 79(16), 71(8), 69(7), 67(9), 57(15), 55(11). Anal. Calcd for C_25_H_30_ClN_5_: C 68.87; H 6.94; N 16.06. Found: C 69.03; H 6.91; N 15.82.

2-(1-Adamantyl)-1-{3-[(2-chloro-9-isopropyl-9*H*-purin-6-yl)amino]phenyl}ethan-1-ol (**3g**)

Prepared from **2a** and 2-(1-adamantyl)-1-(3-aminophenyl)ethan-1-ol. Colourless crystalline powder, yield 98 mg (49%), mp 210–215 °C, R_f_ = 0.44 (system a). ^1^H NMR (CDCl_3_): *δ* 1.48–1.94 (m, 20H, CH(C**H**_3_)_2_ + CH_2_(Ad)+ PhCHC**H**_2_Ad), 2.49 (m, 3H, CH(Ad)), 2.51 (s, 1H, CHO**H**), 4.74 (m, 2H, C**H**OH + C**H**(CH_3_)_2_), 7.03 (d, *J* = 7.6 Hz, 1H, Ph), 7.20 (t, *J* = 7.6 Hz, 1H, Ph), 7.64 (d, *J* = 8.5 Hz, 1H, Ph), 7.75 (s, 1H, Ph), 8.03 (s, 1H, NC^8^HN), 8.63 (s, 1H, C^6^N**H**Ph) ppm. ^13^C NMR (CDCl_3_): *δ* 22.8(CH_3_), 28.8(CH), 32.7(C), 37.3(CH_2_), 43.2(CH_2_), 47.5(CH), 54.2(CH_2_), 70.8(CH), 118.4(CH), 119.1(C), 119.4(CH), 121.6(CH), 129.1(CH), 138.3(CH), 138.4(C), 147.8(C), 150.4(C), 152.4(C), 154.1(C) ppm. IR (KBr): 3319(w), 3241(w), 2899(s), 2846(m), 1642(m), 1573(s), 1500(w), 1467(m), 1424(w), 1347(m), 1324(m), 1289(w), 1222(m), 1200(w), 1037(w), 1001(w), 876(w), 794(m), 636(w) cm^−1^. DI-EI-MS *m*/*z* (%): 467(15), 466(13), 465(M^+^, 43), 463(7), 449(10), 448(8), 447(18), 319(6), 318(33), 317(21), 316(100), 315(8), 314(7), 288(11), 276(27), 275(13), 274(81), 272(6), 246(10), 244(7), 238(21), 210(18), 135(23), 107(13), 105(6), 93(27), 92(6), 91(14), 81(10), 79(27), 77(17), 67(16), 55(10), 43(10), 41(19). Anal. Calcd for C_26_H_32_ClN_5_O: C 67.01; H 6.92; N 15.03. Found: C 67.21; H 7.19; N 14.86.

N-{3-[2-(1-Adamantyl)ethyl]phenyl}-2-chloro-9-isopropyl-9*H*-purin-6-amine (**3h**)

Prepared from **2a** and 3-[2-(1-adamantyl)ethyl]anilinium chloride. Colourless crystalline powder, yield 87 mg (45%), mp 55–60 °C, R_f_ = 0.41 (system a). ^1^H NMR (CDCl_3_): *δ* 1.57–1.72 (m, 20H, CH(C**H**_3_)_2_ + CH_2_(Ad)+ PhCH_2_C**H**_2_Ad), 1.99 (m, 3H, CH(Ad)), 2.59 (t, *J* = 7.9 Hz, 2H, PhC**H**_2_CH_2_Ad), 4.87 (m, 1H, C**H**(CH_3_)_2_), 6.97 (d, *J* = 6.9 Hz, 1H, Ph), 7.30 (t, *J* = 6.6 Hz, 1H, Ph), 7.56 (s, 1H, Ph), 7.67 (d, *J* = 7.9 Hz, 1H, Ph), 7.95 (s, 1H, NC^8^HN), 8.19 (s, 1H, C^6^N**H**Ph) ppm. ^13^C NMR (CDCl_3_): *δ* 22.9(CH_3_), 29.0(CH), 29.3(CH_2_), 32.7(C), 37.5(CH_2_), 42.6(CH_2_), 46.8(CH_2_), 47.6(CH), 117.8(CH), 119.0(C), 120.6(CH), 124.4(CH), 129.1(CH), 136.1(C), 138.2(C), 138.3(CH), 145.1(C), 152.4(C), 154.3(C) ppm. IR (KBr): 3284(w), 3200(w), 3118(w), 2977(w), 2902(s), 2845(m), 1622(s), 1594(s), 1572(s), 1453(m), 1345(m), 1316(s), 1290(w), 1224(m), 1200(w), 1161(w), 1029(m), 941(w), 876(w), 788(w), 695(w), 665(w), 640(w) cm^−1^. DI-EI-MS *m*/*z* (%): 452(10), 451(37), 450(39), 449(M^+^, 100), 448(31), 316(17), 315(11), 314(52), 303(10), 302(6), 301(31), 274(7), 272(23), 261 (7), 260(7), 259(21), 258(14), 236(8), 224(5), 221(6), 136(7), 135(58), 107(19), 93(38), 91(15), 81(14), 79(39), 77(14), 67(22), 55(10), 43(6), 41(17). Anal. Caldc for C_26_H_32_ClN_5_: C 69.39; H 7.17; N 15.56. Found: C 69.49; H 7.11; N 15.67.

2-(1-Adamantyl)-1-{3-[({2-chloro-9-isopropyl-9*H*-purin-6-yl}amino)methyl]phenyl}ethan-1-one (**3i**)

Prepared from **2a** and 2-(1-adamantyl)-1-[3-(aminomethyl)phenyl]ethan-1-one. Colourless crystalline powder, yield 158 mg (77%), mp 165–170 °C, R_f_ = 0.33 (system a). ^1^H NMR (CDCl_3_): *δ* 1.55–1.61 (m, 18H, CH(C**H**_3_)_2_ + CH_2_(Ad)), 1.91 (m, 3H, CH(Ad)), 2.69 (s, 2H, PhCOC**H**_2_Ad), 4.78–4.91 (m + s, 3H, C**H**(CH_3_)_2_ + C^6^NHC**H**_2_Ph), 6.67 (bs, 1H, C^6^N**H**CH_2_Ph), 7.41 (m, 1H, Ph), 7.55 (m, 1H, Ph), 7.68 (s, 1H, Ph), 7.85 (d, *J* = 6.3 Hz, 1H, Ph), 7.98 (s, 1H, NC^8^HN) ppm. ^13^C NMR (CDCl_3_): *δ* 22.9(CH_3_), 28.9(CH), 34.2(C), 36.9(CH_2_), 43.2(CH_2_), 47.2(CH), 51.5(CH_2_), 119.1(C), 127.9(CH), 128.1(CH), 129.0(CH), 132.3(C), 138.0(CH), 138.9(CH), 139.5(C), 154.4(C), 155.3(C), 173.2(C), 200.1(CO) ppm. IR (KBr): 3267(w), 2902(s), 2847(m), 1666(m), 1631(s), 1578(m), 1541(w), 1473(m), 1353(m), 1312(s), 1296(m), 1263(m), 1234(s), 1203(m), 1077(w), 972(w), 756(w), 662(m), 639(w) cm^−1^. DI-EI-MS *m*/*z* (%): 480(10), 479(35), 478(32), 477(M^+^, 100), 443(10), 442(31), 344(24), 343(14), 342(66), 330(8), 328(23), 302(11), 301(14), 300(27), 260(6), 259(7), 258(17), 222(12), 214(11), 212(33), 182(10), 170(6), 143(8), 136(8), 135(69), 134(6), 133(9), 119(27), 118(12), 107(14), 105(9), 104(8), 93(34), 92(9), 91(30), 90(21), 89(12), 81(7), 79(34), 77(12), 67(15). Anal. Calcd for C_27_H_32_ClN_5_O: C 67.84; H 6.75; N 14.65. Found: C 68.02; H 6.54; N 14.51.

2-(1-Adamantyl)-1-{4-[({2-chloro-9-isopropyl-9*H*-purin-6-yl}amino)methyl]phenyl}ethan-1-one (**3j**)

Prepared from **2a** and 2-(1-adamantyl)-1-[4-(aminomethyl)phenyl]ethan-1-one. Colourless crystalline powder, yield 179 mg (87%), mp 146–150 °C, R_f_ = 0.31 (system a). ^1^H NMR (CDCl_3_): *δ* 1.55 (d, *J* = 6.3 Hz, 6H, CH(C**H**_3_)_2_), 1.64 (m, 12H, CH_2_(Ad)), 1.94 (m, 3H, CH(Ad)), 2.69 (s, 2H, PhCOC**H**_2_Ad), 4.76–4.91 (m + s, 3H, C**H**(CH_3_)_2_ + C^6^NHC**H**_2_Ph), 6.66 (bs, 1H, C^6^N**H**CH_2_Ph), 7.43 (d, *J* = 7.6 Hz, 2H, Ph), 7.67 (s, 1H, NC^8^HN), 7.90 (d, *J* = 7.6 Hz, 2H, Ph) ppm. ^13^C NMR (CDCl_3_): *δ* 22.9(CH_3_), 28.9(CH), 34.2(C), 37.0(CH_2_), 43.2(CH_2_), 47.2(CH), 51.5(CH_2_), 119.1(C), 128.0(CH), 129.1(CH), 138.0(CH), 138.4(C), 143.4(C), 154.4(C), 155.3(C), 199.9(CO) ppm. IR (KBr): 3261(w), 2902(s), 2846(m), 1667(m), 1623(s), 1573(m), 1537(w), 1466(m), 1406(m), 1349(m), 1314(s), 1292(m), 1261(m), 1226(s), 1204(m), 1077(w), 1013(w), 977(w), 923(w), 788(w), 664(w) cm^−1^. DI-EI-MS *m*/*z* (%): 480(10), 479(35), 478(31), 477(M^+^, 100), 463(11), 442(7), 344(9), 343(6), 342(25), 330(13), 329(7), 328(38), 302(5), 300(14), 286(10), 282(14), 266(20), 260(7), 259(6), 258(21), 257(6), 225(7), 222(8), 214(18), 213(6), 212(55), 170(11), 136(9), 135(74), 133(10), 119(16), 118(16), 107(14), 105(12), 104(7), 93(33), 92(8), 91(23), 90(21), 89(17), 81(9), 79(32), 77(12), 67(16). Anal. Calcd for C_27_H_32_ClN_5_O: C 67.84; H 6.75; N 14.65. Found: C 68.06; H 6.79; N 14.78.

N-Benzyl-2-chloro-9-isopropyl-9*H*-purin-6-amine (**3k**)

Prepared from **2a** and benzylaminium chloride. Colourless crystalline powder, yield 90 mg (69%), mp 175–177 °C, R_f_ = 0.34 (system a). ^1^H NMR (CDCl_3_): *δ* 1.56 (d, *J* = 6.3 Hz, 6H, CH(C**H**_3_)_2_), 4.85 (m + s, 3H, C**H**(CH_3_)_2_ + C^6^NHC**H**_2_Ph), 6.85 (bs, 1H, C^6^N**H**CH_2_Ph), 7.37 (m, 5H, Ph), 7.53 (s, 1H, NC^8^HN) ppm. ^13^C NMR (CDCl_3_): *δ* 22.9(CH_3_), 47.1(CH), 119,0(C), 127,8(CH), 128,1(CH), 128,9(CH), 137,9(CH), 138,4(C), 139,6(C), 155,5(C), 155,4(C) ppm. IR (KBr): 3266(w), 3125(w), 2979(w), 1715(w), 1626(s), 1571(m), 1536(w), 1453(w), 1354(m), 1311(m), 1292(m), 1254(w), 1229(m), 1202(w), 1068(w), 930(w), 724(w), 695(w), 660(w) cm^−1^. GC-EI-MS *m*/*z* (%): 303(18), 302(11), 301(M^+^, 54), 261(6), 260(14), 259(19), 258(36), 224(6), 223(5), 222(7), 161(5), 154(9), 153(5), 119(20), 107(9), 106(100), 92(11), 91(70), 89(6), 79(7), 77(6), 65(22). Anal. Calcd for C_15_H_16_ClN_5_: C 59.70; H 5.34; N 23.21. Found: C 59.68; H 5.45; N 22.88.

### 3.5. General Procedure for Preparation of 2,6-“Diamino”-9-isopropyl-9H-purines (**4a**–**k**)

Compounds **4a**–**k** were prepared using a slightly modified literature procedure [47]. A mixture of corresponding purine **3a**–**m** (0.15–0.25 mmol) and 3-aminopropan-1-ol (8 equivalents) was vigorously stirred at 160 °C in an argon atmosphere for 3–6 h. After the consumption of all starting purines (monitored by TLC), the reaction mixture was cooled to room temperature, diluted with chloroform (20 cm^3^) and washed several times with water (an unexpended excess of 3-aminopropan-1-ol was removed from the mixture). The organic layer was washed with brine (5 cm^3^), dried over sodium sulphate and evaporated in vacuo. The desired product was obtained after the purification of crude material using column chromatography (silicagel; chloroform/methanol, 8/1, *v*/*v*).

(1-Adamantyl)[3-({2-[(3-hydroxypropyl)amino]-9-isopropyl-9*H*-purin-6-yl}amino)phenyl]methanone (**4a**)

Prepared from **3a** (98 mg, 0.22 mmol) and 3-aminopropan-1-ol (132 mg, 1.76 mmol). Colourless crystalline powder, yield 47 mg (44%), mp 85–89 °C, R_f_ = 0.43 (system b). ^1^H NMR (CDCl_3_): *δ* 1.60 (d, *J* = 6.9 Hz, 6H, CH(C**H**_3_)_2_), 1.71–1.89 (m, 8H, CH_2_(Ad)+ NHCH_2_C**H**_2_CH_2_OH), 2.09 (m, 9H, CH_2_(Ad) + CH(Ad)), 3.68 (m, 4H, NHC**H**_2_CH_2_C**H**_2_OH), 4.69 (septet, *J* = 6.6 Hz, 1H, C**H**(CH_3_)_2_), 5.14 (t, 1H, *J* = 6.3 Hz, N**H**(CH_2_)_3_OH), 7.29–7.37 (m, 2H, Ph), 7.64 (s, 1H, Ph), 7.74 (d, *J* = 7.6 Hz, 1H, Ph), 7.83 (s, 1H, NC^8^HN), 8.18 (s, 1H, C^6^N**H**Ph) ppm. ^13^C NMR (CDCl_3_): *δ* 22.9(CH_3_), 28.4(CH), 33.7(CH_2_), 36.7(CH_2_), 38.0(CH_2_), 39.4(CH_2_), 46.7(C), 47.2(CH), 58.8(CH_2_), 119.4(CH), 121.9(CH), 122.1(CH), 128.7(CH), 135.5(C), 138.9(CH), 140.0(C), 152.6(C), 160.1(C), 209.6(CO) ppm. IR (KBr): 3331(m), 3246(m), 3119(w), 2904(s), 2849(m), 1627(s), 1578(s), 1523(m), 1482(m), 1410(w), 1323(w), 1250(m), 1214(w), 1058(m), 997(m), 884(w), 786(m), 641(w) cm^−1^. DI-EI-MS *m*/*z* (%): 489(33), 488(M^+^, 100), 487(10), 459(6), 458(20), 457(40), 445(15), 444(34), 443(33), 431(7), 430(18), 429(7), 416(10), 415(14), 402(6), 401(12), 282(7), 268(8), 235(5), 234(31), 226(6), 225(6), 136(6), 135(54), 107(15), 93(29), 91(8), 79(33), 77(7), 73(6), 67(6), 45(9), 44(7). ESI-MS (pos.) *m*/*z* (%): 489.5 [M + H]^+^ (100). Anal. Calcd for: C_28_H_36_N_6_O_2_: C 68.83; H 7.43; N 17.20. Found: C 68.98; H 7.26; N 17.41.

3-{[6-({3-[1-Adamantyl(hydroxy)methyl]phenyl}amino)-9-isopropyl-9*H*-purin-2-yl]amino}propan-1-ol (**4b**)

Prepared from **3b** (92 mg, 0.20 mmol) and 3-aminopropan-1-ol (120 mg, 1.60 mmol). Colourless crystalline powder, yield 92 mg (94%), mp 88–93 °C, R_f_ = 0.41 (system b). ^1^H NMR (CDCl_3_): *δ* 1.52–1.75 (m, 20H, CH(C**H**_3_)_2_ + CH_2_(Ad) + NHCH_2_C**H**_2_CH_2_OH), 1.93 (m, 3H, CH(Ad)), 2.65 (bs, 1H, CHO**H**), 3.63 (m, 4H, NHC**H**_2_CH_2_C**H**_2_OH), 4.20 (s, 1H, C**H**OH), 4.65 (septet, *J* = 6.6 Hz, 1H, C**H**(CH_3_)_2_), 5.34 (bs, 1H, NH(CH_2_)_3_O**H**), 6.92 (d, *J* = 7,3 Hz, 1H, Ph), 7.24 (m, 2H, Ph), 7.53–7.59 (m, 2H, Ph + NC^8^HN), 7.72–7.76 (m, 2H, C^6^N**H**Ph+ N**H**(CH_2_)_3_OH) ppm. ^13^C NMR (CDCl_3_): *δ* 22.8(CH_3_), 28.6(CH), 33.5(CH_2_), 37.3(CH_2_), 37.4(C), 38.1(CH_2_), 38.4(CH_2_), 46.5(CH), 58.7(CH_2_), 82.7(CH), 114.8(C), 119.3(CH), 120.2(CH), 123.3(CH), 127.7(CH), 134.9(C), 138.3(CH), 142.6(C), 150.9(C), 152.7(C), 160.2(C) ppm. IR (KBr): 3343(m), 3246(m), 3198(w), 2903(s), 2847(m), 1625(s), 1583(s), 1524(m), 1488(m), 1420(w), 1370(w), 1250(m), 1215(w), 1226(w), 1047(m), 983(w), 885(w), 788(m), 756(m), 732(w), 702(w), 640(w) cm^−1^. DI-EI-MS *m*/*z* (%): 491(19), 490(M^+^, 61), 441(8), 356(23), 355(100), 313(9), 311(12), 297(7), 267(11), 234(11), 135(Ad, 38), 107(9), 93(18), 79(20), 77(5). ESI-MS (pos.) *m*/*z* (%): 491.5 [M + H]^+^ (100). Anal. Calcd for C_28_H_38_N_6_O_2_: C 68.54; H 7.81; N 17.13. Found: C 68.39; H 7.93; N 17.27.

3-{[6-{[3-(1-Adamantylmethyl)phenyl]amino}-9-isopropyl-9*H*-purin-2-yl]amino}propan-1-ol (**4c**)

Prepared from **3c** (90 mg, 0.21 mmol) and 3-aminopropan-1-ol (126 mg, 1.68 mmol). Colourless crystalline powder, yield 70 mg (70%), mp 88–93 °C, R_f_ = 0.67 (system b). ^1^H NMR (CDCl_3_): *δ* 1.50–1.92 (m, 23H, CH(C**H**_3_)_2_ + CH_2_(Ad) + NHCH_2_C**H**_2_CH_2_OH+ CH(Ad)), 2.35 (s, 2H, PhC**H**_2_Ad), 3.65 (m, 4H, NHC**H**_2_CH_2_C**H**_2_OH), 4.65 (m, 2H, C**H**(CH_3_)_2_ + NH(CH_2_)_3_O**H**), 5.21 (bs, 2H, N**H**(CH_2_)_3_OH), 6.79 (d, *J* = 7.3 Hz, 1H, Ph), 7.20 (t, *J* = 7.6 Hz, 1H, Ph), 7.31 (s, 1H, Ph), 7.58 (s, 1H, NC^8^HN), 7.71 (d, *J* = 7.9 Hz, 1H, Ph), 7.78 (s, 1H, C^6^N**H**Ph) ppm. ^13^C NMR (CDCl_3_): *δ* 22.8(CH_3_), 28.9(CH), 33.4(CH_2_), 33.8(C), 37.2(CH_2_), 37.9(CH_2_), 42.7(CH_2_), 46.6(CH), 51.5(CH_2_), 58.7(CH_2_), 115.1(C), 118.0(CH), 122.7(CH), 125.9(CH), 128.1(CH), 135.0(C), 138.3(C), 139.2(CH), 150.8(C), 152.8(C), 160.2(C) ppm. IR (KBr): 3328(m), 3244(m), 3121(w), 2902(s), 2844(m), 1625(s), 1581(s), 1522(m), 1488(s), 1418(w), 1324(w), 1247(m), 1215(w), 1227(w), 1050(w), 884(w), 788(m), 755(m), 718(w), 700(w), 640(w) cm^−1^. DI-EI-MS *m*/*z* (%): 475(33), 474(M^+^, 100), 473(12), 444(18), 443(37), 431(15), 430(35), 429(31), 416(10), 415(8), 401(9), 387(7), 253(5), 251(5), 234(19), 135(Ad, 36), 93(20), 91(6), 79(23), 45(7). ESI-MS (pos.) *m*/*z* (%): 475.5 [M + H]^+^ (100). Anal. Calcd for C_28_H_38_N_6_O: C 70.85; H 8.07; N 17.71. Found: 70.73; H 7.12; N 17.80.

(1-Adamantyl)[4-({2-[(3-hydroxypropyl)amino]-9-isopropyl-9*H*-purin-6-yl}amino)phenyl]methanone (**4d**)

Prepared from **3d** (68 mg, 0.15 mmol) and 3-aminopropan-1-ol (90 mg, 1.20 mmol). Pale orange crystalline powder, yield 35 mg (48%), mp 88–93 °C, R_f_ = 0.67 (system b). ^1^H NMR (DMSO-*d_5_*): *δ* 1.50 (d, *J* = 5.1 Hz, 6H, CH(C**H**_3_)_2_), 1.74 (m, 8H, (CH_2_)Ad+ NHCH_2_C**H**_2_CH_2_OH), 1.99 (m, 6H, (CH_2_)Ad), 2.04 (m, 3H, CH(Ad)), 3.51 (m, 2H, NHC**H**_2_CH_2_C**H**_2_OH), 4.47 (m, 1H, NH(CH_2_)_3_O**H**), 4.60 (m, 1H, C**H**(CH_3_)_2_), 6.66 (m, 1H, N**H**(CH_2_)_3_OH), 7.72 (d, *J* = 6.6 Hz, 2H, Ph), 7.97 (s, 1H, NC^8^HN), 8.12 (d, *J* = 6.6 Hz, 2H, Ph), 9.68 (s, 1H, C^6^N**H**Ph) ppm. ^13^C NMR (DMSO-*d_6_*): *δ* 22.0(CH_3_), 27.7(CH), 32.5(CH_2_), 36.1(CH_2_), 45.8(C), 46.0(CH), 58.9(CH_2_), 114.2(C), 118.6(CH), 128.9(CH), 130.6(C), 136.3(CH), 143.4(C), 151.6(C), 158.8(C), 169.7(C), 205.7(CO) ppm. IR (KBr): 3328(m), 3246(m), 3114(w), 2903(s), 2849(m), 1627(s), 1590(s), 1574(s), 1508(s), 1473(m), 1407(m), 1370(w), 1322(m), 1271(w), 1239(m), 1174(m), 1057(m), 988(w), 930(w), 884(w), 844(w), 788(m), 750(w), 640(w) cm^−1^. DI-EI-MS *m*/*z* (%): 489(10), 488(M^+^, 24), 430(8), 411(23), 410(91), 408(7), 370(8), 355(22), 354(100), 353(39), 352(24), 342(19), 323(6), 296(28), 295(27), 266(9), 264(16), 253(6), 252(9), 250(12), 235(11), 136(11), 135(86), 120(9), 118(10), 107(18), 93(37), 91(16), 81(14), 79(38), 77(16), 73(12), 69(12), 67(19), 56(50), 55(57), 44(20), 43(28), 41(28). ESI-MS (pos.) *m*/*z* (%): 489.3 [M + H]^+^ (100). Anal. Calcd for C_28_H_36_N_6_O_2_: C 68.83; H 7.43; N 17.20. Found: C 69.02; H 7.35; N 17.14.

3-{[6-({4-[1-Adamantyl(hydroxy)methyl]phenyl}amino)-9-isopropyl-9*H*-purin-2-yl]amino}propan-1-ol (**4e**)

Prepared from **3e** (102 mg, 0.23 mmol) and 3-aminopropan-1-ol (138 mg, 1.84 mmol). Pale yellow crystalline powder, yield 89 mg (79%), mp 107–111 °C, R_f_ = 0.43 (system b). ^1^H NMR (CDCl_3_): *δ* 1.48–1.64 (m, 20H, CH(C**H**_3_)_2_ + CH_2_(Ad) + NHCH_2_C**H**_2_CH_2_OH), 1.95 (m, 3H, CH(Ad)), 3.02 (bs, 1H, CHO**H**), 3.63 (m, 4H, NHC**H**_2_CH_2_C**H**_2_OH), 4.16 (s, 1H, C**H**OH), 4.65 (septet, *J* = 6.3 Hz, 1H, C**H**(CH_3_)_2_), 5.22 (bs, 1H, NH(CH_2_)_3_O**H**), 7.18 (d, *J* = 8.3 Hz, 2H, Ph), 7.56–7.62 (d + s, 3H, Ph + NC^8^HN), 7.90 (s, 1H, C^6^NHPh) ppm. ^13^C NMR (CDCl_3_): *δ* 22.8(CH_3_), 28.6(CH), 33.6(CH_2_), 37.3(CH_2_), 37.5(C), 37.8(CH_2_), 38.4(CH_2_), 46.6(CH), 58.7(CH_2_), 82.7(CH), 115.0(C), 119.6(CH), 128.4(CH), 134.9(C), 138.4(CH), 138.0(C), 152.7(C), 160.2(C) ppm. IR (KBr): 3404–3345(bm), 3250(w), 3112(w), 2903(s), 2846(m), 1622(s), 1597(s), 1580(s), 1546(w), 1512(s), 1475(w), 1414(m), 1370(w), 1313(m), 1250(m), 1127(w), 1036(m), 1015(w), 936(w), 842(w), 788(m), 755(m), 641(w) cm^−1^. DI-EI-MS *m*/*z* (%): 491(12), 490(M^+^, 38), 356(22), 355(100), 354(8), 313(11), 267(9), 135(10), 93(6), 79(8), 44(6). ESI-MS (pos.) *m*/*z* (%): 491.5 [M + H]^+^ (100). Anal. Calcd for C_28_H_38_N_6_O_2_: C 68.54; H 7.81; N 17.13. Found: C 68.38; H 7.95; N 17.19.

3-{[6-{[4-(1-Adamantylmethyl)phenyl]amino}-9-isopropyl-9*H*-purin-2-yl]amino}propan-1-ol (**4f**)

Prepared from **3f** (101 mg, 0.23 mmol) and 3-aminopropan-1-ol (138 mg, 1.84 mmol). Pale brown crystalline powder, yield 81 mg (74%), mp 85–90 °C, R_f_ = 0.68 (system b). ^1^H NMR (CDCl_3_): *δ* 1.48–1.92 (m, 20H, CH(C**H**_3_)_2_ + CH_2_(Ad) + NHCH_2_C**H**_2_CH_2_OH+ CH(Ad)), 2.33 (s, 2H, PhC**H**_2_Ad), 3.64 (m, 4H, NHC**H**_2_CH_2_C**H**_2_OH), 4.65 (m, 2H, C**H**(CH_3_)_2_ + NH(CH_2_)_3_O**H**), 5.26 (bs, 1H, N**H**(CH_2_)_3_OH), 7.03 (d, *J* = 7.9 Hz, 2H, Ph), 7.57 (s, 1H, C^6^N**H**Ph), 7.62 (d, *J* = 7.6 Hz, 2H, Ph), 7.88 (s, 1H, NC^8^HN) ppm. ^13^C NMR (CDCl_3_): *δ* 22.8(CH_3_), 28.9(CH), 33.5(CH_2_), 33.7(C), 37.2(CH_2_), 37.8(CH_2_), 42.5(CH_2_), 46.5(CH), 50.8(CH_2_), 58.6(CH_2_), 115.0(C), 119.6(CH), 131.0(CH), 133.3(C), 134.9(CH), 136.9(C), 150.7(C), 152.7(C), 160.2(C) ppm. IR (KBr): 3328(m), 3243(w), 3116(w), 2901(s), 2844(m), 1622(s), 1595(s), 1579(s), 1545(w), 1511(s), 1477(w), 1414(m), 1370(w), 1313(m), 1251(m), 1215(w), 1127(w), 1046(w), 948(w), 884(w), 844(m), 787(m), 756(w), 640(w) cm^−1^. DI-EI-MS *m*/*z* (%): 475(33), 474(M^+^, 100), 473(7), 444(16), 443(33), 431(11), 430(27), 429(17), 417(5), 416(17), 401(6), 340(7), 339(33), 297(9), 295(16), 281(15), 251(5), 234(16), 135(20), 107(7), 106(25), 93(12), 79(14). ESI-MS (pos.) *m*/*z* (%): 475.5 [M + H]^+^ (100). Anal. Calcd for C_28_H_38_N_6_O: C 70.85; H 8.07; N 17.71. Found: C 70.96; H 8.24; N 17.59.

3-{[6-({3-[2-(1-Adamantyl)-1-hydroxyethyl]phenyl}amino)-9-isopropyl-9*H*-purin-2-yl]amino}propan-1-ol (**4g**)

Prepared from **3g** (100 mg, 0.21 mmol) and 3-aminopropan-1-ol (126 mg, 1.68 mmol). Pale yellow crystalline powder, yield 94 mg (89%), mp 87–91 °C, R_f_ = 0.42 (system b). ^1^H NMR (CDCl_3_): *δ* 1.51–1.74 (m, 22H, CH(C**H**_3_)_2_ + CH_2_(Ad) + PhCHC**H**_2_Ad+ NHCH_2_C**H**_2_CH_2_OH), 1.92 (m, 3H, CH(Ad)), 3.65 (m, 5H, NHC**H**_2_CH_2_C**H**_2_OH + CHO**H**), 4.64 (septet, *J* = 6.9 Hz, 1H, C**H**(CH_3_)_2_), 4.86 (m, 1H, C**H**OH), 5.27 (m, 1H, N**H**(CH_2_)_3_OH**)**, 6.96 (d, *J* = 7.6 Hz, 1H, Ph), 7.20 (t, *J* = 7.9 Hz, 1H, Ph), 7,25 (d, *J* = 7.9 Hz, 1H, Ph), 7.53 (s, 1H, Ph), 7.83 (s, 1H, NC^8^HN), 7.95 (s, 1H, C^6^N**H**Ph) ppm. ^13^C NMR (CDCl_3_): *δ* 22.8(CH_3_), 28.9(CH), 32.7(C), 33.5(CH_2_), 37.2(CH_2_), 38.1(CH_2_), 43.2(CH_2_), 46.5(CH), 54.0(CH_2_), 58.7(CH_2_), 70.7(CH), 114.9(C), 118.2(CH), 119.2(CH), 121.0(CH), 128.9(CH), 135.0(CH), 139.1(C), 148.0(C), 150.5(C), 152.6(C), 160.2(C) ppm. IR (KBr): 3333(m), 3247(w), 3119(w), 2900(s), 2845(m), 1625(s), 1584(s), 1524(m), 1484(m), 1443(w), 1370(w), 1324(w), 1254(m), 1215(w), 1131(w), 1064(m), 1112(w), 884(w), 787(m), 756(m), 641(w) cm^−1^. DI-EI-MS *m*/*z* (%): 505(34), 504(M^+^, 100), 473(6), 461(5), 460(11), 455(11), 356(8), 355(22), 325(7), 313(7), 312(11), 311(18), 269(5), 267(9), 234(25), 136(6), 35(67), 107(9), 93(17), 79(16). ESI-MS (pos.) *m*/*z* (%): 505.5 [M + H]^+^ (100). Anal. Calcd for C_29_H_40_N_6_O_2_: C 69.02; H 7.99; N 16.65. Found: C 69.09; H 7.93; N 16.78.

3-{[6-({3-[2-(1-Adamantyl)ethyl]phenyl}amino)-9-isopropyl-9*H*-purin-2-yl]amino}propan-1-ol (**4h**)

Prepared from **3h** (72 mg, 0.16 mmol) and 3-aminopropan-1-ol (96 mg, 1.28 mmol). Colourless crystalline powder, yield 63 mg (80%), mp 72–78 °C, R_f_ = 0.71 (system b). ^1^H NMR (CDCl_3_): *δ* 1.40–1.79 (m, 22H, CH(C**H**_3_)_2_ + CH_2_(Ad) + PhCH_2_C**H**_2_Ad+ NHCH_2_C**H**_2_CH_2_OH), 2.00 (m, 3H, CH(Ad)), 2.59 (t, *J* = 7.9 Hz, 2H, PhC**H**_2_CH_2_Ad), 3.69 (m, 4H, NHC**H**_2_CH_2_C**H**_2_OH), 4.69 (m, 2H, C**H**(CH_3_)_2_ + NH(CH_2_)_3_O**H**), 5.28 (bs, 2H, N**H**(CH_2_)_3_OH**)**, 6.93 (d, *J* = 6.6 Hz, 1H, Ph), 7.27 (t, *J* = 7.3 Hz, 1H, Ph), 7.53 (s, 1H, C^6^N**H**Ph), 7.63–7.71 (m, 3H, Ph + NC^8^HN) ppm. ^13^C NMR (CDCl_3_): *δ* 22.9(CH_3_), 29.0(CH), 29.4(CH_2_), 32.7(C), 33.7(CH_2_), 37.5(CH_2_), 37.8(CH_2_), 42.7(CH_2_), 46.6(CH_2_), 46.8(CH), 58.7(CH_2_), 115.2(C), 117.8(CH), 118.9(C), 120.4(CH), 123.6(CH), 128.9(CH), 135.0(C), 139.0(CH), 144.9(C), 152.8(C), 155.1(C), 160.2(C) ppm. IR (KBr): 3324(m), 3246(w), 3124(w), 2901(s), 2844(m), 1613(m), 1592(s), 1523(m), 1488(m), 1418(w), 1370(w), 1323(s), 1247(m), 1215(w), 1127(w), 1046(m), 1013(w), 934(w), 885(w), 787(m), 756(w), 696(w), 669(w), 641(w) cm^−1^. DI-EI-MS *m*/*z* (%): 489(34), 488(M^+^, 100), 487(10), 458(17), 457(35), 445(11), 444(24), 443(23), 415(9), 401(6), 353(10), 309(11), 296(16), 295(7), 282(12), 253(8), 251(5), 234(21), 135(13), 107(6), 93(11), 91(5), 79(13). ESI-MS (pos.) *m*/*z* (%): 489.5 [M + H]^+^ (100). Anal. Calcd for C_29_H_40_N_6_O: C 71.28; H 8.25; N 17.20. Found: C 71.19; H 8.20; N 17.29.

2-(1-Adamantyl)-1-{3-[({2-(3-hydroxypropyl)amino]-9-isopropyl-9*H*-purin-6-yl}amino)methyl]phenyl}ethan-1-one (**4i**)

Prepared from **3i** (100 mg, 0.21 mmol) and 3-aminopropan-1-ol (126 mg, 1.68 mmol). Colourless crystalline powder, yield 87 mg (80%), mp 64–69 °C, R_f_ = 0.16 (system b). ^1^H NMR (CDCl_3_): *δ* 1.49–1.73 (m, 20H, CH(C**H**_3_)_2_ + (CH_2_)Ad + NHCH_2_C**H**_2_CH_2_OH), 1.89 (m, 3H, (CH)Ad), 2.66 (s, 2H, PhCOC**H**_2_Ad), 3.62 (m, 4H, NHC**H**_2_CH_2_C**H**_2_OH), 4.60 (septet, *J* = 6.6 Hz, 1H, C**H**(CH_3_)_2_), 4.81 (s, 2H, C^6^NHC**H**_2_Ph), 5.11 (bs, 1H, N**H**(CH_2_)_3_OH), 6.46 (bs, 1H, C^6^N**H**CH_2_Ph), 7.34–7.52 (m, 3H, Ph), 7.81 (s, 1H, Ph), 7.95 (s, 1H, NC^8^HN) ppm. ^13^C NMR (CDCl_3_): *δ* 22.8(CH_3_), 28.9(CH), 33.7(CH_2_), 34.1(C), 36.9(CH_2_), 37.7(CH_2_), 43.1(CH_2_), 46.4(CH), 51.5(CH_2_), 58.5(CH_2_), 114.6(C), 127.5(CH), 127.9(CH), 128.8(CH), 132.1(CH), 134.5(C), 139.2(CH), 139.8(C), 151.6(C), 155.1(C), 160.4(C), 200.2(CO) ppm. IR (KBr): 3322(m), 3249(w), 3120(w), 2903(s), 2847(m), 1670(m), 1601(s), 1496(s), 1450(w), 1391(w), 1371(w), 1346(w), 1288(w), 1255(m), 1216(w), 1137(w), 1069(m), 976(w), 884(w), 788(m), 757(w), 696(w), 644(w) cm^−1^. DI-EI-MS *m*/*z* (%): 517(36), 516(M^+^, 100), 515(6), 487(5), 486(15), 485(11), 473(13), 472(28), 471(17), 458(11), 381(9), 249(6), 135(31), 134(6), 119(16), 93(6), 91(9), 79(9), 73(6), 60(6), 45(14), 44(14). ESI-MS (pos.) *m*/*z* (%): 517.5 [M + H]^+^ (100). Anal. Calcd for C_30_H_40_N_6_O_2_: C 69.74; H 7.80; N 16.27. Found: C 69.58; H 7.84; N 16.36.

2-(1-Adamantyl)-1-{4-[({2-(3-hydroxypropyl)amino]-9-isopropyl-9*H*-purin-6-yl}amino)methyl]phenyl}ethan-1-one (**4j**)

Prepared from **3j** (100 mg, 0.21 mmol) and 3-aminopropan-1-ol (126 mg, 1.68 mmol). Colourless crystalline powder, yield 86 mg (79%), mp 66–60 °C, R_f_ = 0.23 (system b). ^1^H NMR (CDCl_3_): *δ* 1.50–1.69 (m, 20H, CH(C**H**_3_)_2_ + CH_2_(Ad) + NHCH_2_C**H**_2_CH_2_OH), 1.92 (m, 3H, CH(Ad)), 2.67 (s, 2H, PhCOC**H**_2_Ad), 3.61 (m, 4H, NHC**H**_2_CH_2_C**H**_2_OH), 4.60 (m, 1H, C**H**(CH_3_)_2_), 4.81 (bs, 2H, C^6^NHC**H**_2_Ph), 5.04 (bs, 1H, N**H**(CH_2_)_3_OH), 6.46 (bs, 1H, C^6^N**H**CH_2_Ph), 7.41 (m, 3H, Ph + NC^8^HN), 7.87 (d, *J* = 7.6 Hz, 2H, Ph) ppm. ^13^C NMR (CDCl_3_): *δ* 22.8(CH_3_), 28.9(CH), 33.6(CH_2_), 34.1(C), 36.9(CH_2_), 37.7(CH_2_), 43.2(CH_2_), 46.4(CH), 51.4(CH_2_), 58.5(CH_2_), 115.2(C), 127.6(CH), 128.9(CH), 134.6(C), 138.1(CH), 144.4(C), 155.1(C), 160.4(C), 199.9(CO) ppm. IR (KBr): 3316(m), 3242(w), 3115(w), 2902(s), 2847(m), 1668(m), 1600(s), 1496(s), 1409(w), 1390(w), 1370(w), 1346(w), 1260(m), 1214(w), 1133(w), 1067(m), 1014(w), 977(w), 788(m), 670(w), 642(w) cm^−1^. DI-EI-MS *m*/*z* (%): 517(35), 516(M^+^, 100), 515(5), 486(13), 485(20), 473(8), 472(23), 471(13), 458(9), 382(5), 381(21), 337(5), 249(8), 135(35), 134(7), 133(9), 119(5), 107(9), 93(14), 91(11), 79(15). ESI-MS (pos.) *m*/*z* (%): 517.6 [M + H]^+^ (100). Anal. Calcd for C_30_H_40_N_6_O_2_: C 69.74; H 7.80; N 16.27. Found: C 69.82; H 7.71; N 16.19.

3-{[6-(Benzylamino)-9-isopropyl-9*H*-purin-2-yl]amino}propan-1-ol (**4k**)

Prepared from **3k** (75 mg, 0.25 mmol) and 3-aminopropan-1-ol (150 mg, 2.00 mmol). Pale yellow crystalline powder, yield 75 mg (88%), mp 85–89 °C, R_f_ = 0.43 (system b). ^1^H NMR (CDCl_3_): *δ* 1.53 (d, *J* = 6.9 Hz, 6H, CH(C**H**_3_)_2_), 1,74 (quintet, *J* = 5.6 Hz, 2H, NHCH_2_C**H**_2_CH_2_OH), 3.63 (m, 4H, NHC**H**_2_CH_2_C**H**_2_OH), 4.62 (septet, *J* = 6.9 Hz, 1H, C**H**(CH_3_)_2_), 4.79 (s, 2H, C^6^NHC**H**_2_Ph), 5.10 (bs, 2H, N**H**(CH_2_)_3_O**H**), 6.38 (bs, 1H, C^6^N**H**CH_2_Ph), 7.26–7.38 (m, 5H, Ph), 7.44 (s, 1H, NC^8^HN) ppm. ^13^C NMR (CDCl_3_): *δ* 22.9(CH_3_), 33.7(CH_2_), 37.6(CH_2_), 46.4(CH), 58.5(CH_2_), 114.6(C), 127.5(CH), 127.9(CH), 128.8(CH), 134.5(CH), 139.1(C), 155.1(C), 155.6(C), 160.4(C) ppm. IR (KBr): 3400(m), 3268–3206(bm), 3125(w), 2972–2873(bw), 1623(s), 1600(s), 1523(s), 1474(w), 1390(m), 1341(w), 1292(m), 1260(m), 1220(w), 1130(w), 1183(w), 1065(m), 1026(w), 972(m), 886(w), 787(m), 745(m), 726(w), 696(m), 639(m), 543(w) cm^−1^. DI-EI-MS *m*/*z* (%): 341(13), 340(M^+^, 58), 310(9), 309(21), 297(9), 296(23), 295(20), 282(10), 267(5), 253(8), 239(5), 191(11), 177(7), 149(7), 135(9), 134(10), 119(5), 108(7), 107(6), 106(20), 92(10), 91(100), 65(10), 57(6), 55(5), 43(15), 41(10). ESI-MS (pos.) *m*/*z* (%): 341.3 [M + H]^+^ (100). Anal. Calcd for C_18_H_24_N_6_O: C 63.51 H 7.11; N 24.69. Found: C 63.28; H 7.21; N 24.76.

### 3.6. CDK Inhibition Assay

CDK2/cyclin E activity was assayed as previously described [11,15]. Briefly, the kinase was assayed with [γ-^33^P]ATP and suitable peptide substrates in a reaction buffer (60 mM HEPES-NaOH, pH 7.5, 3 mM MgCl_2_, 3 mM MnCl_2_, 3 μM Na-orthovanadate, 1.2 mM DTT, 2.5 μg/50 μL PEG_20.000_). The reactions were stopped by adding 5 µL of 3% aq. H_3_PO_4_. Aliquots were spotted onto P-81 phosphocellulose, washed with 0.5% aq. H_3_PO_4_ and air-dried. Kinase inhibition was quantified using an FLA-7000 digital image analyser. The concentration of the test compound required to reduce the kinase activity by 50% was determined from dose–response curves and reported as the IC_50_ value.

### 3.7. In Vitro Cytotoxicity

Cell lines K562 and MCF-7 were obtained from the European Collection of Cell Cultures. The cell lines were cultivated in a Dulbecco’s Modified Eagle’s medium supplemented with 10% fetal bovine serum, penicillin (100 U/mL) and streptomycin (100 μg/mL) at 37 °C in 5% CO_2_. For the viability assays, cells were seeded into 96-well plates (5000 cells per well), and after the preincubation period, were treated in triplicates with six different doses of each compound for 72 h. After treatments, a resazurin (Sigma-Aldrich, St Louis, MO, USA) solution was added for four hours, and the fluorescence of resorufin formed in live cells was measured at 544 nm/590 nm (excitation/emission) using a Fluoroskan Ascent microplate reader (Labsystems, Finland). The GI_50_ value, a drug concentration lethal to 50% of the cells, was calculated from the dose–response curves.

## 4. Conclusions

Our initial motivation was to investigate if the adamantane scaffold (Ad) can be used to improve purine-based CDK inhibitors. We assumed there would be improved water solubility and related bioavailability of Ad-modified drugs via complexation with suitable macrocyclic carriers. We prepared a series of ten new 2,6,9-trisubstituted purines bearing isopropyl, 3-hydroxypropylamino and various adamantylated anilines and benzylamines in positions 9, 2 and 6, respectively. Although previous studies demonstrated that substituting purine at position 6 with adamantylamine would switch off the inhibition activity towards CDK1 and CDK2, we reached IC_50_(CDK2/E) = 0.21 ± 0.02 μM for adamatylated purine **4b**, which is even lower than that of its parent, bohemine **4k**. In concert with the supramolecular nature of the interaction between adamantylated purines and CD carries, we showed that IC_50_ (CDK2/E) and GI_50_ (K-562, MCF-7) increased with a portion of β-CD in tested mixtures. On the other hand, the application of CD carriers allowed us to determine the GI_50_ values, even in the cases of sparingly soluble purines. In addition, we performed a docking study to clarify that the inactivity of previously published adamantylated purines is not related to the presence of the bulky adamantane moiety, which was believed to prevent the entrance of the inhibitor to the active site. We demonstrated that entire molecules of purine derivatives with the adamantane cage, linked via appropriately long spacers, can be buried inside the binding pocket of CDK2 to form tight complexes. We believe that our results will encourage scientists to use the adamantane scaffold for improving drug properties.

## Figures and Tables

**Table 1:** CDK inhibition activity of purines **4a**–**l** and their complexes with β-CD.

Compound	CDK2/Cyclin E IC_50_ (μm) ^1^		
	Purine	Purine·β-CD (1:1)	Purine·β-CD (1:10)
**4a**	2.55 ± 0.08	5.5 ± 1.3	>40
**4b**	0.21 ± 0.02	0.16	0.3 ± 0.2
**4c**	12 ± 8	1.85	>40
**4d**	0.6 ± 0.2	1.4 ± 0.5	7.57
**4e**	1.6 ± 0.7	1.53	26.1
**4f**	>12.5	>40	>40
**4g**	0.92 ± 0.18	1.24	7.15
**4h**	>12.5	>40	>40
**4i**	14 ± 5	>40	30.7
**4j**	3.26 ± 0.01	15.7	20.9
**4k** (bohemine)	0.7 ± 0.5	1.1 ± 0.6	1.11 ± 0.12
**4l** (roscovitine)	0.22 ^2^	n.a. ^3^	n.a. ^3^

^1^ IC_50_ values are expressed as the mean ± SD (standard deviation) of duplicated experiments. ^2^ The value of roscovitine (**4l**) was taken from Jorda et al. [19]. ^3^ Not available.

**Table 2:** In vitro antiproliferative activity of purines **4a**–**l** and their complexes with β-CD.

Compound	K-562 GI_50_ (μm) ^1^			MCF-7 GI_50_ (μm) ^1^		
	Purine	Purine·β-CD (1:1)	Purine·β-CD (1:10)	Purine	Purine·β-CD (1:1)	Purine·β-CD (1:10)
**4a**	6.16	15.04 ± 0.16	>40	>6.25	15.7 ± 0.9	>40
**4b**	>6.25	34.69 ± 0.14	>40	>6.25	32 ± 3	>40
**4c**	>6.25	28.7 ± 10.2	>40	>6.25	>40	>40
**4d**	5.45	16.5 ± 0.7	>40	5.72	13.8 ± 1.4	>40
**4e**	10.1 ± 0.8	18.7 ± 0.6	>40	8.1 ± 0.7	17 ± 2	>40
**4f**	10.4 ± 0.7	24 ± 3	>40	> 12.5	32.9 ± 0.1	>40
**4g**	>6.25	33.6 ± 1.2	>40	>6.25	17 ± 2	>40
**4h**	>12.5	>40	>40	>12.5	>40	>40
**4i**	>6.25	19.8 ± 0.5	>40	>6.25	30 ± 5	>40
**4j**	>6.25	17.7 ± 0.6	>40	>6.25	21.51 ± 0.04	>40
**4k** (bohemine)	96.55	>40	>40	26.73 ± 6.36	36 ± 4	35 ± 4
**4l** (roscovitine)	50.1 ^2^	n.a. ^3^	n.a. ^3^	12.6 ^2^	n.a. ^3^	n.a. ^3^

^1^ GI_50_ values are expressed as the mean ± SD of duplicated experiments. Tumour cell lines: K-562, human chronic myelogenous leukaemia; MCF-7, human breast adenocarcinoma. ^2^ The values of roscovitine were taken from Popowycz et al. [51]. ^3^ Not available.

## Supplementary Materials

The following are available online at https://www.mdpi.com/article/10.3390/ijms222312675/s1.
