# Supplementary material for: Adamantane-Substituted Purines and Their β-Cyclodextrin Complexes: Synthesis and Biological Activity
Source: Int J Mol Sci. 2021 Nov 24;22(23):12675. doi: 10.3390/ijms222312675 (PMC8657435; doi:10.3390/ijms222312675)
Supplement: Supplementary file 1 [file ijms-22-12675-s001.zip › ijms-1466727-supplementary.pdf]

Adamantane-substituted purines and their β-cyclodextrin complexes: Synthesis and biological activity

Michal Rouchal[a], Jana Rudolfová[a], Vladimír Kryštof[b], Veronika Vojáčková[b], Richard Čmelík[c], and Robert Vícha[a,*]

[a] *Department of Chemistry, Faculty of Technology, Tomas Bata University in Zlín, Vavrečkova 275, 760 01 Zlín, Czech Republic*

[b] *Department of Experimental Biology, Palacký University, Šlechtitelů 27, 783 71 Olomouc, Czech Republic*

[c] *Institute of Analytical Chemistry, v.v.i., Academy of Sciences of the Czech Republic, Veveří 97, 602 00 Brno, Czech Republic*

*Corresponding author: email: rvicha@utb.cz

Supporting Information

Table of Contents

NMR and MS spectra of compounds **4a–k** S2

ESI-MS spectra of equimolar mixtures of purines **4** with β-CD S22

Molecular docking results S32

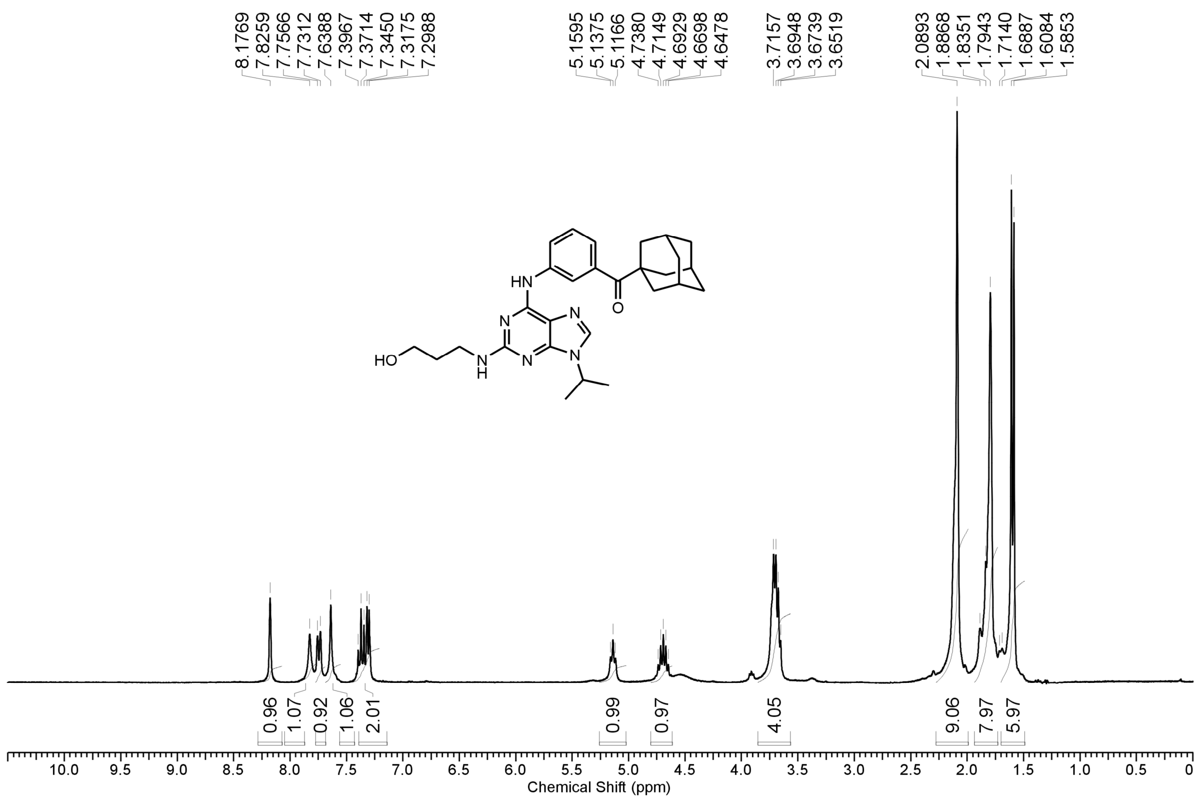

Figure S1 ^{1}H NMR spectrum (CDCl$_3$, 300 MHz, 303 K) of compound **4a**.

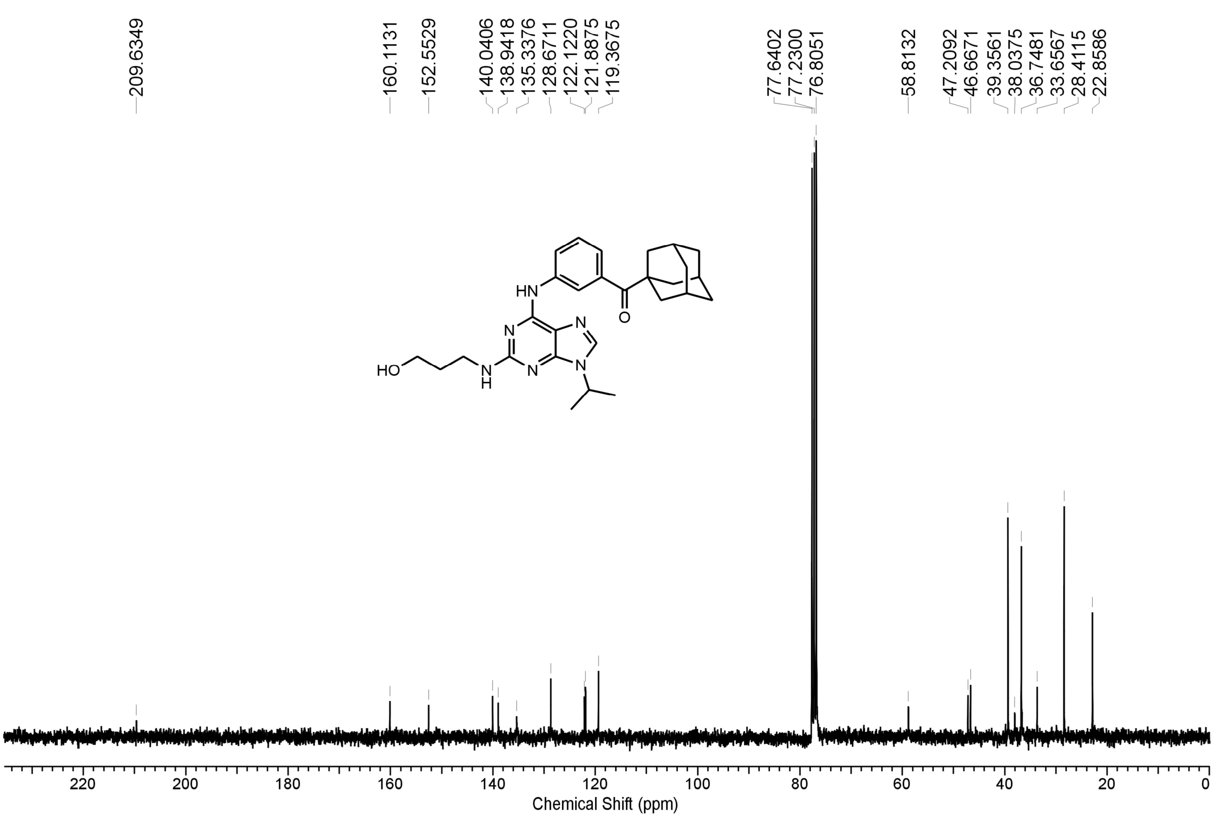

Figure S2 ^{13}C NMR spectrum (CDCl$_3$, 75 MHz, 303 K) of compound **4a**.

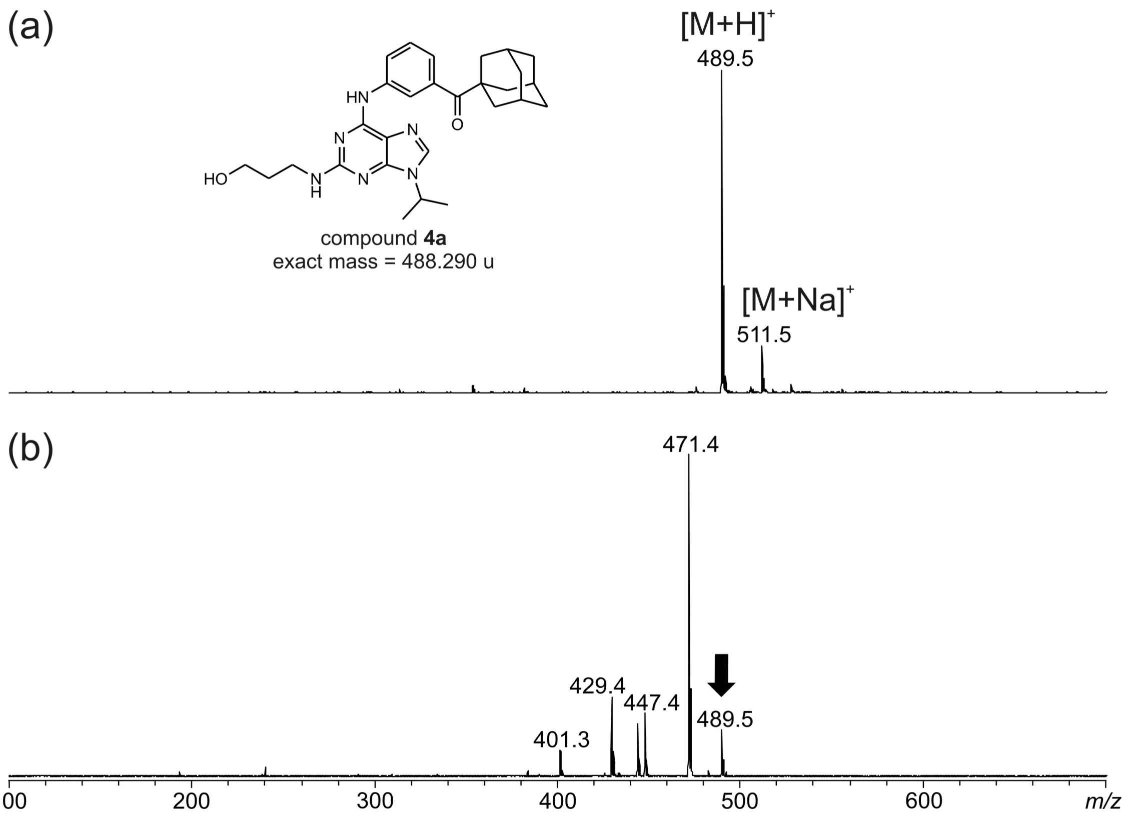

Figure S3 Positive-ion mode ESI mass spectra (full scan) of compound **4a**; (a) first-order mass spectra, (b) MS/MS of *m/z* 489. The assignments for the observed ions are shown in the brackets. The fragmented ion in tandem mass spectrum is marked with bold, downward arrow.

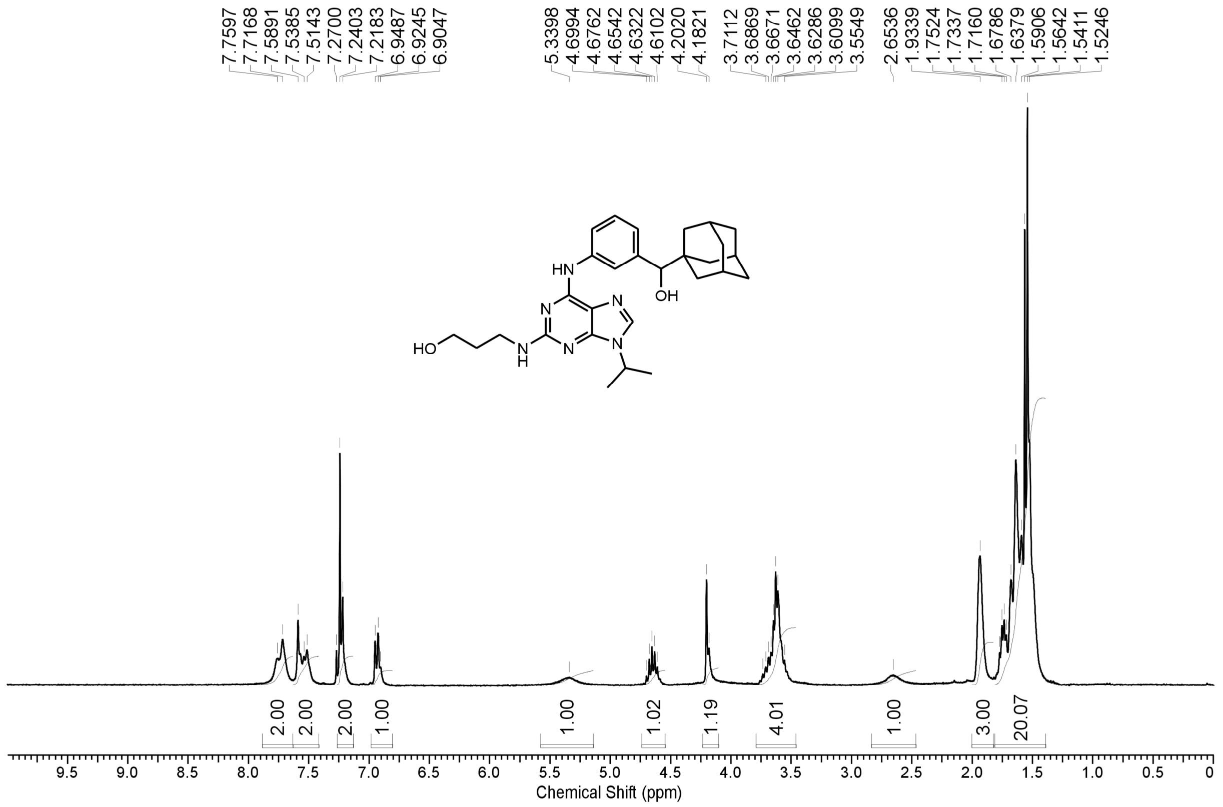

Figure S4 ^{1}H NMR spectrum ($CDCl_3$, 300 MHz, 303 K) of compound **4b**.

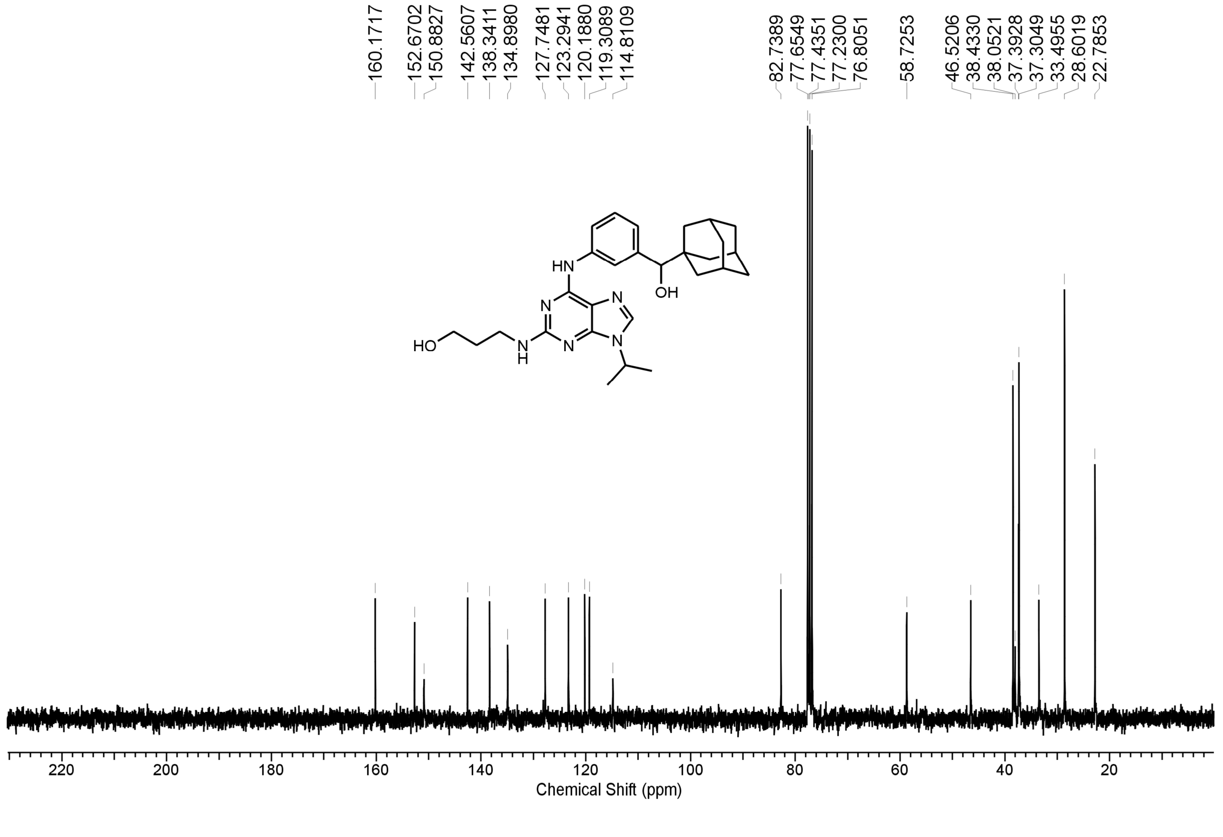

Figure S5 ^{13}C NMR spectrum ($CDCl_3$, 75 MHz, 303 K) of compound **4b**.

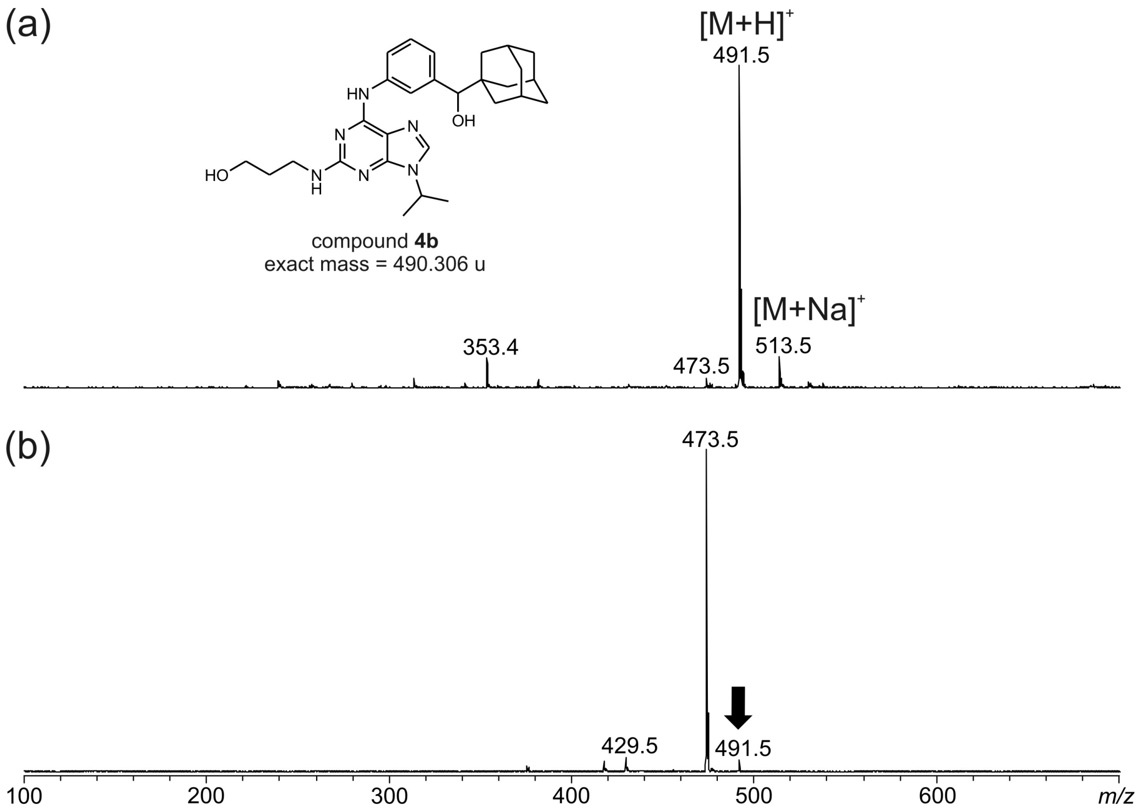

Figure S6 Positive-ion mode ESI mass spectra (full scan) of compound **4b**; (a) first-order mass spectra, (b) MS/MS of *m/z* 491. The assignments for the observed ions are shown in the brackets. The fragmented ion in tandem mass spectrum is marked with bold, downward arrow.

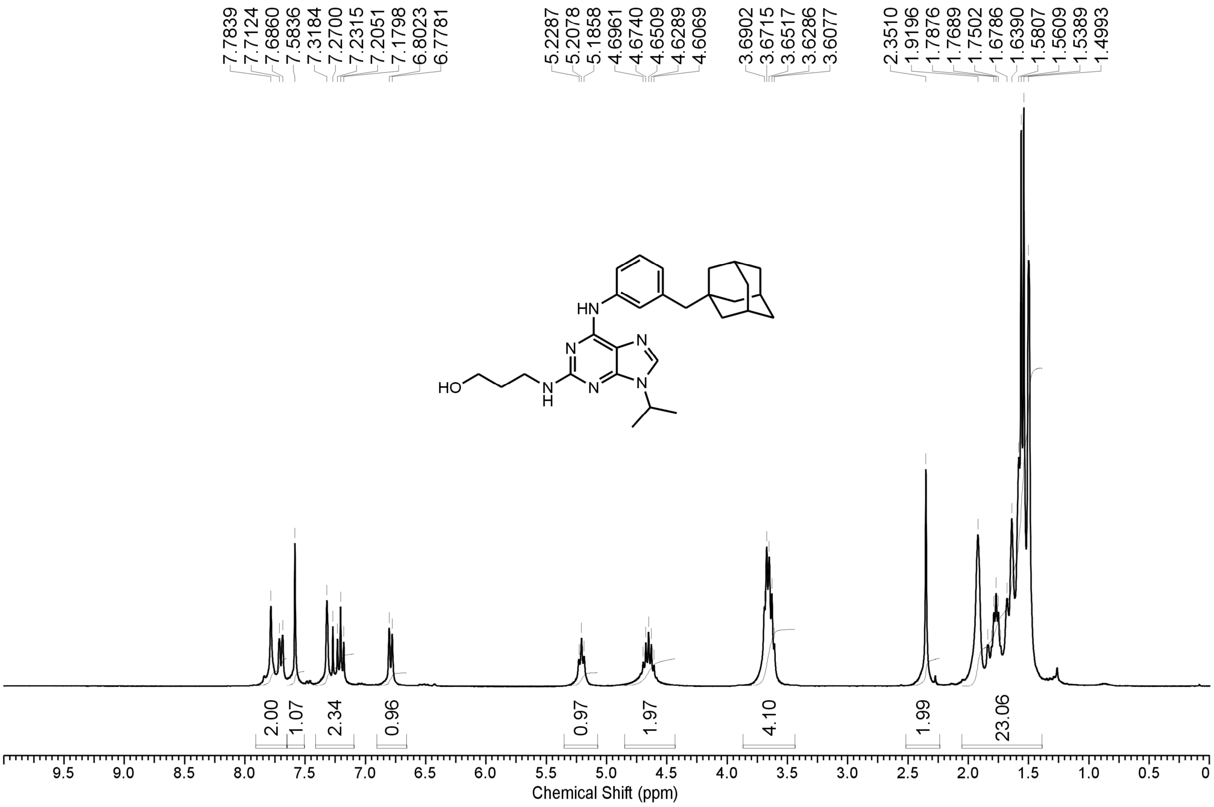

Figure S7 ^{1}H NMR spectrum ($CDCl_3$, 300 MHz, 303 K) of compound **4c**.

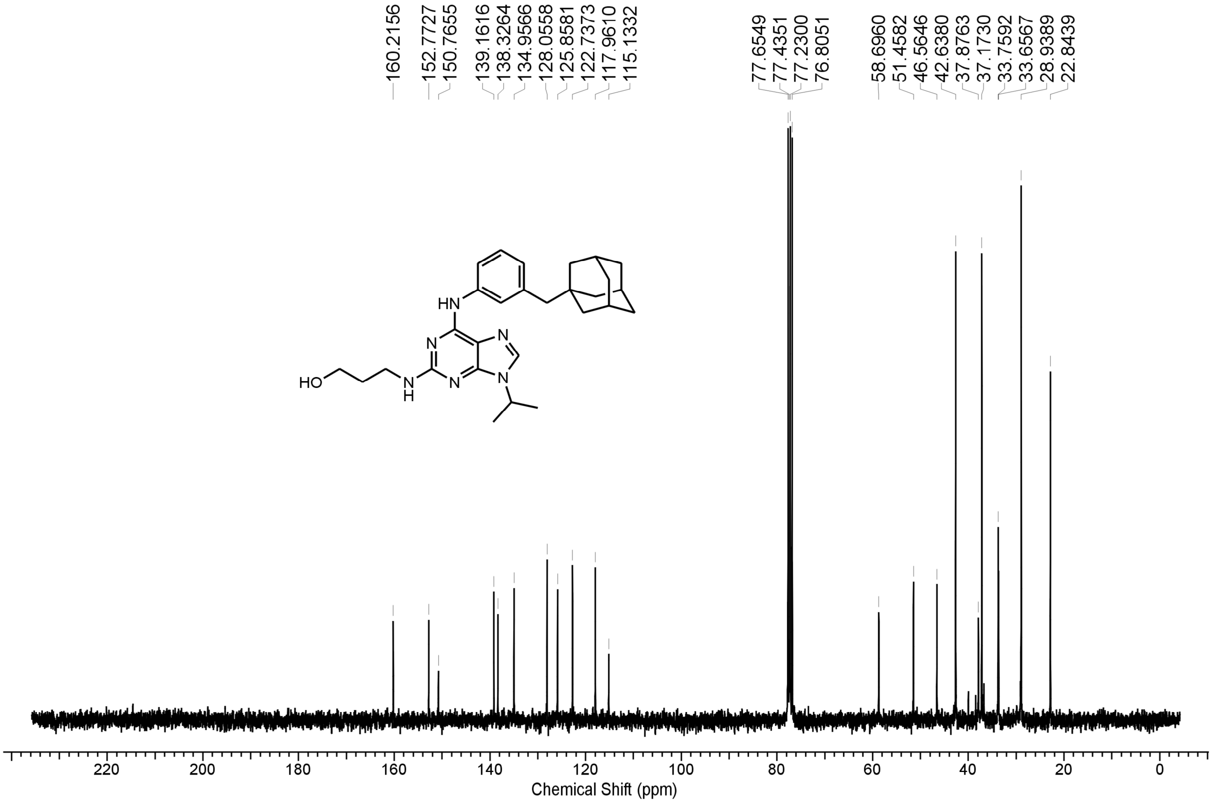

Figure S8 ^{13}C NMR spectrum ($CDCl_3$, 75 MHz, 303 K) of compound **4c**.

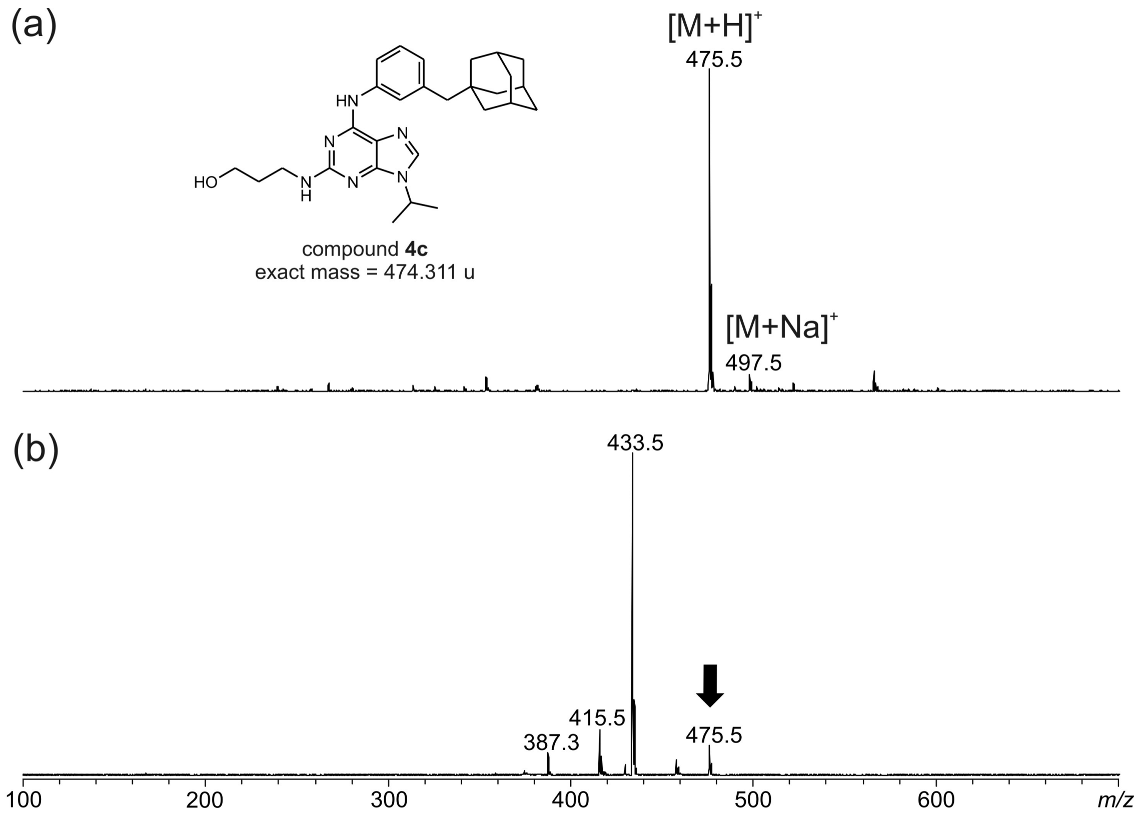

Figure S9 Positive-ion mode ESI mass spectra (full scan) of compound **4c**; (a) first-order mass spectra, (b) MS/MS of *m/z* 475. The assignments for the observed ions are shown in the brackets. The fragmented ion in tandem mass spectrum is marked with bold, downward arrow.

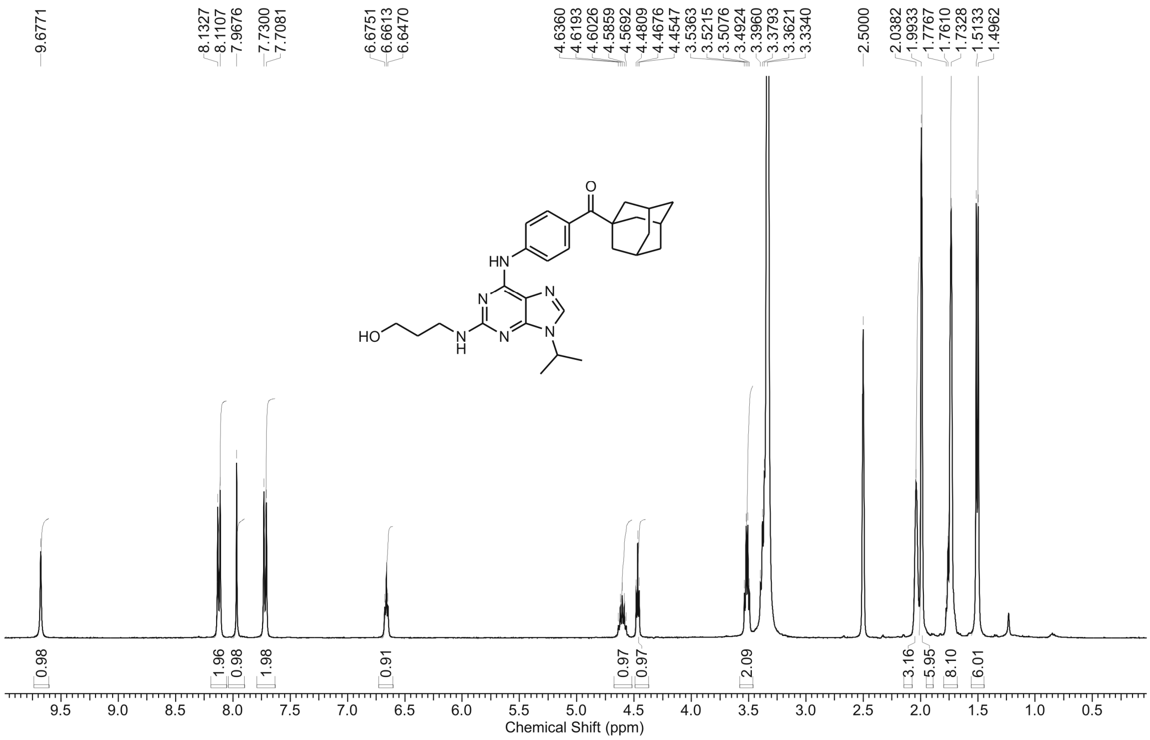

Figure S10 ^{1}H NMR spectrum (DMSO-d_6, 400 MHz, 303 K) of compound **4d**.

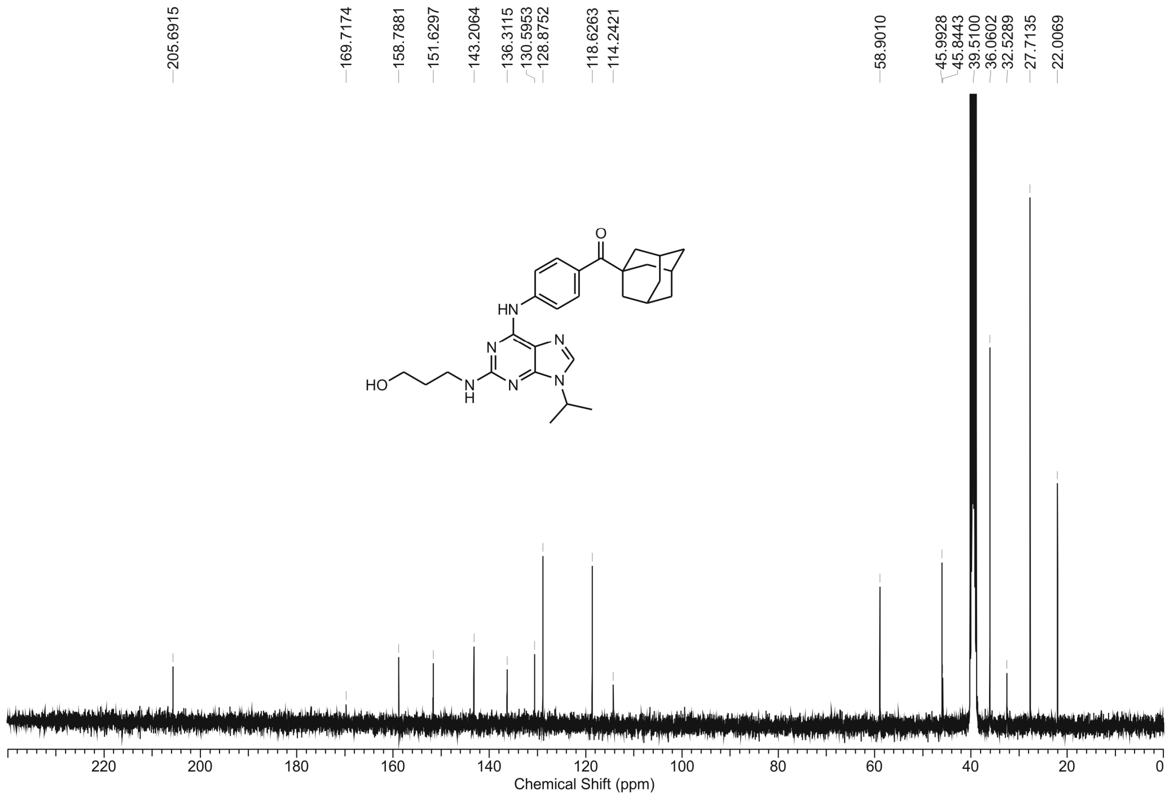

Figure S11 ^{13}C NMR spectrum (DMSO-d_6, 101 MHz, 303 K) of compound **4d**.

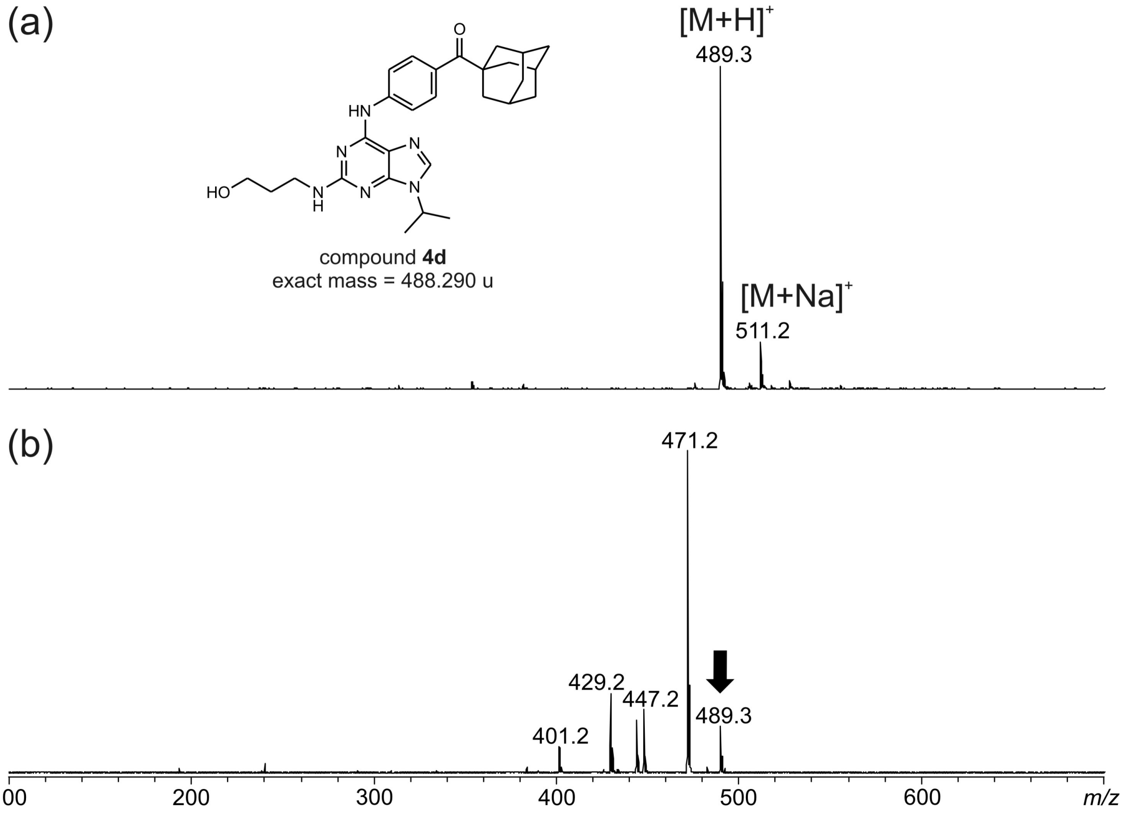

Figure S12 Positive-ion mode ESI mass spectra (full scan) of compound **4d**; (a) first-order mass spectra, (b) MS/MS of *m/z* 489. The assignments for the observed ions are shown in the brackets. The fragmented ion in tandem mass spectrum is marked with bold, downward arrow.

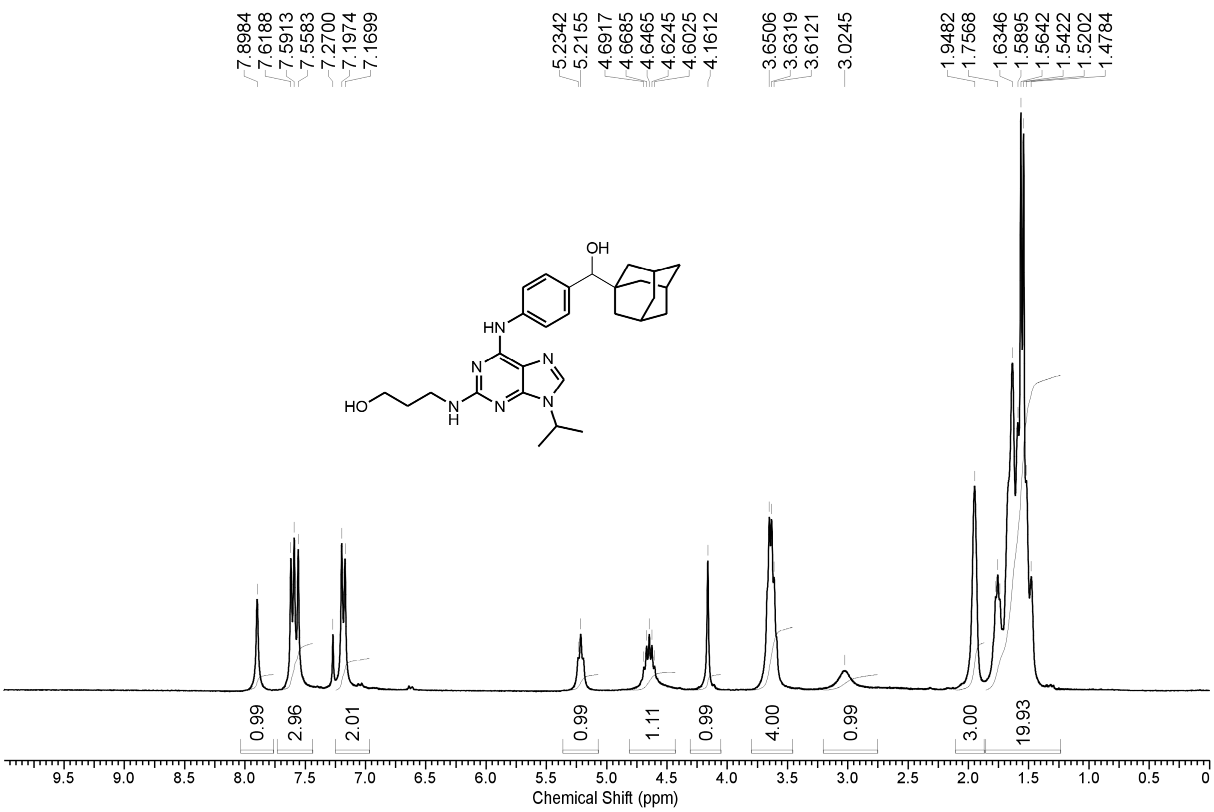

Figure S13 ^{1}H NMR spectrum ($CDCl_3$, 300 MHz, 303 K) of compound **4e**.

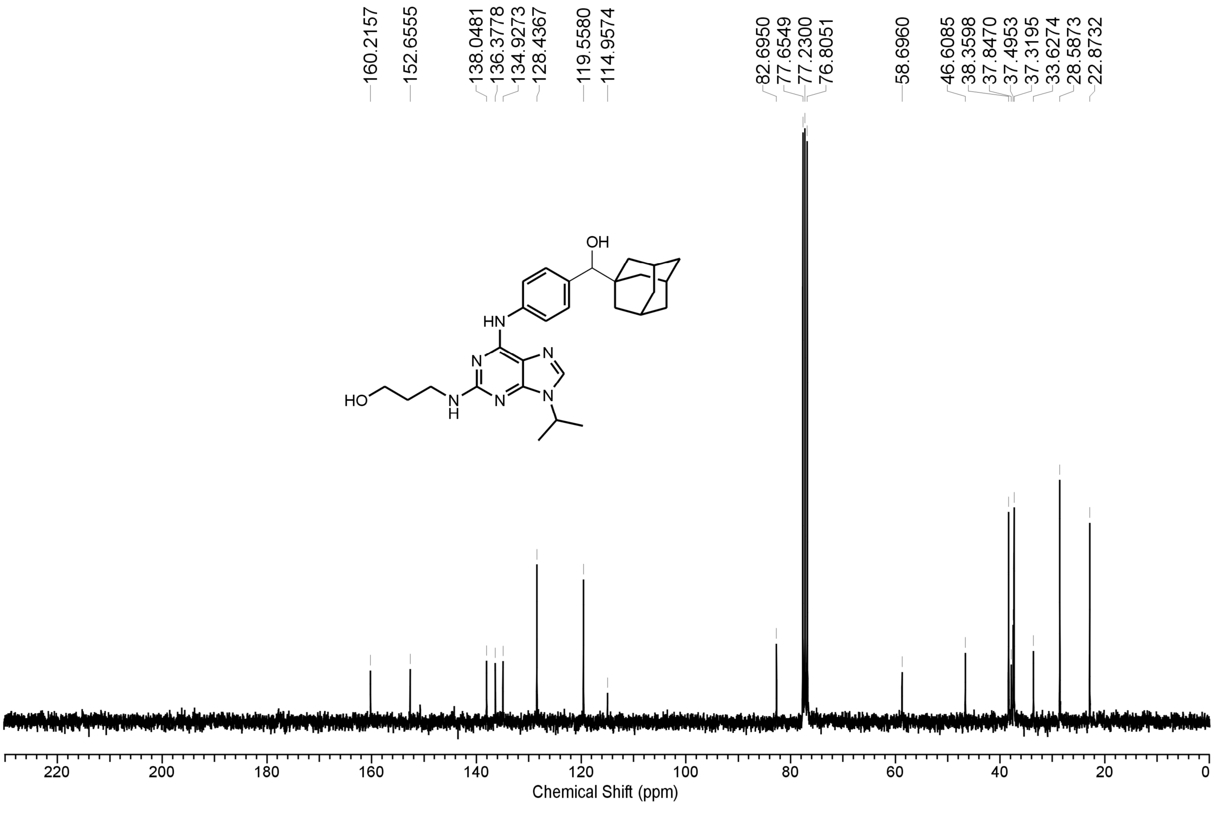

Figure S14 ^{13}C NMR spectrum ($CDCl_3$, 75 MHz, 303 K) of compound **4e**.

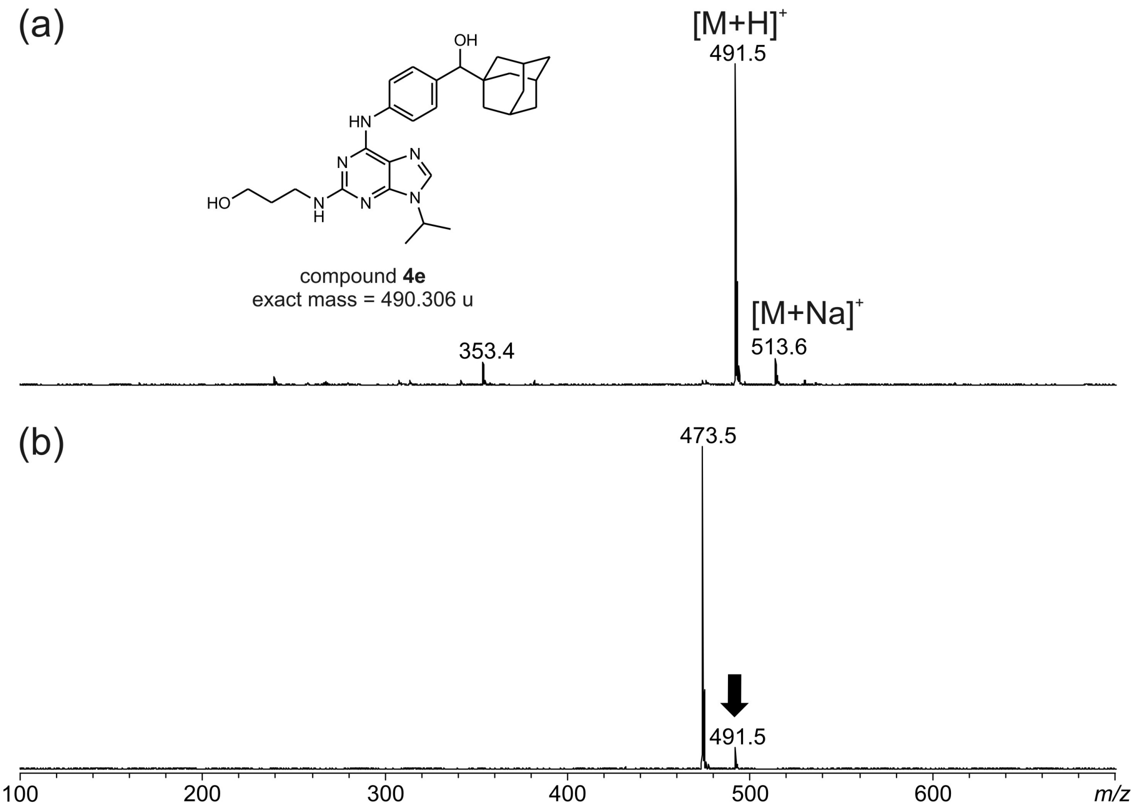

Figure S15 Positive-ion mode ESI mass spectra (full scan) of compound **4e**; (a) first-order mass spectra, (b) MS/MS of *m/z* 491. The assignments for the observed ions are shown in the brackets. The fragmented ion in tandem mass spectrum is marked with bold, downward arrow.

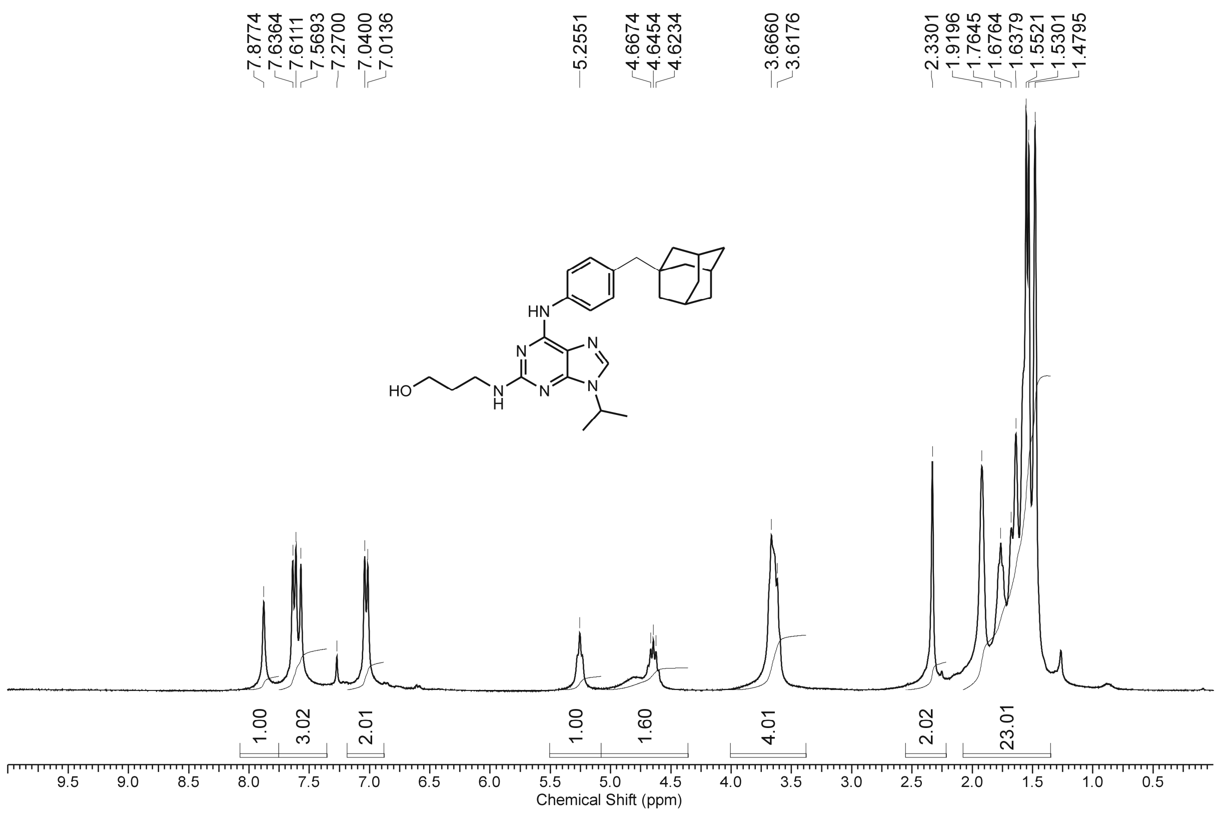

Figure S16 ^{1}H NMR spectrum ($CDCl_3$, 300 MHz, 303 K) of compound **4f**.

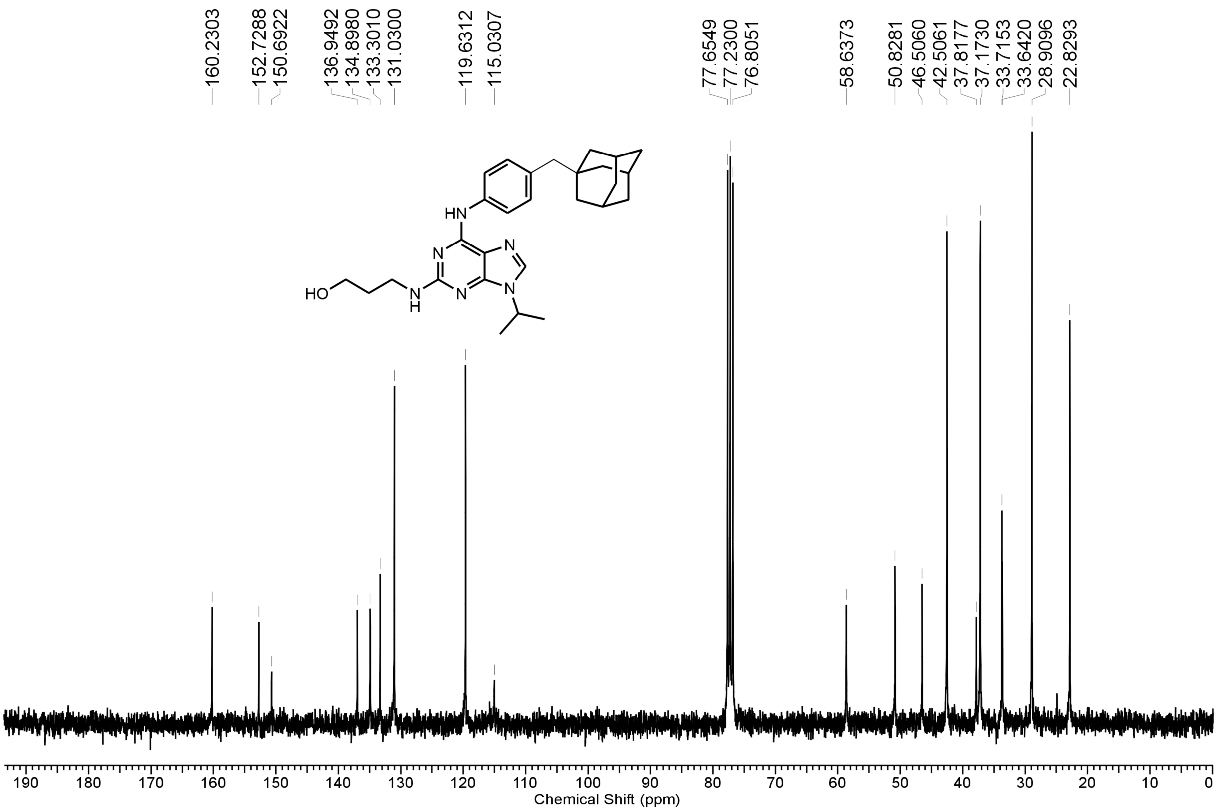

Figure S17 ^{13}C NMR spectrum ($CDCl_3$, 75 MHz, 303 K) of compound **4f**.

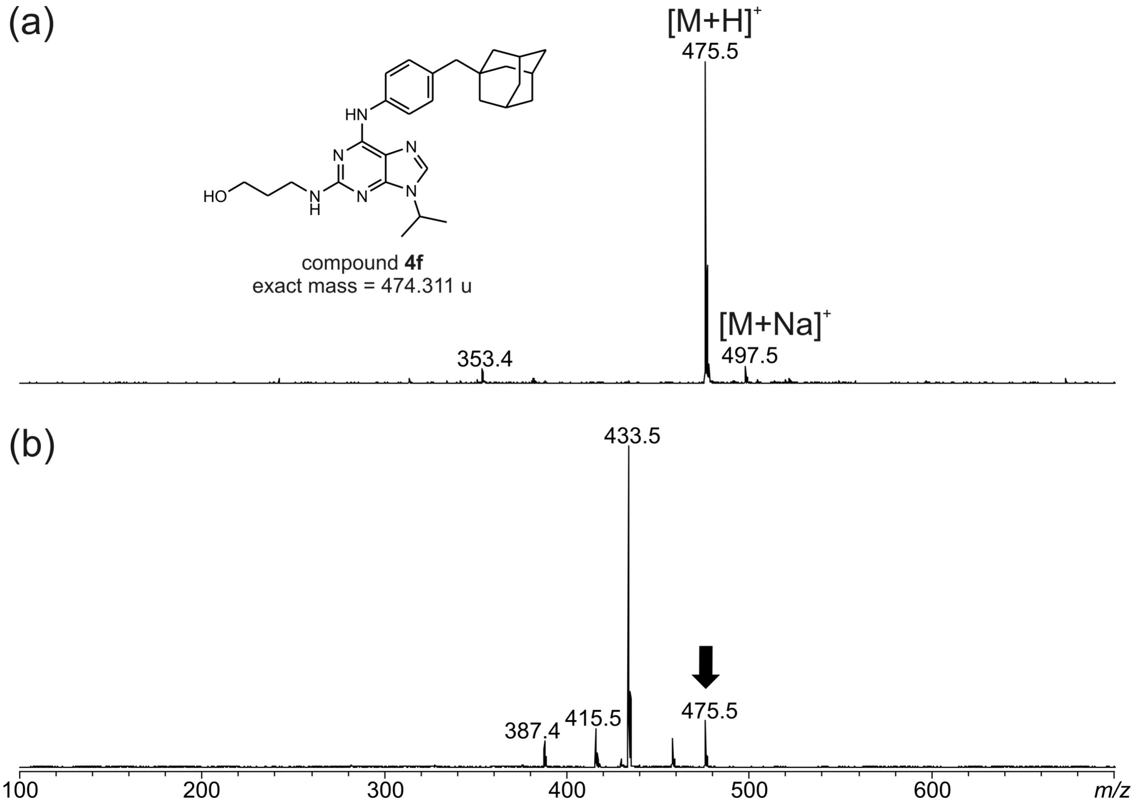

Figure S18 Positive-ion mode ESI mass spectra (full scan) of compound **4f**; (a) first-order mass spectra, (b) MS/MS of *m/z* 475. The assignments for the observed ions are shown in the brackets. The fragmented ion in tandem mass spectrum is marked with bold, downward arrow.

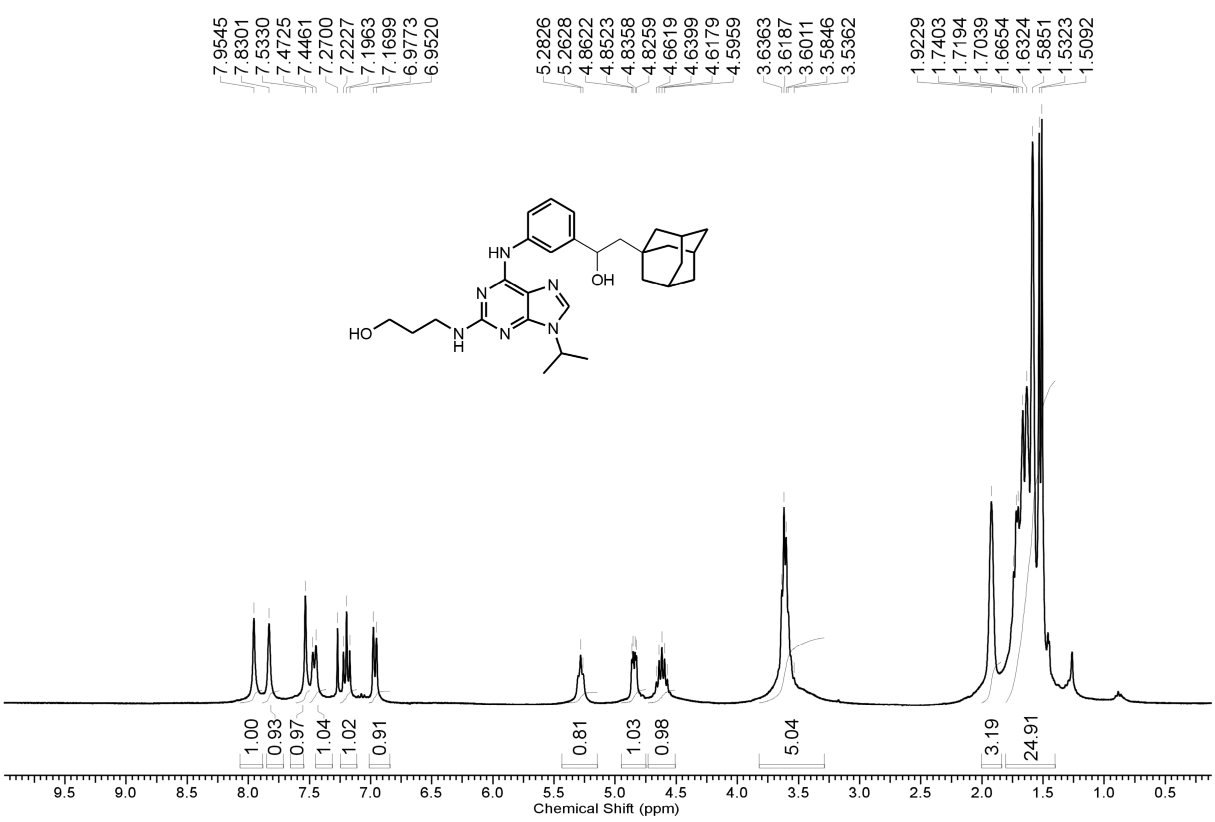

Figure S19 ^{1}H NMR spectrum ($CDCl_3$, 300 MHz, 303 K) of compound **4g**.

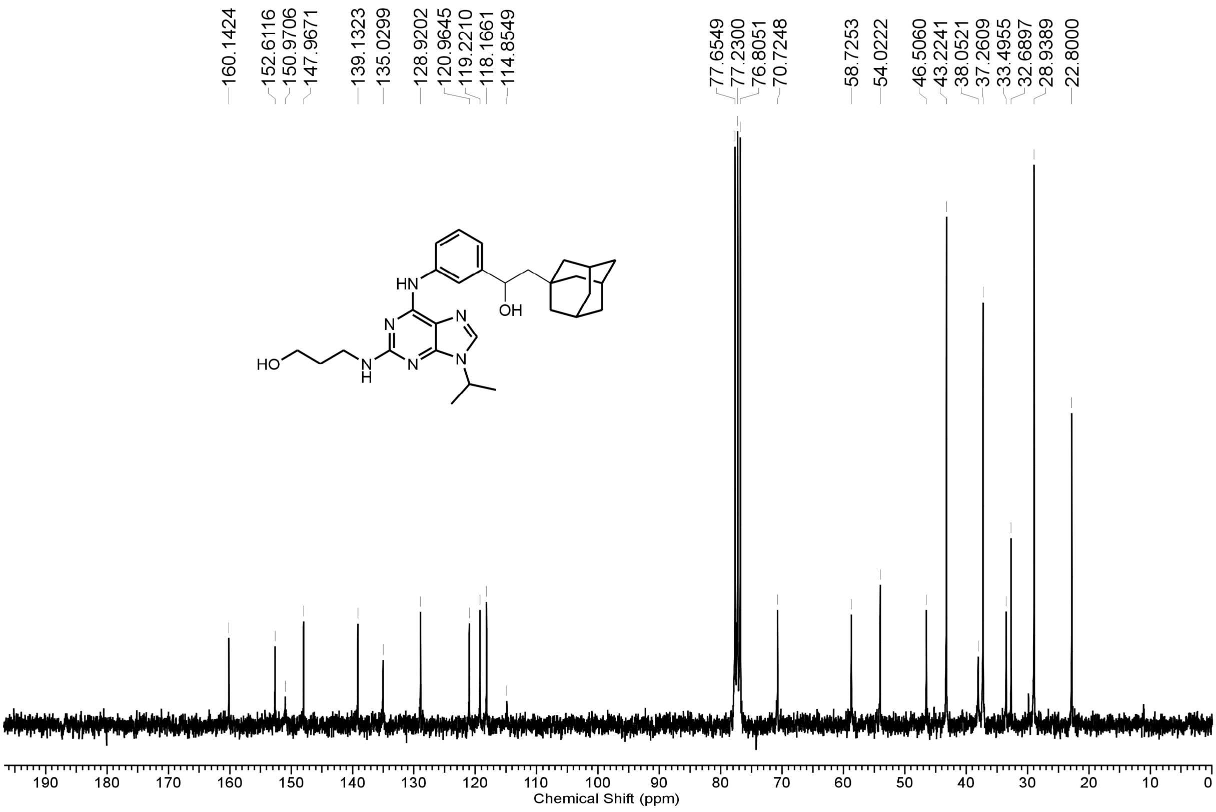

Figure S20 ^{13}C NMR spectrum ($CDCl_3$, 75 MHz, 303 K) of compound **4g**.

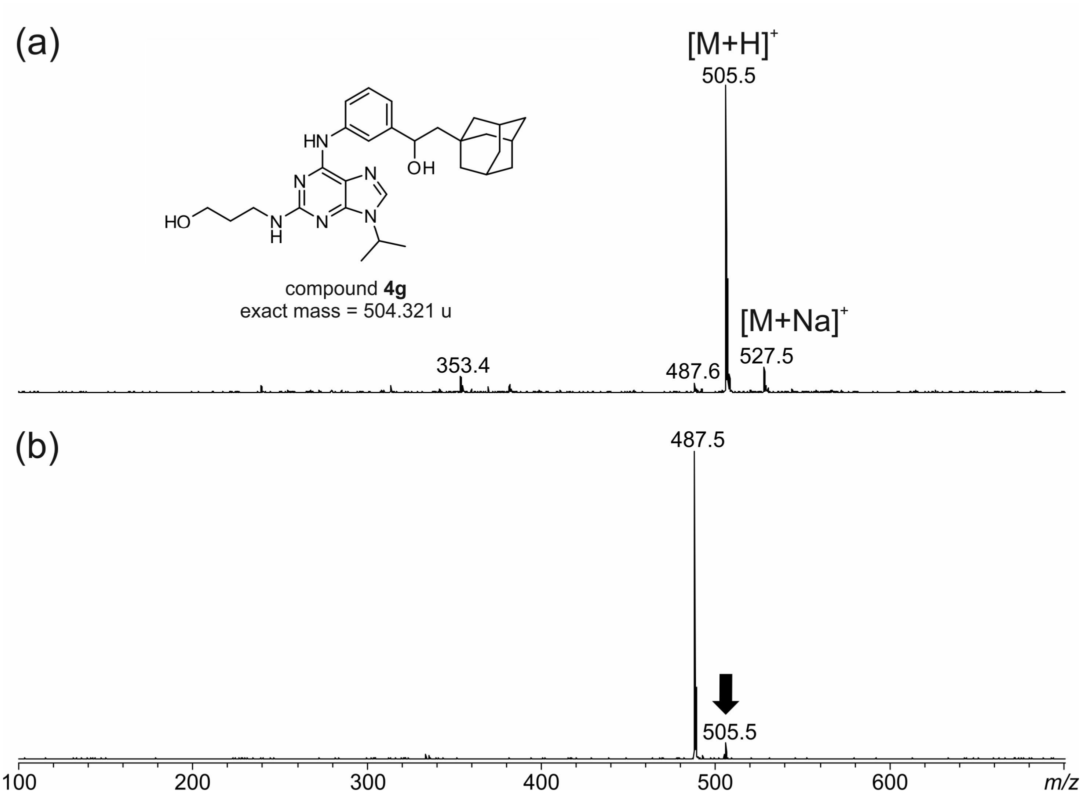

Figure S21 Positive-ion mode ESI mass spectra (full scan) of compound **4g**; (a) first-order mass spectra, (b) MS/MS of *m/z* 505. The assignments for the observed ions are shown in the brackets. The fragmented ion in tandem mass spectrum is marked with bold, downward arrow.

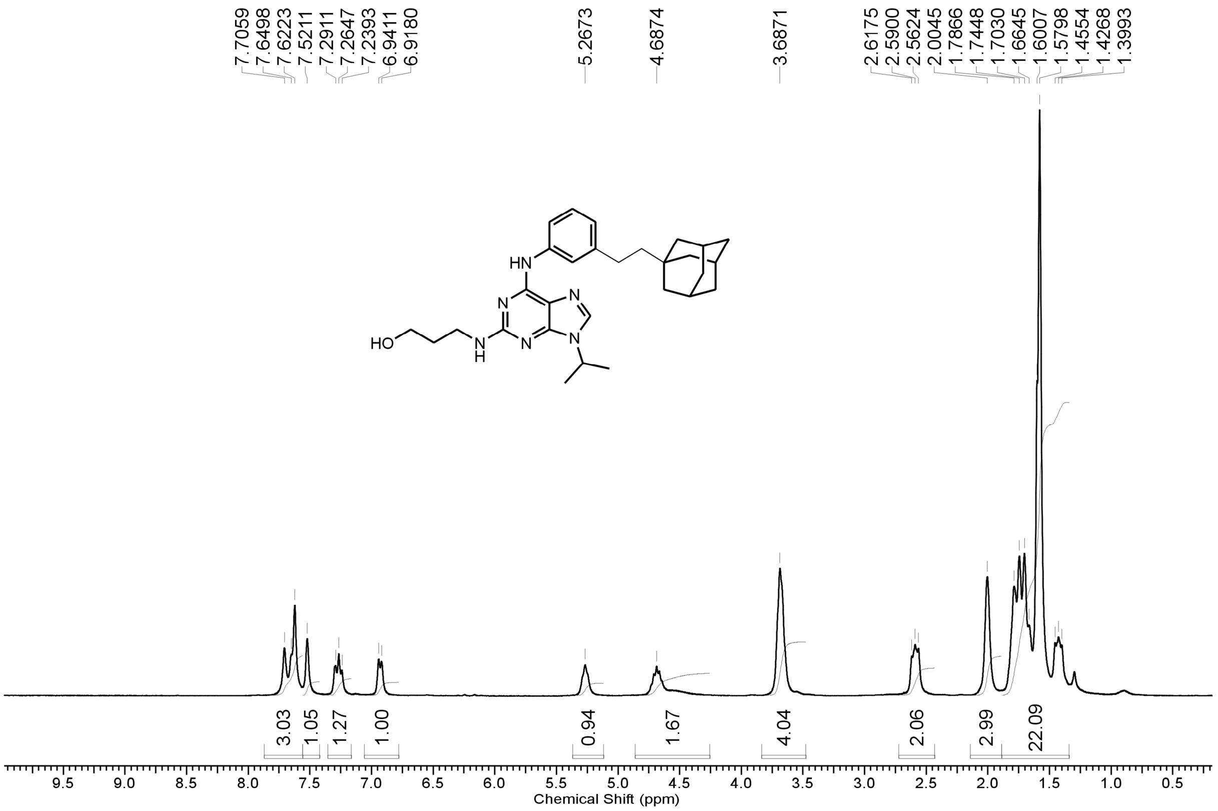

Figure S22 ^{1}H NMR spectrum ($CDCl_3$, 300 MHz, 303 K) of compound **4h**.

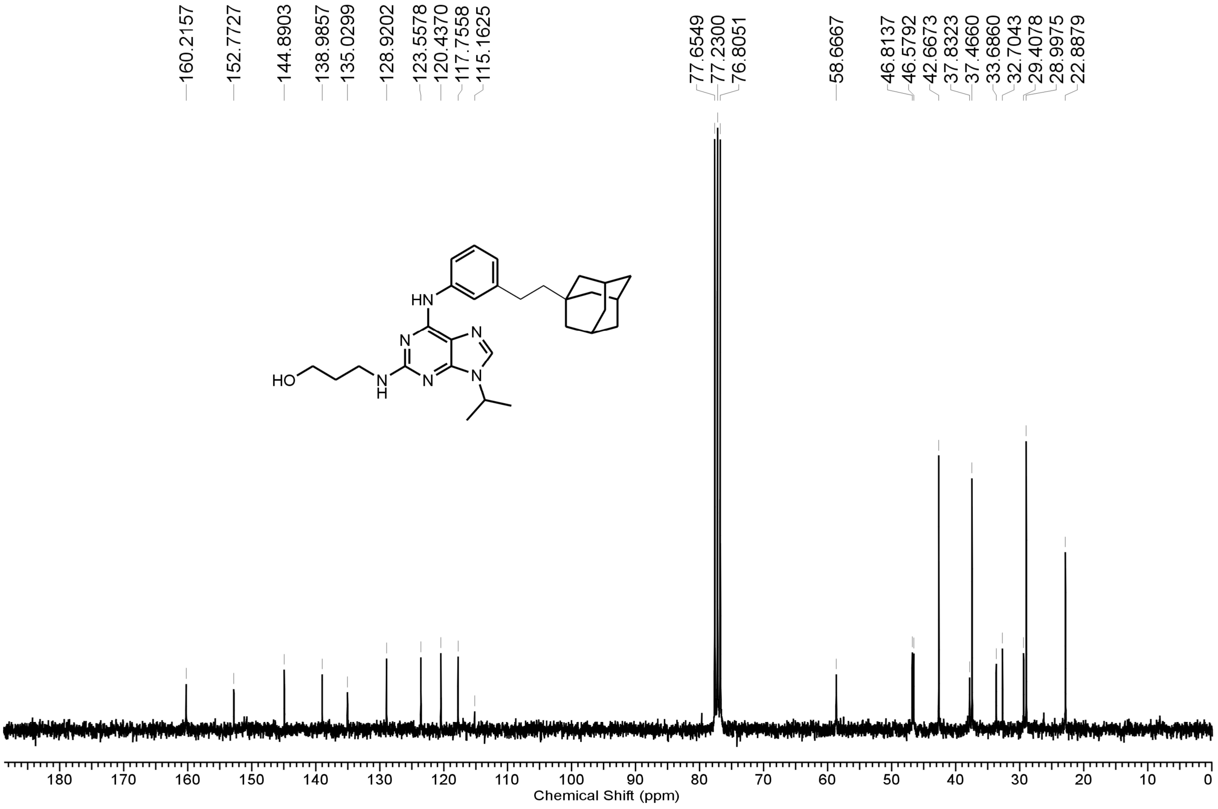

Figure S23 ^{13}C NMR spectrum ($CDCl_3$, 75 MHz, 303 K) of compound **4h**.

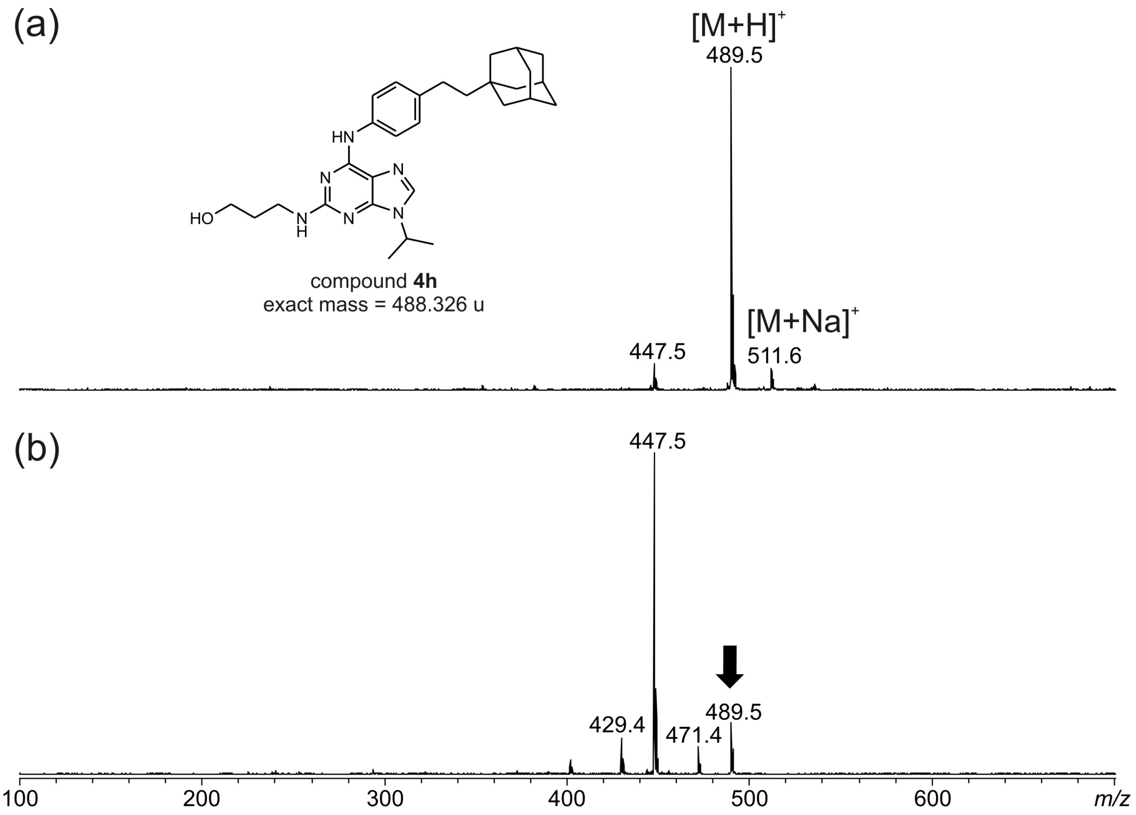

Figure S24 Positive-ion mode ESI mass spectra (full scan) of compound **4h**; (a) first-order mass spectra, (b) MS/MS of *m/z* 489. The assignments for the observed ions are shown in the brackets. The fragmented ion in tandem mass spectrum is marked with bold, downward arrow.

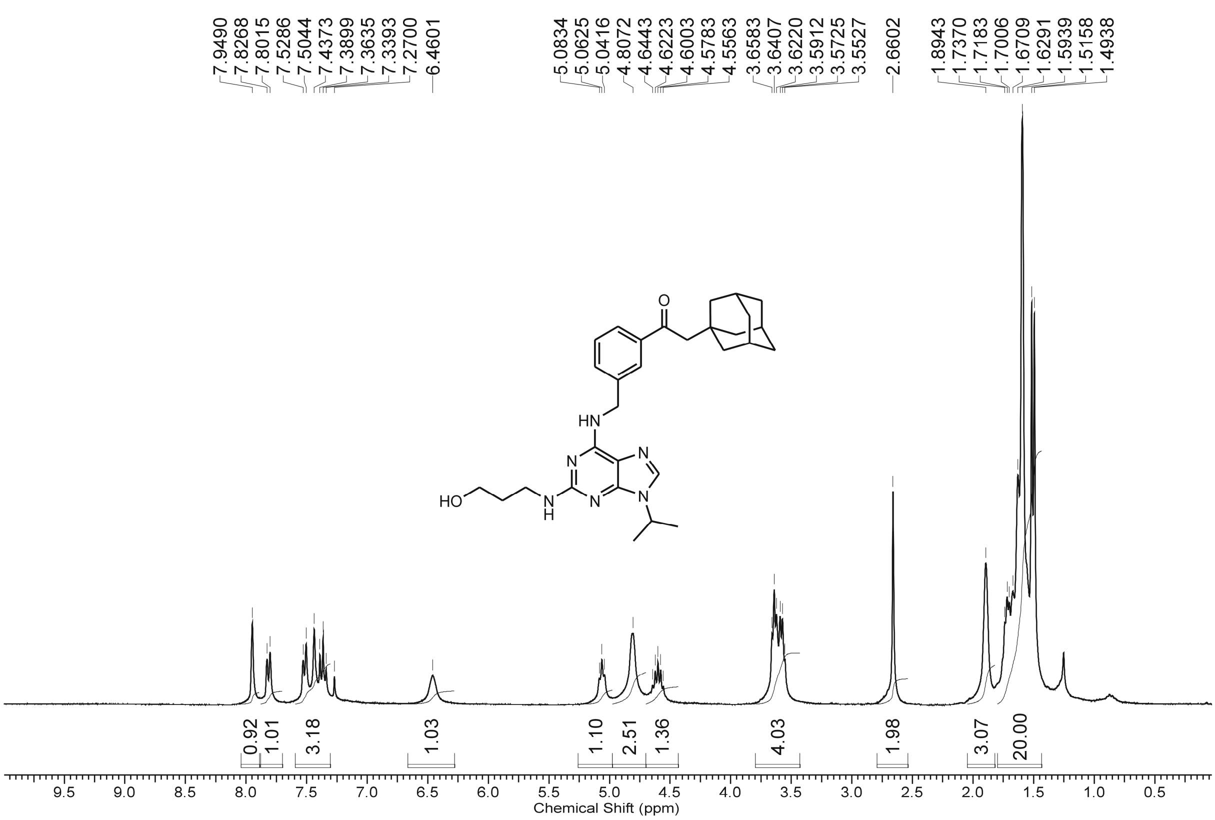

Figure S25 ^{1}H NMR spectrum ($CDCl_3$, 300 MHz, 303 K) of compound **4i**.

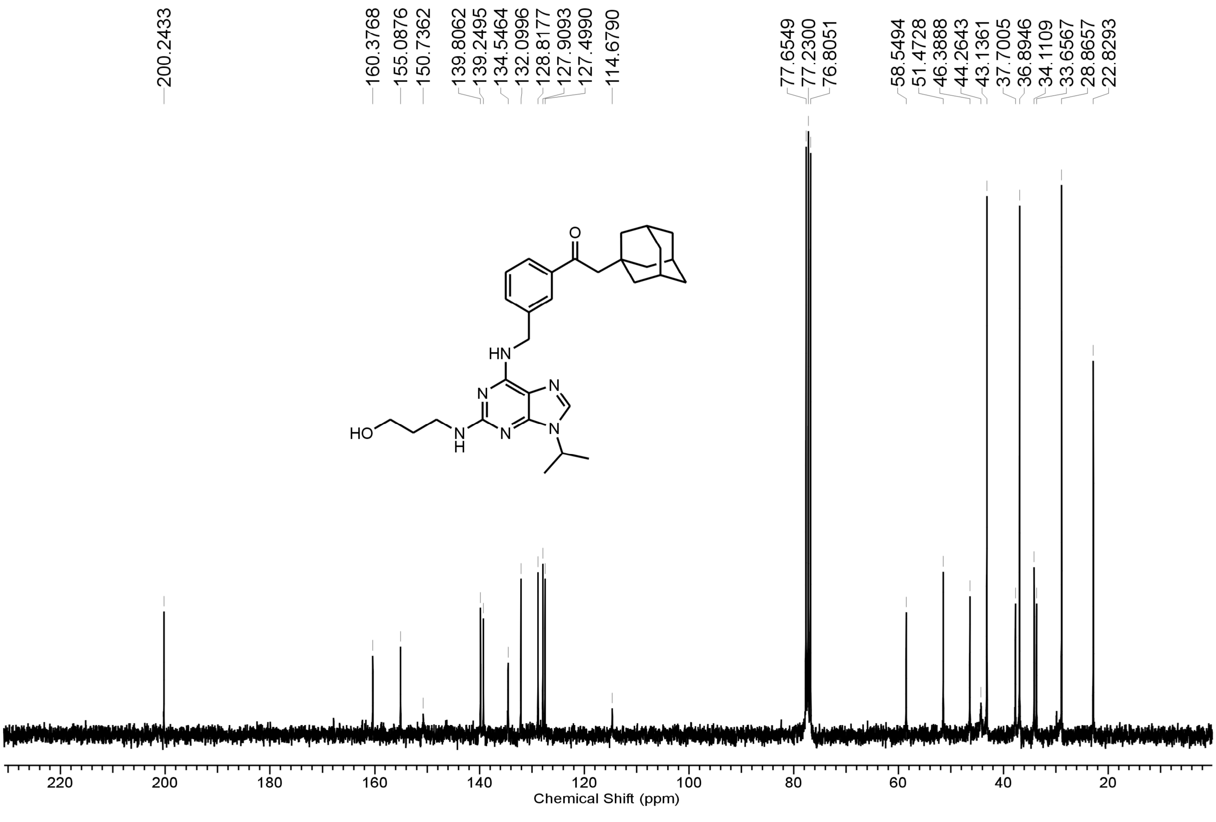

Figure S26 ^{13}C NMR spectrum ($CDCl_3$, 75 MHz, 303 K) of compound **4i**.

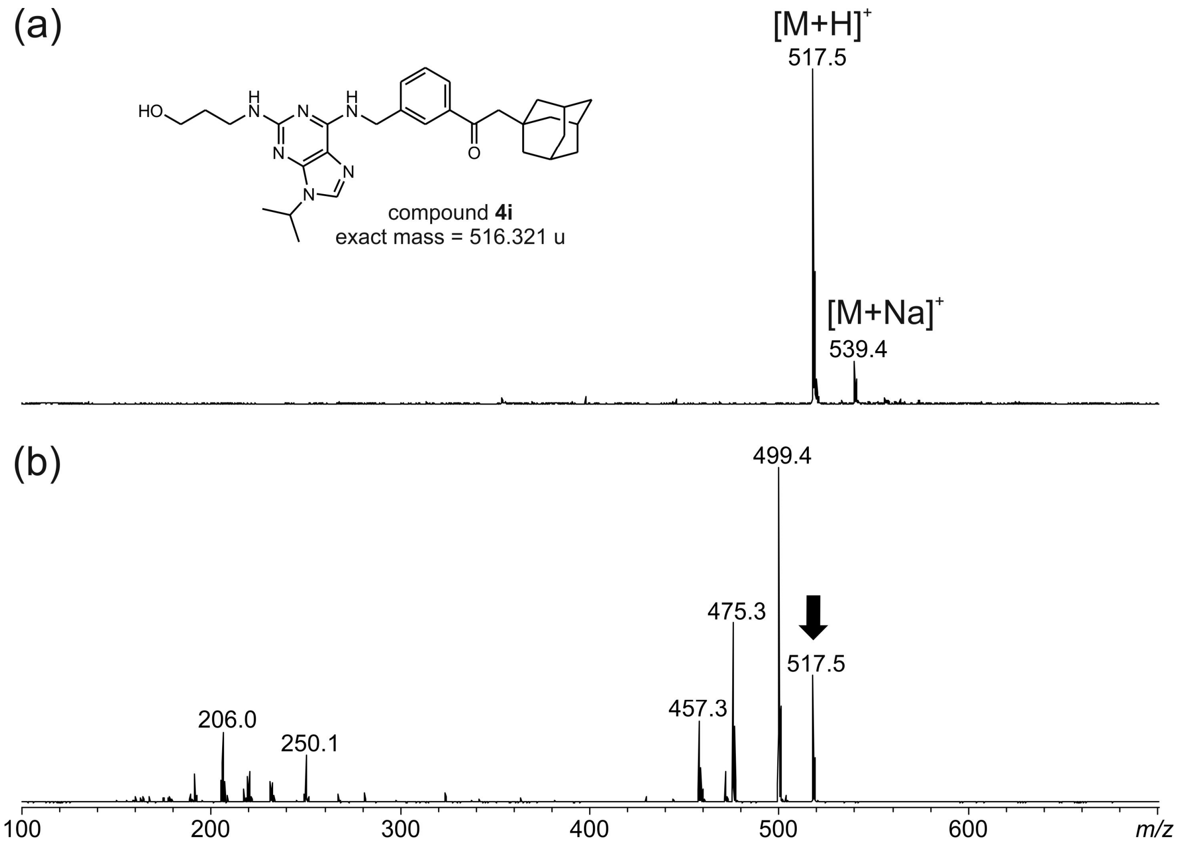

Figure S27 Positive-ion mode ESI mass spectra (full scan) of compound **4i**; (a) first-order mass spectra, (b) MS/MS of *m/z* 517. The assignments for the observed ions are shown in the brackets. The fragmented ion in tandem mass spectrum is marked with bold, downward arrow.

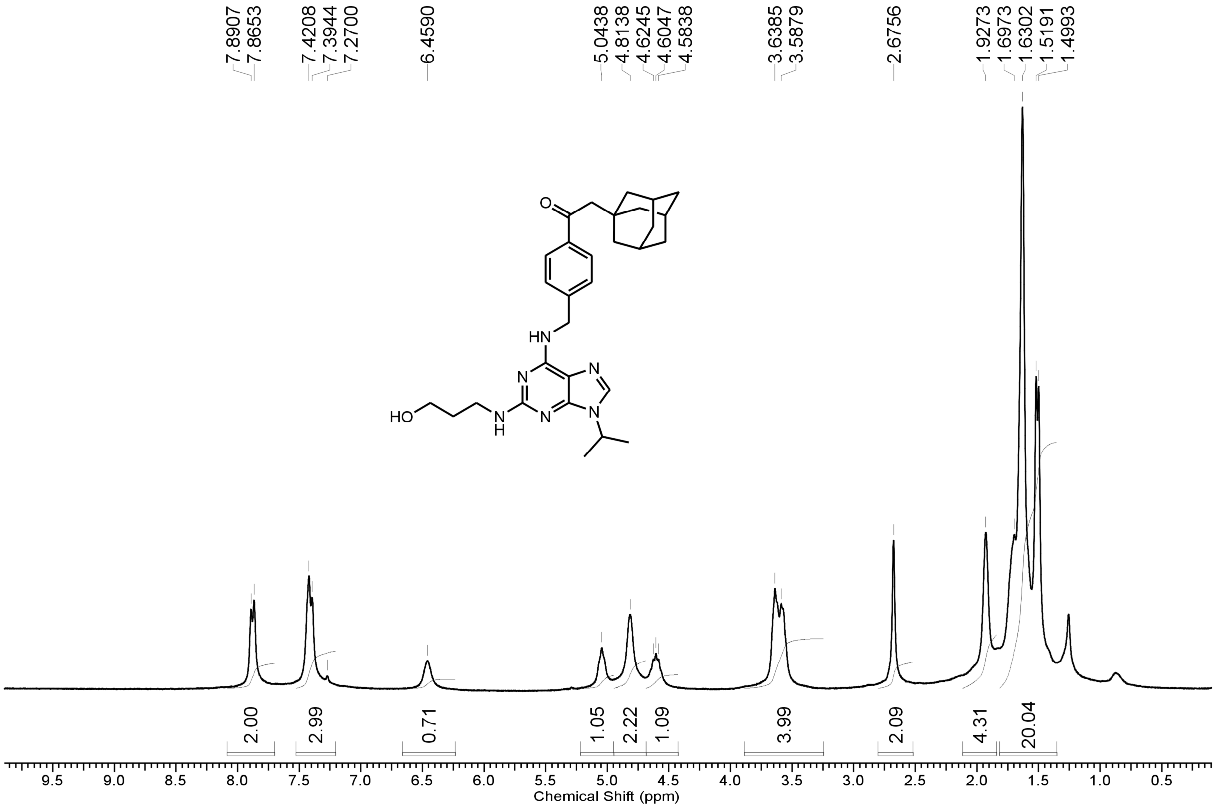

Figure S28 ^{1}H NMR spectrum ($CDCl_3$, 300 MHz, 303 K) of compound **4j**.

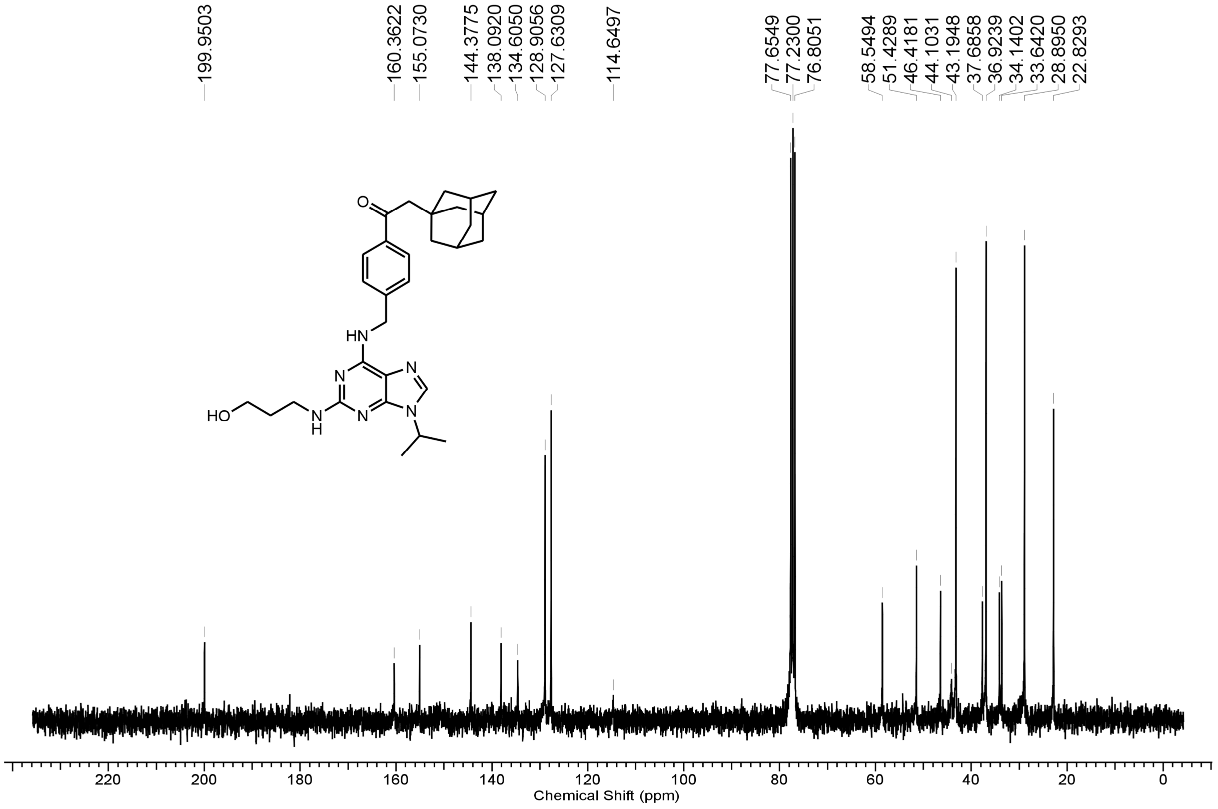

Figure S29 ^{13}C NMR spectrum ($CDCl_3$, 75 MHz, 303 K) of compound **4j**.

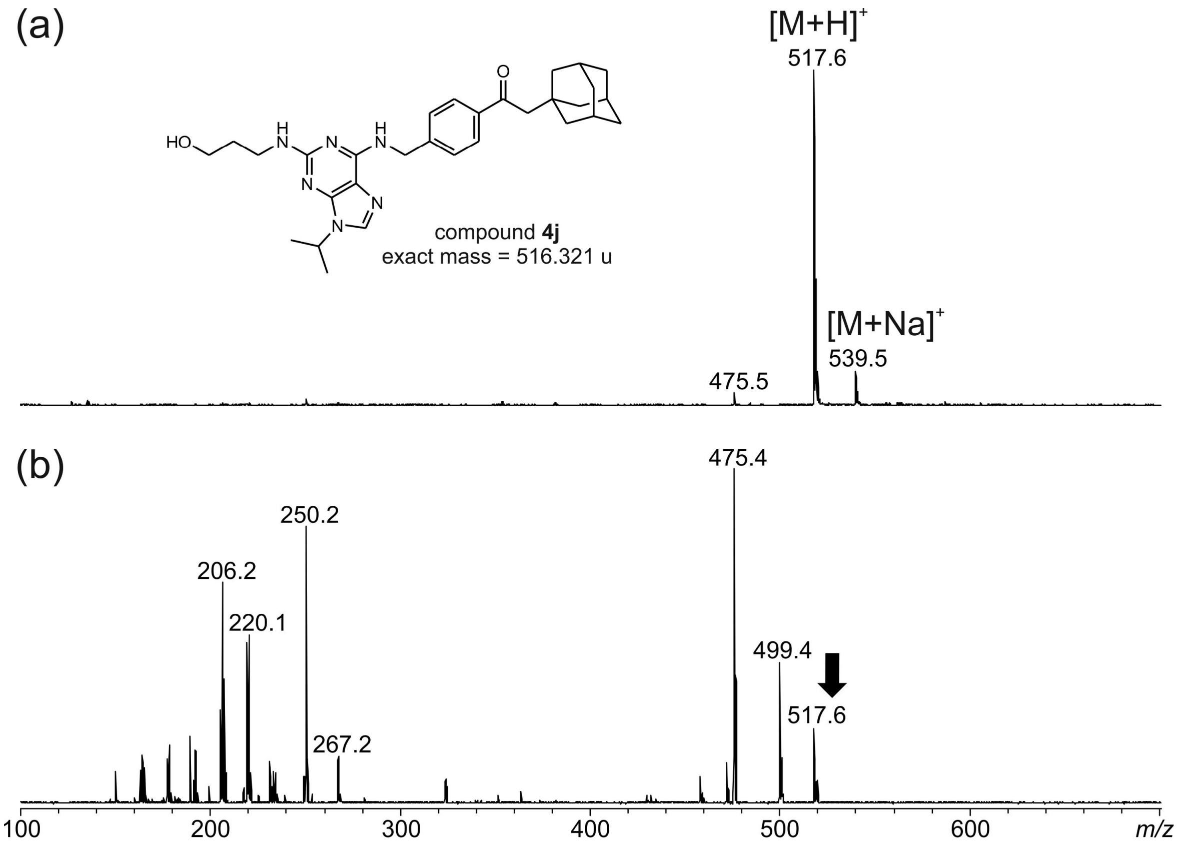

Figure S30 Positive-ion mode ESI mass spectra (full scan) of compound **4j**; (a) first-order mass spectra, (b) MS/MS of *m/z* 517. The assignments for the observed ions are shown in the brackets. The fragmented ion in tandem mass spectrum is marked with bold, downward arrow.

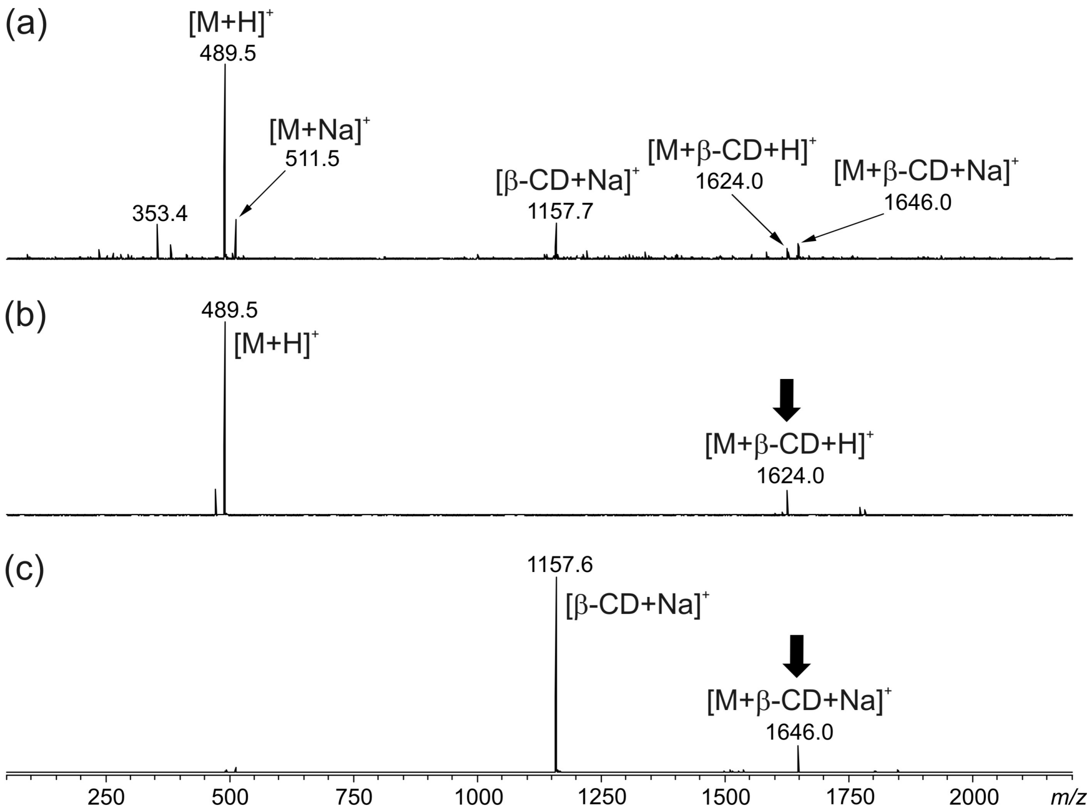

Figure S31 The positive-ion ESI mass spectra of MeOH/H_2O (1/1, v/v) solution of **4a**·β-CD; (a) first-order mass spectra, (b) MS/MS of *m/z* 1624, (c) MS/MS of *m/z* 1646. The assignments for the observed ions are shown in the brackets. The fragmented ions in tandem mass spectra are marked with bold, downward arrows.

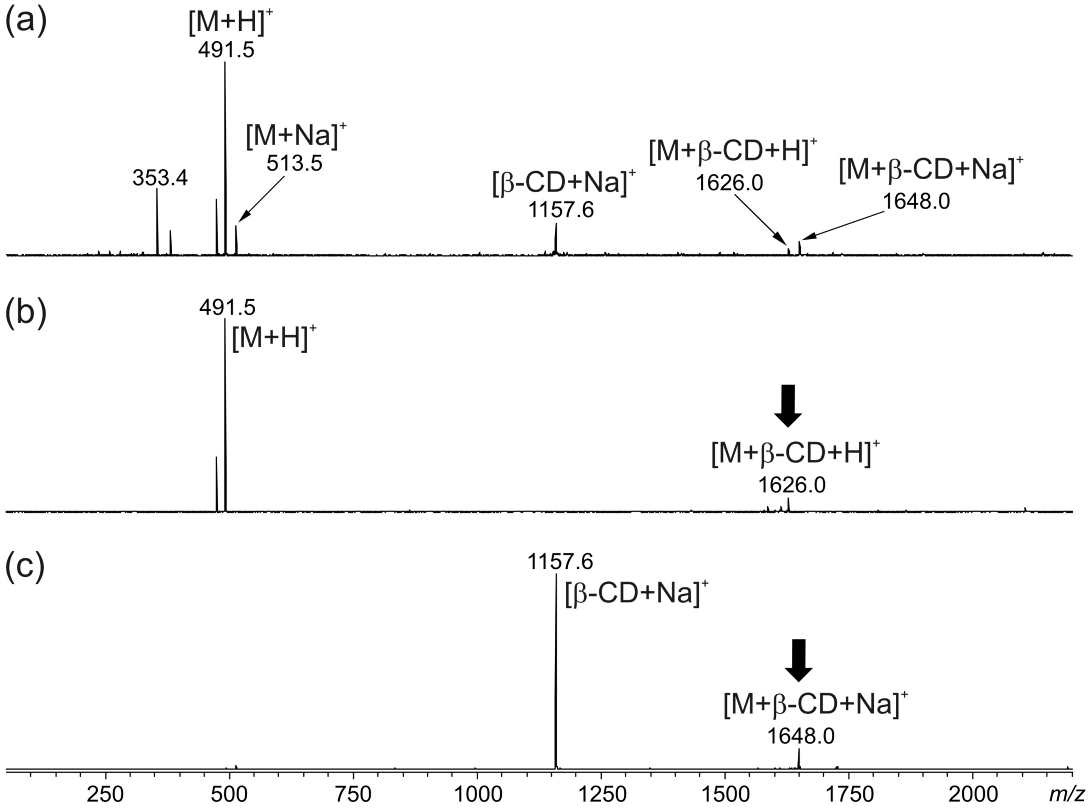

Figure S32 The positive-ion ESI mass spectra of MeOH/H_2O (1/1, v/v) solution of **4b**·β-CD; (a) first-order mass spectra, (b) MS/MS of *m/z* 1626, (c) MS/MS of *m/z* 1648. The assignments for the observed ions are shown in the brackets. The fragmented ions in tandem mass spectra are marked with bold, downward arrows.

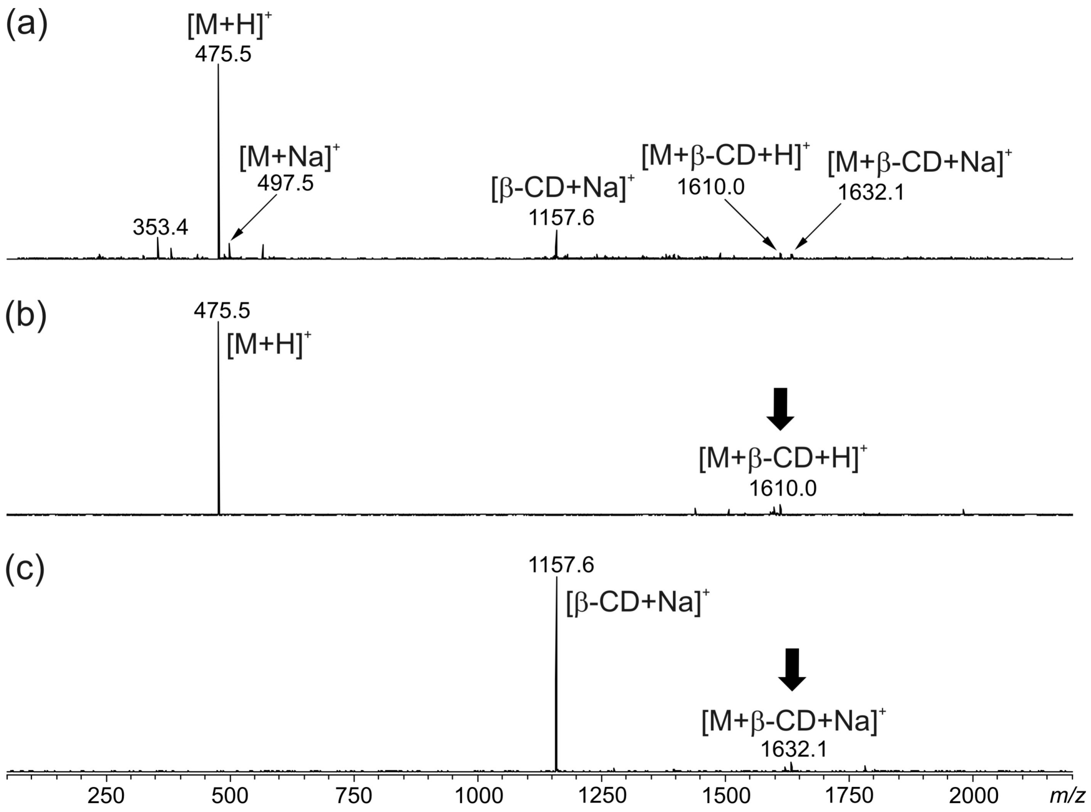

Figure S33 The positive-ion ESI mass spectra of MeOH/H_2O (1/1, v/v) solution of **4c**·β-CD; (a) first-order mass spectra, (b) MS/MS of *m/z* 1610, (c) MS/MS of *m/z* 1632. The assignments for the observed ions are shown in the brackets. The fragmented ions in tandem mass spectra are marked with bold, downward arrows.

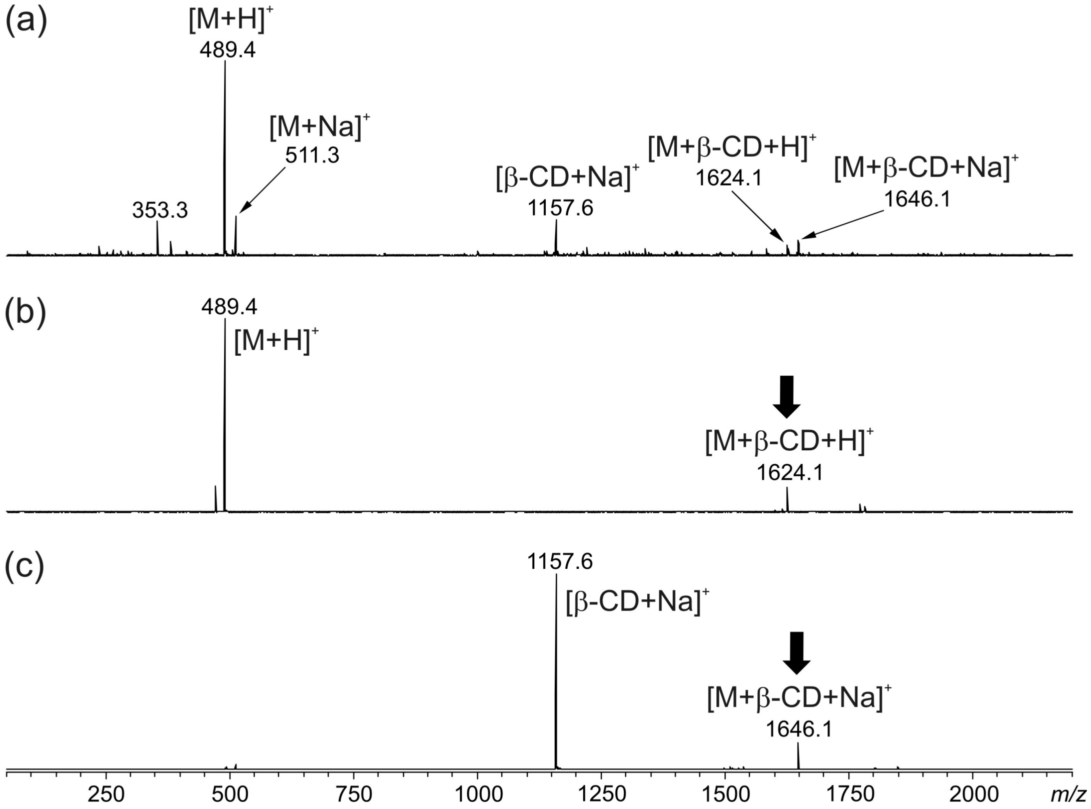

Figure S34 The positive-ion ESI mass spectra of MeOH/H_2O (1/1, v/v) solution of **4d**·β-CD; (a) first-order mass spectra, (b) MS/MS of *m/z* 1624, (c) MS/MS of *m/z* 1646. The assignments for the observed ions are shown in the brackets. The fragmented ions in tandem mass spectra are marked with bold, downward arrows.

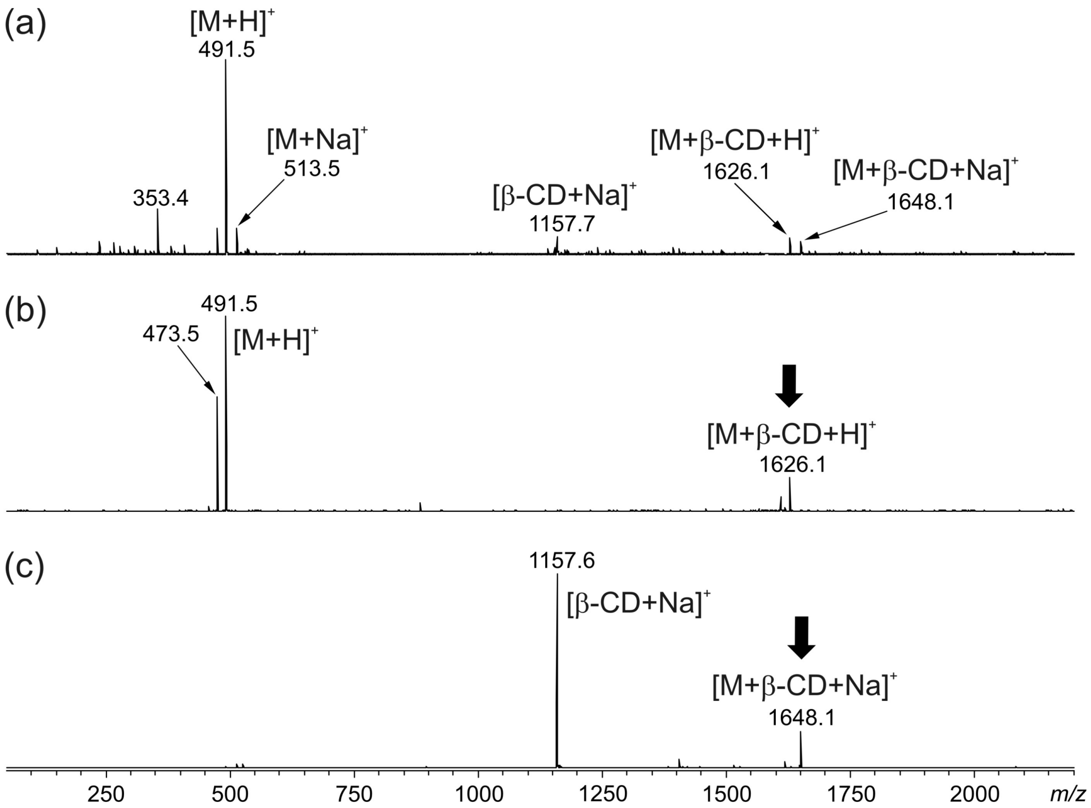

Figure S35 The positive-ion ESI mass spectra of MeOH/H_2O (1/1, v/v) solution of **4e**·β-CD; (a) first-order mass spectra, (b) MS/MS of *m/z* 1626, (c) MS/MS of *m/z* 1648. The assignments for the observed ions are shown in the brackets. The fragmented ions in tandem mass spectra are marked with bold, downward arrows.

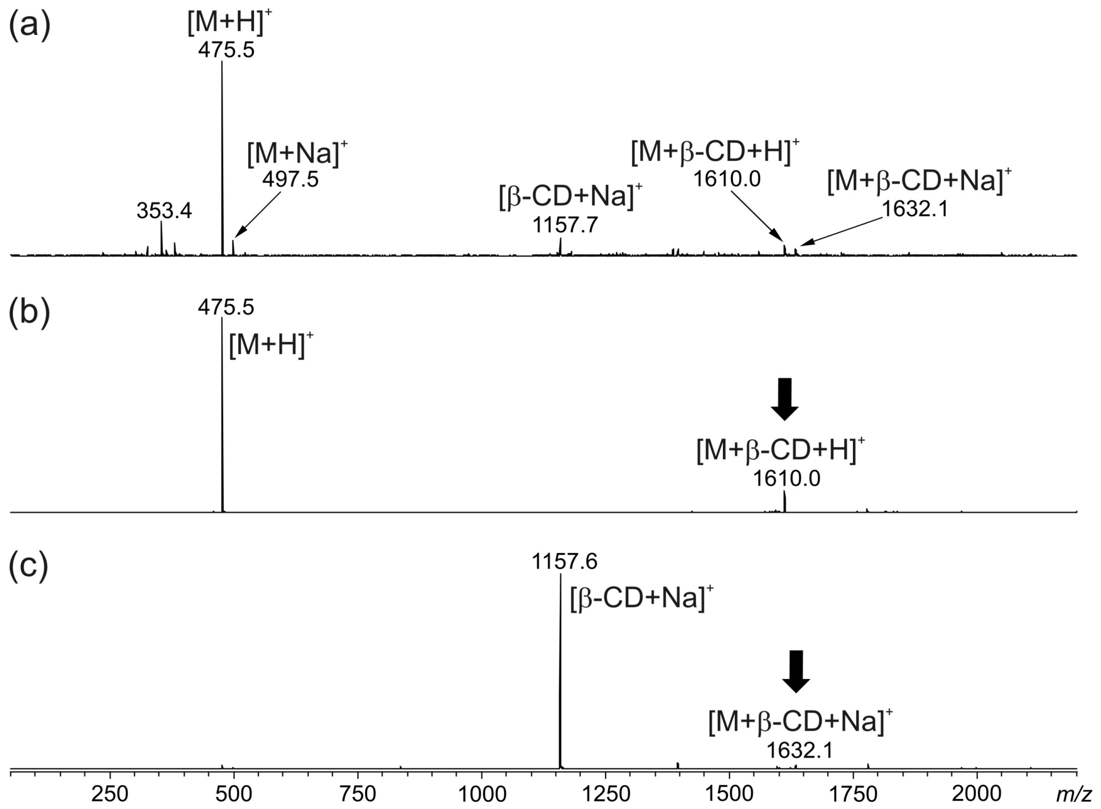

Figure S36 The positive-ion ESI mass spectra of MeOH/H_2O (1/1, v/v) solution of **4f**·β-CD; (a) first-order mass spectra, (b) MS/MS of *m/z* 1610, (c) MS/MS of *m/z* 1632. The assignments for the observed ions are shown in the brackets. The fragmented ions in tandem mass spectra are marked with bold, downward arrows.

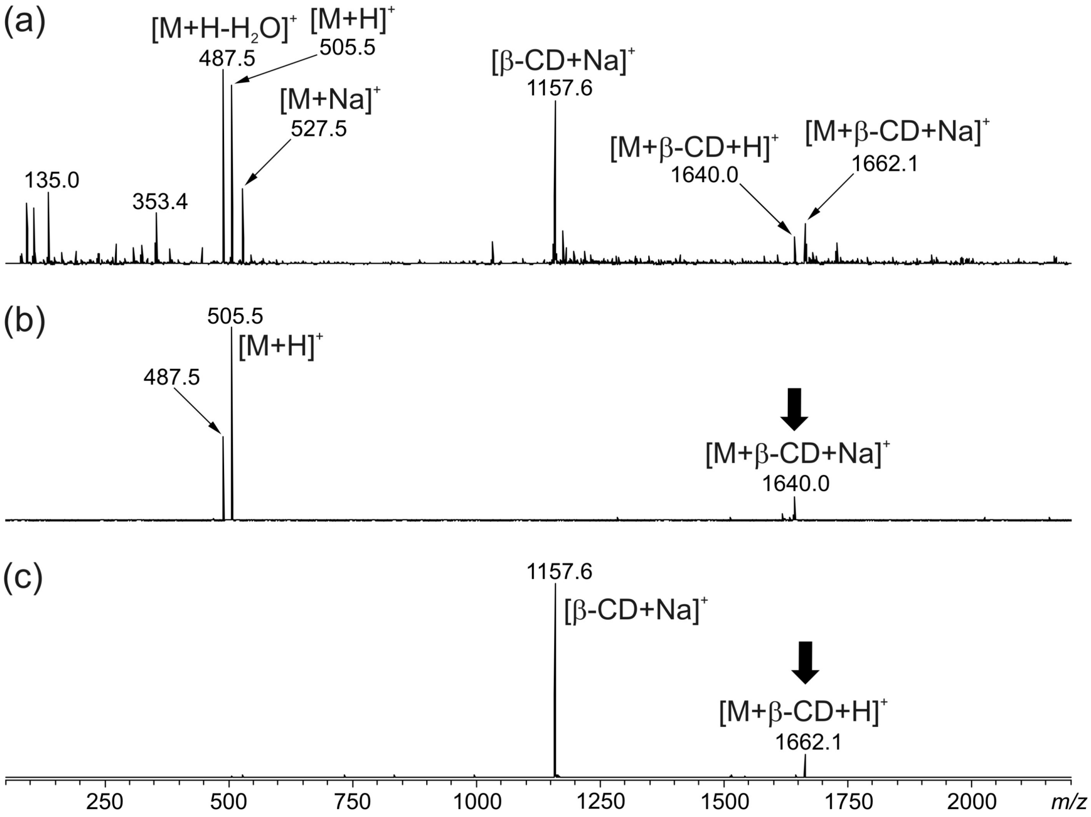

Figure S37 The positive-ion ESI mass spectra of MeOH/H_2O (1/1, v/v) solution of **4g**·β-CD; (a) first-order mass spectra, (b) MS/MS of *m/z* 1640, (c) MS/MS of *m/z* 1662. The assignments for the observed ions are shown in the brackets. The fragmented ions in tandem mass spectra are marked with bold, downward arrows.

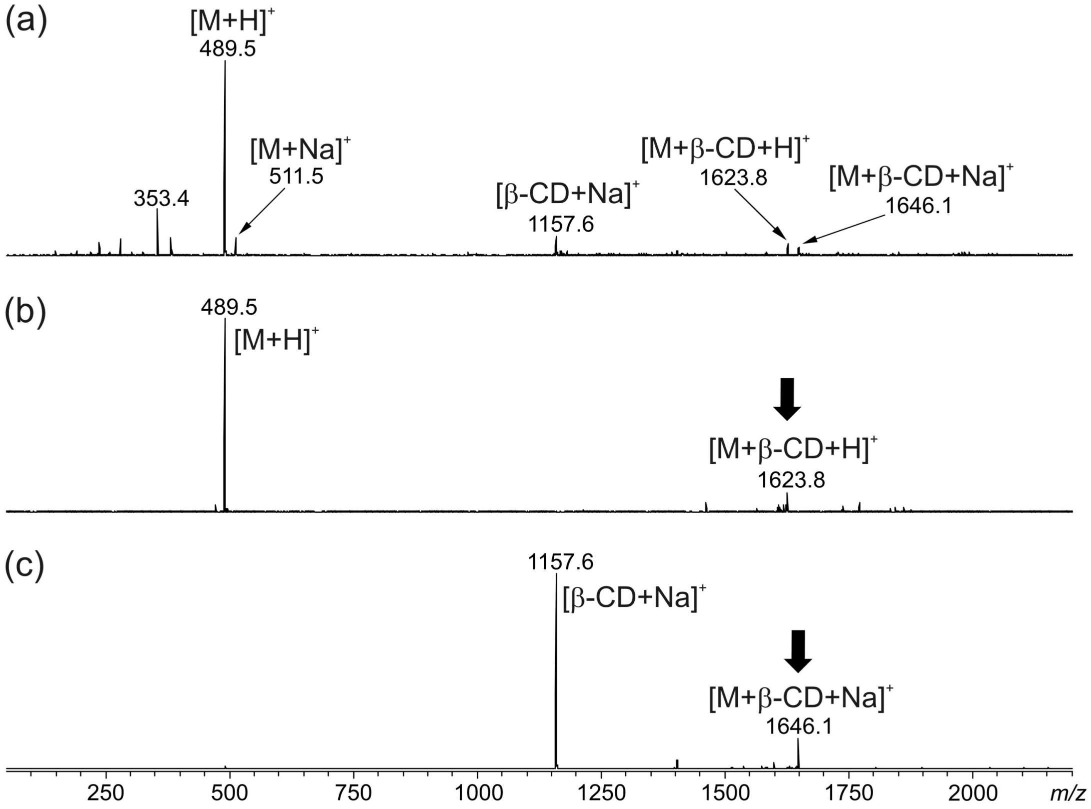

Figure S38 The positive-ion ESI mass spectra of MeOH/H_2O (1/1, v/v) solution of **4h**·β-CD; (a) first-order mass spectra, (b) MS/MS of *m/z* 1623, (c) MS/MS of *m/z* 1646. The assignments for the observed ions are shown in the brackets. The fragmented ions in tandem mass spectra are marked with bold, downward arrows.

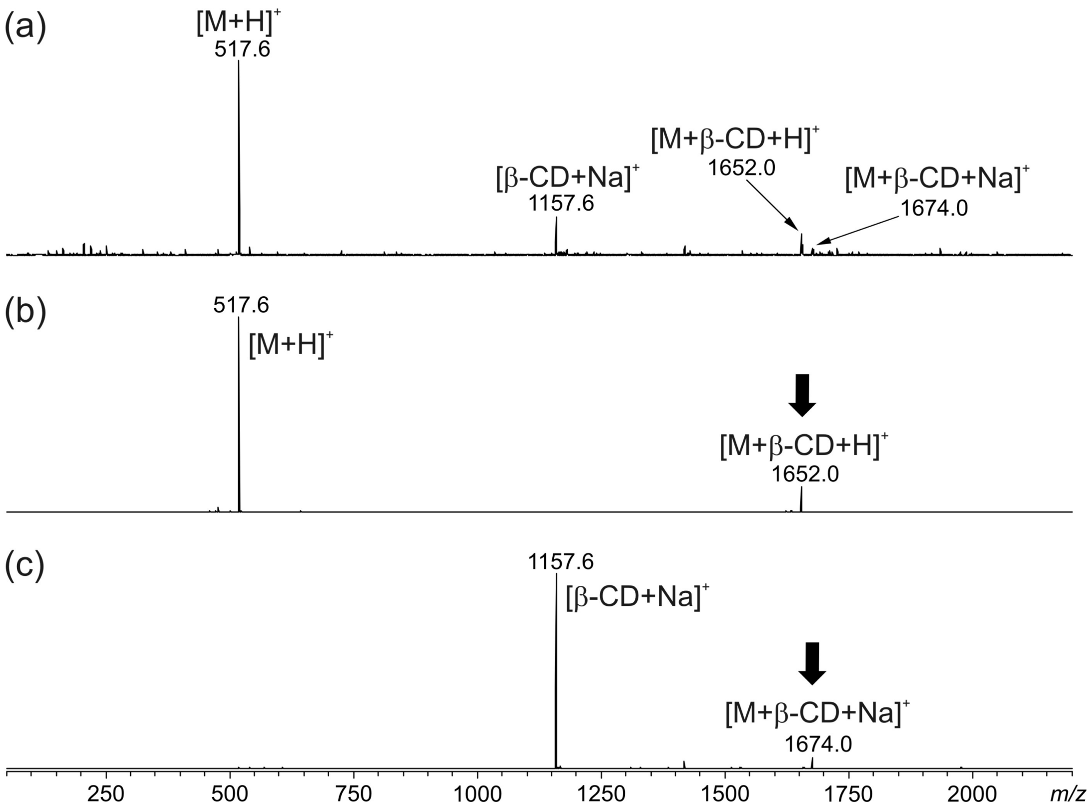

Figure S39 The positive-ion ESI mass spectra of MeOH/H_2O (1/1, v/v) solution of **4j**·β-CD; (a) first-order mass spectra, (b) MS/MS of *m/z* 1652, (c) MS/MS of *m/z* 1674. The assignments for the observed ions are shown in the brackets. The fragmented ions in tandem mass spectra are marked with bold, downward arrows.

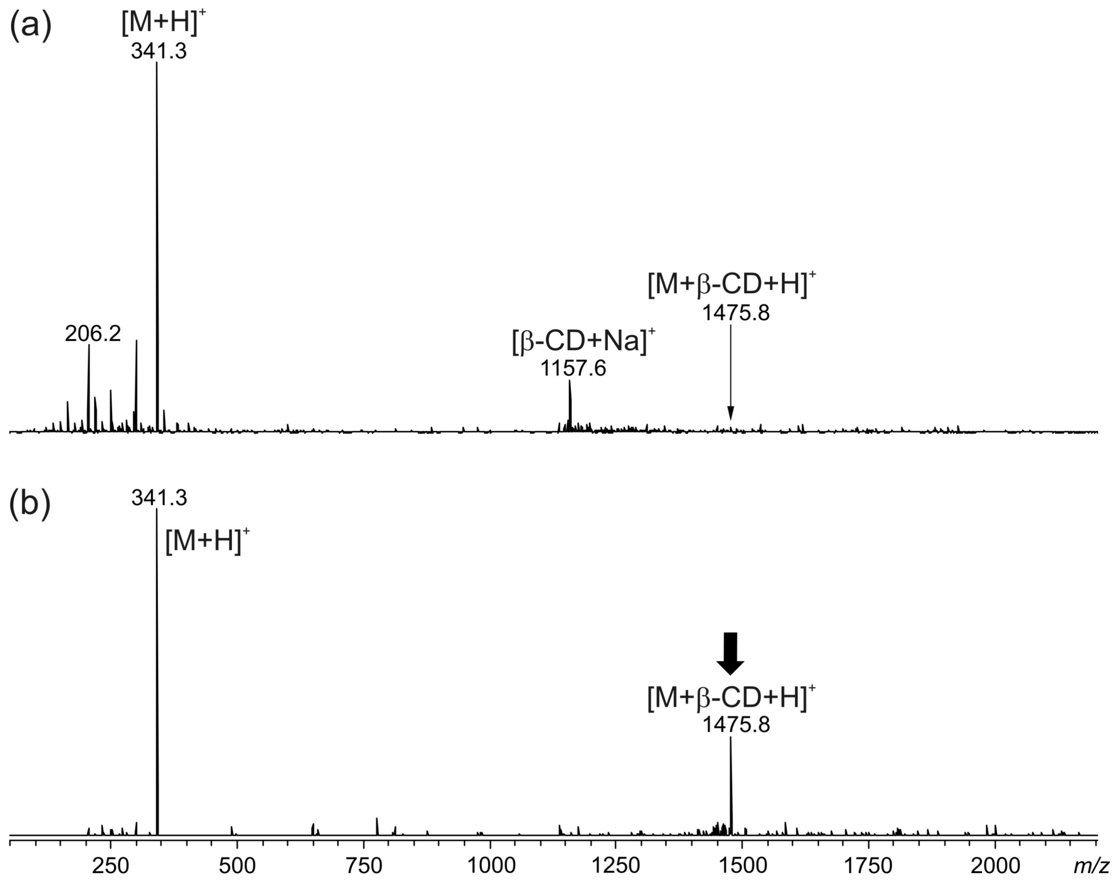

Figure S40 The positive-ion ESI mass spectra of MeOH/H_2O (1/1, v/v) solution of **4k**·β-CD; (a) first-order mass spectra, (b) MS/MS of *m/z* 1475. The assignments for the observed ions are shown in the brackets. The fragmented ion in tandem mass spectra is marked with bold, downward arrow.

Molecular docking results

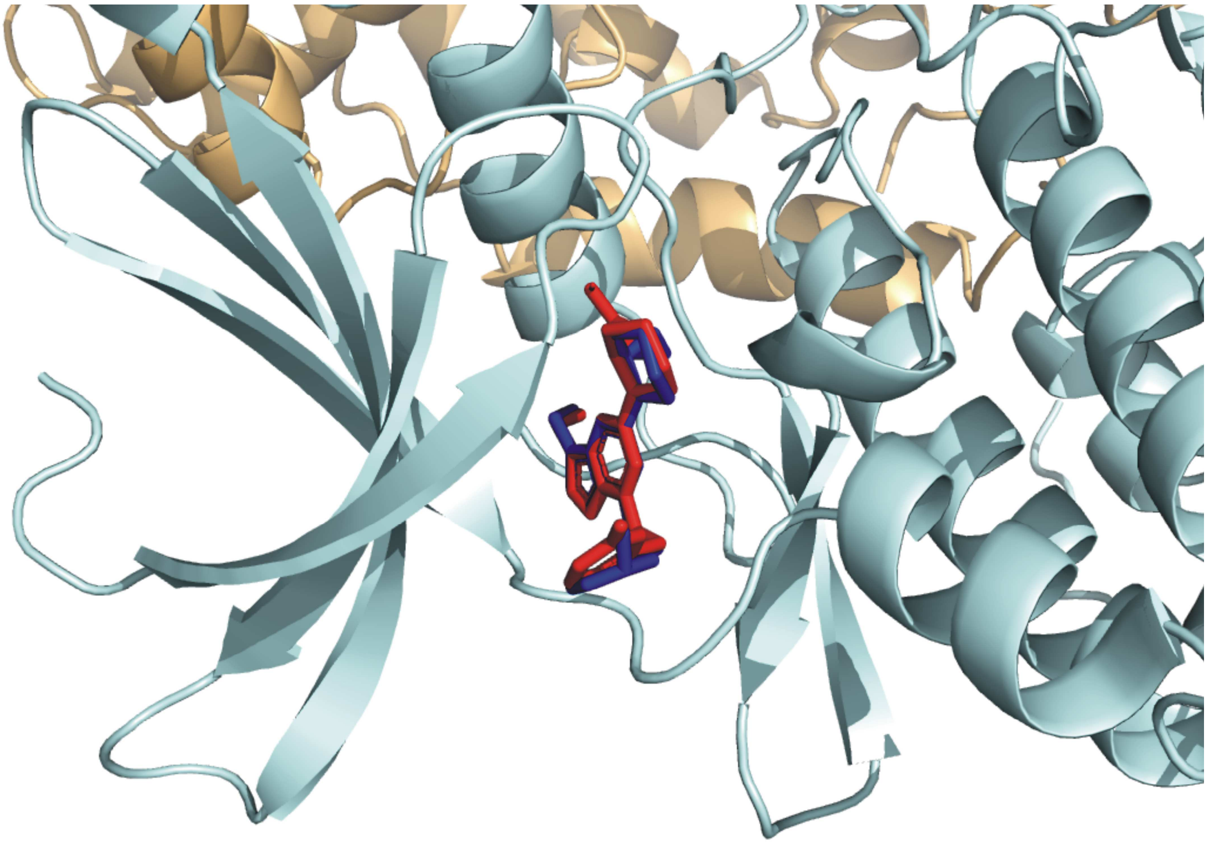

Figure S41 The dinaciclib molecule redocked (red) into X-ray diffraction structure of CDK2 (light blue)/cyclin E (light yellow) complex with dinaciclib (blue).

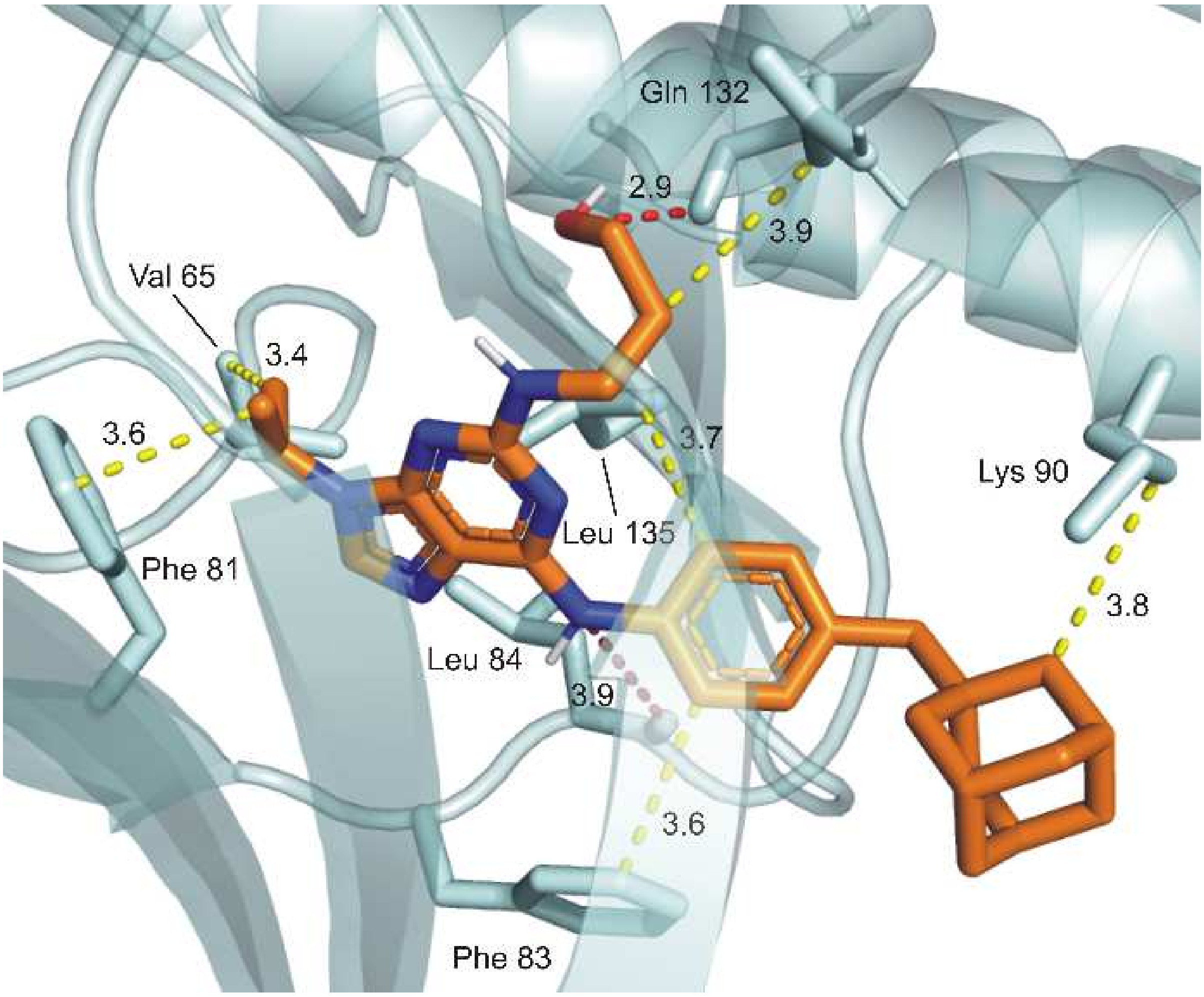

Figure S42 Purine **4f** docked into CDK2/cyclin E active site. Intermolecular contacts were analysed using PLIP. Non-polar contacts and H-bonds are shown as yellow and red dashed lines, respectively. Lenghts are given in Å.

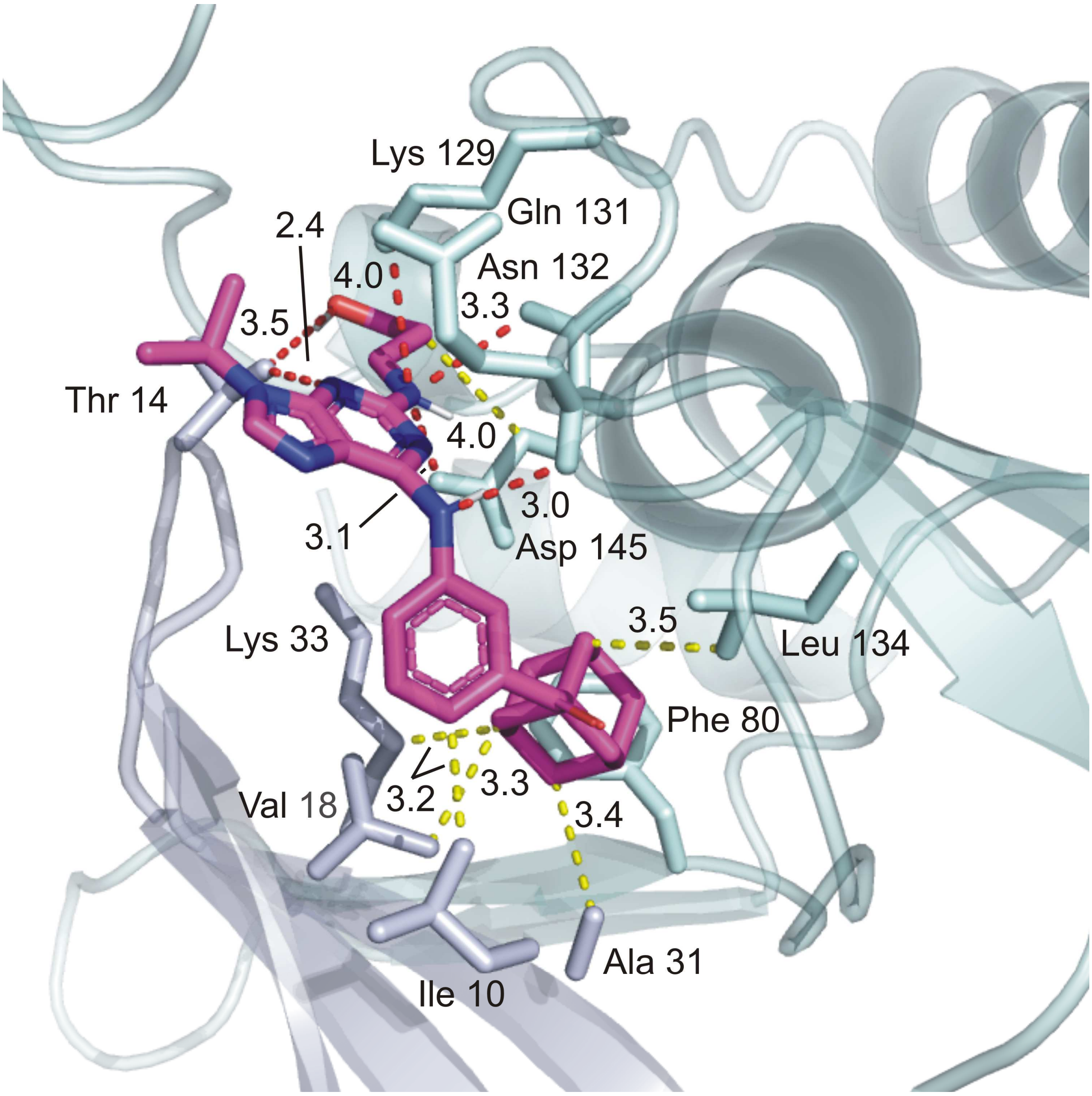

Figure S43 Purine **4a** docked into CDK2 active site. Intermolecular contacts were analysed using PLIP. Non-polar contacts and H-bonds are shown as yellow and red dashed lines, respectively. Lenghts are given in Å.

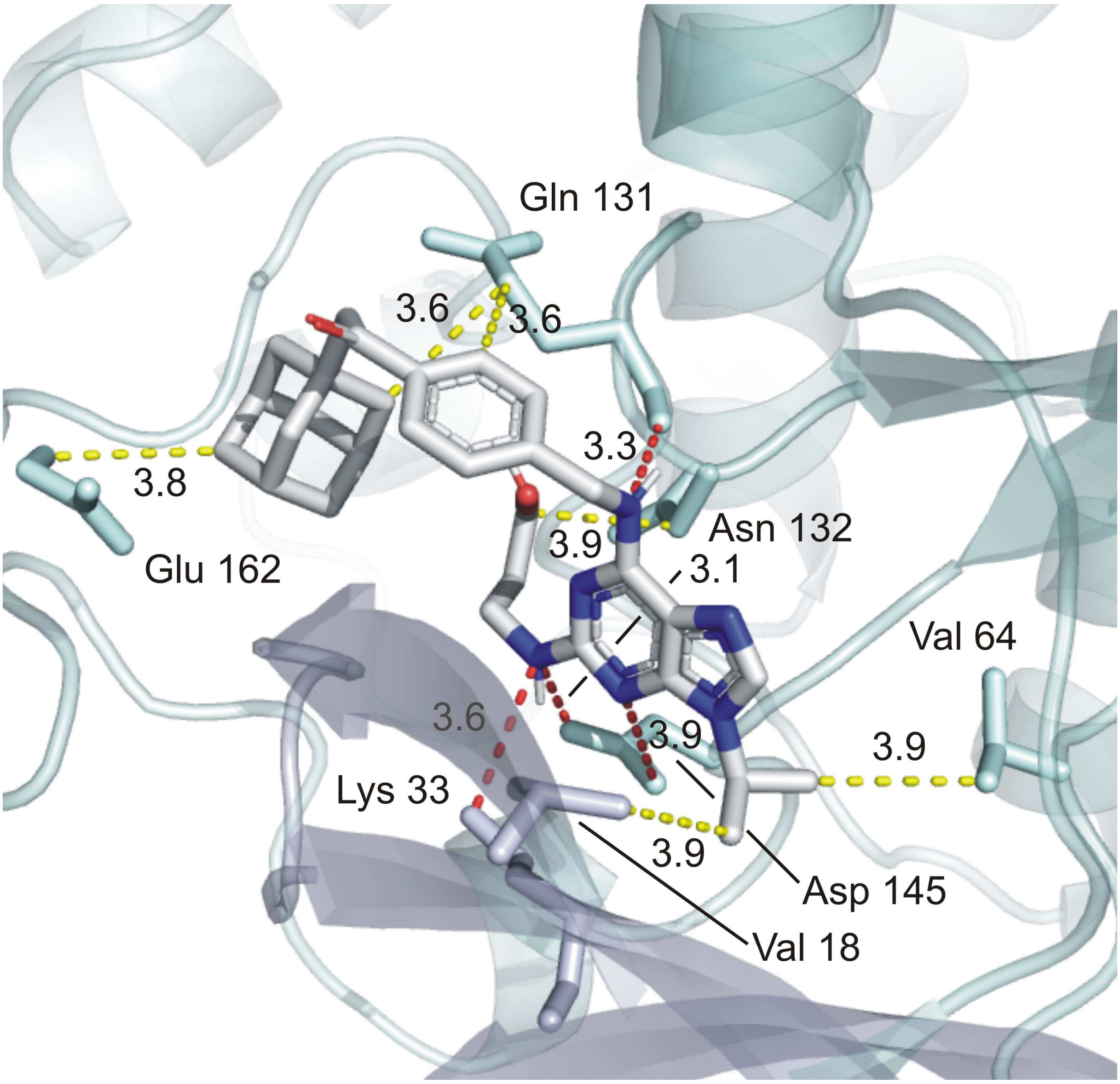

Figure S44 Purine **4j** docked into CDK2 active site. Intermolecular contacts were analysed using PLIP. Non-polar contacts and H-bonds are shown as yellow and red dashed lines, respectively. Lenghts are given in Å.

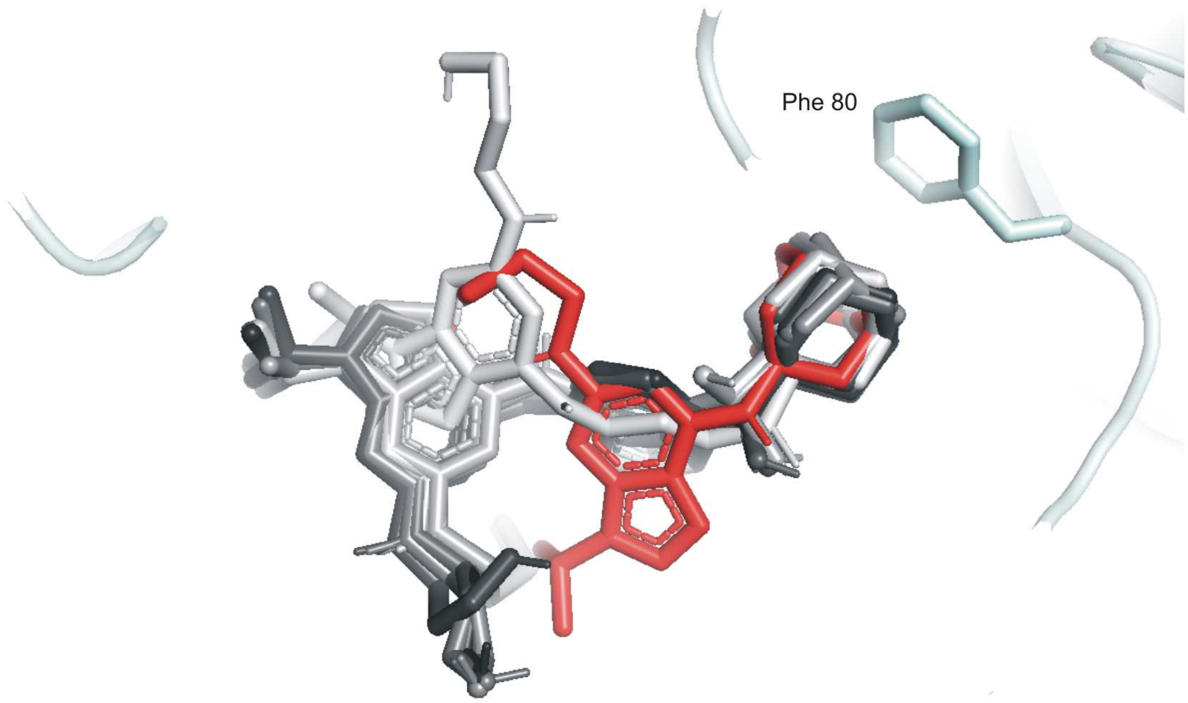

Figure 45 Superposition of the previously published adamantylaminopurine derivative **4m** (red) and the ligands **4a–4f** (grayscale) inside the binding site of CDK2.

Table S1 Intermolecular contacts[a] of purines **4a–4m** in active site of CDK2 as identified using PLIP.

	purine															
	**4a**	(*R*)-**4b**	(*S*)-**4b**	**4c**	**4d**	(*R*)-**4e**	(*S*)-**4e**	**4f**	(*R*)-**4g**	(*S*)-**4g**	**4h**	**4i**	**4j**	**4k**	**4l**	**4m**
–ΔG [kJ mol^{-1}]	44.3	40.1	41.4	41.8	42.2	40.5	40.1	41.4	40.5	40.5	41.0	41.0	37.2	33.4	35.5	35.1
residue	distance [Å]															
Ile 10	3.19	3.58	3.40	3.60					3.69		3.59			3.41<br>3.65		
Gly 11														4.00		
Glu 12			3.60													
Gly 13					3.80	3.91	3.83	3.98								
Thr 14	2.37<br>3.51<br>3.73								2.98<br>3.14							
Val 18	3.30<br>3.95	3.61<br>3.85	3.71<br>3.80	3.62<br>3.66	3.69	3.38<br>3.63	3.52<br>3.70	3.48<br>3.83					3.86	3.61		3.52<br>3.60
Ala 31	3.43								3.60	3.63	3.72					
Lys 33		3.08	3.10	3.39	3.22<br>5.88	3.23<br>5.78	3.17	3.23<br>5.80		3.97	3.97		3.64			3.19
Val 64		3.61		3.79	3.58	3.55	3.58	3.48				3.98	3.86	3.73		3.74
Phe 80	3.22<br>3.57<br>3.75	3.28<br>3.56<br>3.77	3.35<br>3.36<br>3.75<br>3.96	3.48<br>3.63	3.35<br>3.60<br>3.76	3.22<br>3.68<br>3.91	3.46<br>3.49<br>3.98	3.34<br>3.50<br>3.77	3.68	3.63	3.68	3.52<br>3.73		3.53<br>3.79		
Phe 82														3.62		
Leu 83									2.97			2.92		2.94<br>3.72		
His 84									3.77	3.82	3.78					
Gln 85									4.00	3.90	3.90					
Asp 86					3.22		3.05	2.82								
Lys 89																
Lys 129	3.95		3.99													
Gln 131	2.98	3.53		3.59	3.64<br>3.98	3.53	3.96	3.59				3.78	3.33<br>3.60<br>3.90			
Asn 132	3.28												3.87			3.89
Leu 134	3.45	3.51	2.87<br>3.83	3.13	3.22	3.49	3.25		3.77	3.84	3.85					
Ala 144		3.13	3.27	3.18	3.45	3.44	3.26	3.42				3.65				3.20
Asp 145	3.09<br>3.99												3.87<br>3.13			
Glu 162				3.74		3.73		3.85	3.98			3.75	3.82			
Val 163										3.97						
Val 164		3.88	3.79	3.55	3.61	3.84	3.58	3.95	3.53		3.51	3.49				

[a] Non-polar contacts in black (given as C···C distance), H-bonds in red (given as D···A distance), cation-π interactions in blue (given as distance between centre of gravity of aromatic C-atoms and cation).

Table S2 Intermolecular contacts[a] of purines **4a–4l** in active site of CDK2/cyclin E as identified using PLIP.

	purine															
	**4a**	(*R*)-**4b**	(*S*)-**4b**	**4c**	**4d**	(*R*)-**4e**	(*S*)-**4e**	**4f**	(*R*)-**4g**	S-**4g**	**4h**	**4i**	**4j**	**4k**	**4l**	**dinaciclib**
–ΔG [kJ mol^{-1}]		37.6	37.2	36.8	36.8	35.1	39.7	37.6	37.6	39.7	38.0	38.0	39.3	37.3	35.1	41.4
residue	distance [Å]															
Glu 9					3.92	3.37			3.53							
Lys 10						2.89										
Ile 11	2.96	3.55	3.63		3.41	3.25	3.42		3.56		3.53	2.93<br>3.97	3.57<br>3.85			3.62<br>3.76
Gly 12	3.95															
Glu 13									3.94		3.91					3.98
Gly 14															3.27	
Val 19	3.43<br>3.57	3.62	3.68	3.54	3.33<br>3.43	3.13<br>3.29			3.65	3.52<br>3.83	3.63	3.39<br>3.49	3.49<br>3.76	3.70	3.52	3.77
Lys 21					3.39	3.98							3.88			
Ala 32										3.87						
Val 65							3.31	3.38		3.97<br>3.98			3.88	3.72<br>3.73	3.89	
Phe 81	3.55<br>3.69<br>3.76	3.71<br>3.72<br>3.88	3.66<br>3.71<br>3.83	3.62<br>3.64<br>3.77	3.46<br>3.72	3.68<br>3.84	3.41	3.56<br>3.82	3.57<br>3.68	3.49<br>3.58<br>3.71<br>3.96	3.51<br>3.67<br>3.71	3.47<br>3.50	3.44<br>3.49<br>3.72<br>3.90	3.77	3.45<br>3.68	3.76
Glu 82										2.92						
Phe 83	3.71					3.55	3.57	3.59	3.83<br>3.89			3.96	3.76		3.67	3.71
Leu 84		2.44	2.45<br>2.94	2.43<br>3.00				3.93	3.25<br>3.98	2.59<br>3.02	3.36	3.08<br>3.09	2.80<br>4.06		2.97	2.96
His 85				3.98						3.85		3.62				
Asp 87	2.75<br>3.61	2.93	2.99	2.91	2.63<br>4.06	2.69	2.86			2.71<br>3.97		3.27	2.42	3.04<br>3.31<br>3.78		
Lys 90	2.94				3.33	3.19		3.82		2.97		3.57	3.58			
Gln 132				3.99			3.78	3.87<br>2.94		2.99						3.75
Asn 133		3.20	3.94		4.03											
Leu 135	3.09<br>3.84	3.38<br>3.98	3.41	2.93<br>3.60	3.19<br>3.36	3.36<br>3.37	3.60	3.71		2.67		3.38<br>3.56	3.78<br>3.88	3.35		3.64
Ala 145										3.18						
Asp 146										3.84						

[a] Non-polar contacts in black (given as C···C distance), H-bonds in red (given as D···A distance).
